# Understanding low-pressure CO_2_ insertion chemistry in epoxide–CO_2_ copolymerization catalysis

**DOI:** 10.1038/s41557-026-02098-6

**Published:** 2026-04-30

**Authors:** Rosie Thorogood, Katharina H. S. Eisenhardt, Madeleine L. Smith, Charlotte K. Williams

**Affiliations:** Department of Chemistry, University of Oxford, Oxford, UK

**Keywords:** Catalytic mechanisms, Polymerization mechanisms

## Abstract

Despite many CO_2_ use strategies being reliant on fast and selective CO_2_ insertion reactions into metal-alkoxide bonds, in-depth studies into this chemistry remain rare. Here the effect of CO_2_ pressure on the CO_2_ insertion chemistry is studied using epoxide–CO_2_ copolymerizations. Five high-performance literature catalysts are investigated under systematically varied CO_2_ pressures, revealing kinetic profiles indicative of CO_2_ insertion equilibria. For each catalyst, two key parameters describing the CO_2_ insertion chemistry are determined: the equilibrium constant, *K*_eq_, and the saturation CO_2_ pressure above which catalytic performance is maximized, *P*_threshold_. Generalizable correlations between copolymerization activity, *K*_eq_ and *P*_threshold_ are uncovered and used to predict performances for four further catalyst–monomer combinations. These correlations are a direct link between CO_2_ insertion chemistry and process operating conditions, providing a mechanistic framework and testing protocols to accelerate future catalyst development. These results should help deliver efficient scalable CO_2_ use technologies, operating with minimal energy.

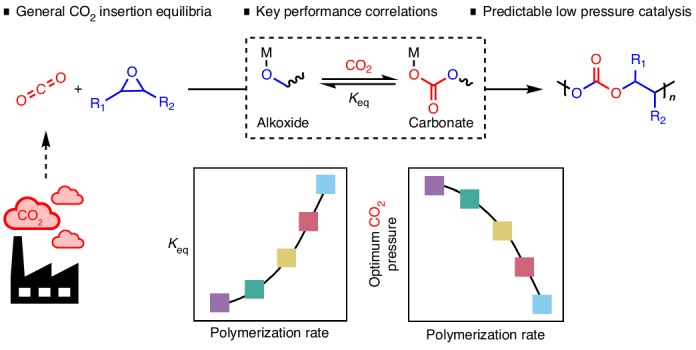

## Abstract (web summary)

CO_2_ insertion into metal-alkoxide bonds is a well-established strategy, but detailed mechanistic studies are lacking. Now the effect of CO_2_ pressure on CO_2_ insertion for epoxide–CO_2_ copolymerization has been explored using a range of catalysts, providing new mechanistic insights as well as testing protocols for monomer and catalyst development.

## Main

The chemical industry remains dependent on unsustainable, fossil carbon sources^1–3^. To move towards a circular carbon economy, there is an urgent need to replace them with renewable carbon, for example from biomass or waste CO_2_, and to ensure the lowest possible energy consumption^1–3^. Scalable CO_2_ use technologies are, therefore, essential for the future chemical industry, but should operate under the lowest feasible pressure and deliver maximum rates^1,4–6^. To effectively transform CO_2_ into valuable products, catalysis is vital^7–10^. CO_2_ insertion into metal-alkoxide bonds (M–OR) is a common step across many catalytic transformations, for example CO_2_ coupling with epoxides to form cyclic carbonates or polycarbonates or with alcohols to form dialkyl carbonates^8,10,11^. In nature, the metalloenzyme Rubisco catalyses atmospheric CO_2_ transformation into 3-phosphoglycerate, involving CO_2_ insertion into a transient Mg(II)ene-diolate (alkoxide) species^5,12^. Related CO_2_ insertions into metal hydroxide or aqua bonds (M–OH or M–H_2_O) are implicated in the industrial high pressure catalytic reverse water gas shift reaction^5^. These CO_2_ insertion steps are often proposed to occur rapidly and to be pre-rate limiting, challenging their direct investigation^9,13,14^. Increasing the scale and efficiency of these CO_2_ transformations requires better understanding of the kinetics and thermodynamics of CO_2_ insertion into metal-alkoxide bonds^15^.

Most previous investigations of CO_2_ insertion chemistry have applied stoichiometric reactions with metal alkoxides and/or hydroxides^16,17^. For example, zinc hydroxide complexes were shown to react with CO_2_ in chemical equilibria^18,19^. Whilst stoichiometric studies can be useful, caution should be applied in generalizing their findings to catalytic processes. Such CO_2_ insertions relevant to utilization catalysis should be studied under real process operating conditions.

**Fig. 1:**
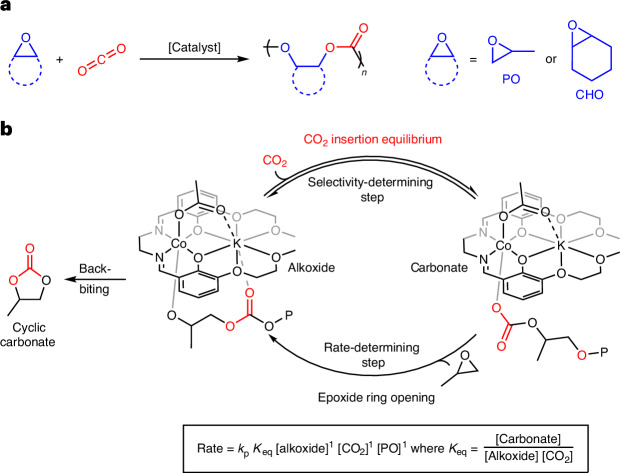
The ROCOP of epoxides and carbon dioxide. **a**, ROCOP of epoxides and CO_2_ with the two most commonly studied epoxides: propene oxide (PO) and cyclohexene oxide (CHO). **b**, The proposed catalytic cycle for a high-performance Co(III)K(I) catalyst. The CO_2_ insertion is proposed as an equilibrium and incorporated into the rate law^13^.

In this work, we target carbon dioxide and epoxide ring-opening copolymerization (ROCOP) using catalysts that produce perfectly alternating polycarbonates and maximize carbon dioxide uptake^20,21^. These catalytic cycles also involve metal-alkoxide intermediates that react with carbon dioxide to produce a metal-carbonate species; in this field it is desirable to identify carbon dioxide insertion chemistry that is effective at low catalyst (metal-alkoxide) concentrations and low CO_2_ pressures. Previous kinetic and computational investigations, using a range of different catalysts, were generally conducted using higher carbon dioxide pressures (10–30 bar) and nearly always showed a zero order in CO_2_ pressure; as a consequence, in this field catalysts were not designed or optimized for low CO_2_ pressure insertion chemistry^13,14,22–25^. Very recently, we discovered the ROCOP of CO_2_ and propene oxide (PO) using a heterodinuclear Co(III)K(I) catalyst, which showed good activity and selectivity at very low pressures. The catalyst also showed rates that depend on the CO_2_ pressure applied over the range 2–12 bar. At higher pressures (12–30 bar), the rates are independent of pressure and are maximized^13^. Experimental kinetics and density functional theory calculations suggested that CO_2_ insertion occurs by an equilibrium reaction between metal-alkoxide and metal-carbonate intermediates^13,14^ (Fig. 1b). The CO_2_ insertion equilibrium controls the effective concentration of the metal-carbonate intermediate, which is the catalytic species involved in the polymerization rate-determining step. Further, it influences catalyst selectivity, as the cyclic carbonate by-product forms from the metal alkoxide^13^. Before our work, only one other group reported two β-diiminate Zn(II) catalysts, used for cyclohexene oxide (CHO)–CO_2_ ROCOP, that also showed pressure-dependent rates^26,27^. These previous reports, together with some suggestions of CO_2_ pressure-dependent rates in other CO_2_ insertion reactions^18,19,28^, motivate the current in-depth investigation into how CO_2_ pressure influences rates more generally in this field of copolymerization catalysis.

For industrially operable polymerizations, energy efficiency is important and this may be achieved by conducting reactions at moderate temperatures so as to balance rates, selectivity and polymer viscosity^29^. Further, reactions should be conducted at the lowest catalyst loadings feasible, particularly if residues must be removed before polymer use. In the past, there have been extensive investigations into the influences of catalyst loading and process temperature but much less understanding of how carbon dioxide pressure influences the catalysis^14,21,30,31^. Nonetheless, when copolymerization catalysts are effective at low carbon dioxide pressures (*P*_CO2_ ≤ 10 bar), the resulting process financial costs (capex) and greenhouse gas emissions associated with gas compression are expected to be substantially lower^29^. To illustrate this point, Aspen Plus was used to estimate the changes to cost and energy input during poly(propene carbonate) (PPC) production due to CO_2_ compression from 1 bar to 5 bar, 10 bar, 20 bar or 50 bar, respectively. These estimates suggest that increasing the CO_2_ pressure from 5 bar to 20 bar, results in an increase of ~50% to the capital cost and >200% to the process energy requirement (Supplementary Table 1). These results highlight the economic and environmental benefits for lower pressure copolymerization catalysts.

Here the CO_2_ insertion chemistry occurring during CO_2_ and epoxide ROCOP catalysis is examined under industrially relevant process operating conditions, that is low catalyst loading (1:4,000 catalyst:epoxide), moderate temperature (50 °C) and minimized but fixed carbon dioxide pressure^9,20,32^ (Fig. 1a). Epoxide–CO_2_ ROCOP is an efficient CO_2_ utilization process, forming useful polycarbonate products with high CO_2_ uptakes, for example 43 wt% CO_2_ in PPC^9,13,33–36^. These alternating polycarbonates are useful as surfactants, electrolytes and binders in batteries, thermoplastic elastomers, pressure sensitive adhesives and as engineering plastics^1,37–40^. To understand the influence of CO_2_ pressure on copolymerization rates, we selected five known high-performance catalysts and investigated the influence of the carbon dioxide pressure applied to each of their polymerization rates. In all cases, the CO_2_ pressure applied during the catalysis influences the copolymerization kinetics and for each catalyst the CO_2_ insertion equilibrium constant is quantified. Combining the different catalyst threshold pressures and insertion equilibria, we reveal a general method to evaluate, optimize and compare new catalysts. For new catalysts it is possible using a single experiment to predict the lowest CO_2_ pressure required to achieve maximum rates and selectivity.

## Results and discussion

### Catalyst selection

**Fig. 2:**
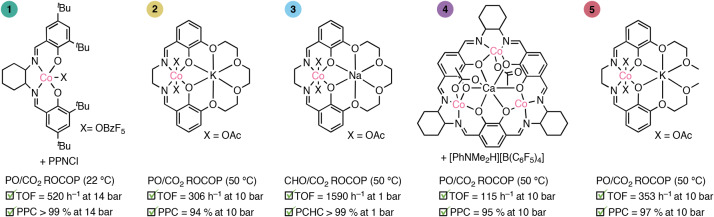
Catalysts studied in this work. The structures and key data for the catalysts **1**^33^, **2**^34^, **3**^34^, **4**^41^ and **5**^13^. The figure includes turnover frequency (TOF) and selectivity for polycarbonate data at the lowest carbon dioxide pressure reported in the original publications. ^*t*^Bu, tertiary butyl (–C(CH_3_)_3_); OBzF_5_, pentafluorobenzoate (–OCOC_6_F_5_); Ph, phenyl (–C_6_H_5_); PPNCl, bis(triphenylphosphine)iminium chloride; OAc, acetate (–OCOCH_3_).

Direct comparisons between catalysts in this field is challenging as rates are reported under individually selected and/or optimized conditions. Analysis of the literature shows many require high CO_2_ pressures and moderate temperatures, challenging the selection of catalysts operating at the low pressures and higher temperatures desirable for scale-up (Supplementary Fig. 1). To select catalysts suitable for in-depth kinetic investigations of CO_2_ pressure dependence, we evaluated the literature for catalysts with precedent for good activity (turnover frequency >100 h^−1^) and selectivity (>90%) at low CO_2_ pressures (≤10 bar) (Supplementary Fig. 1 and Supplementary Table 2). We also selected catalysts that retain their performances at medium to high temperatures (45–100 °C), so as to maximize rates while minimizing polymer viscosity^29^. From a long list of catalysts showing optimum performances at pressures below 30 bar, catalysts **1**–**4** were selected^13,33,34,41^. They fulfil the performance criteria, are synthetically accessible and represent different classes including both mono-metallic or multi-metallic catalysts and catalysts operating with or without cocatalysts (Fig. 2). The rates for each of these catalysts were determined under varied CO_2_ pressures (2–30 bar) and using otherwise identical conditions^33,34,41^. The results of these experiments are compared to the previously reported Co(III)K(I) catalyst **5** for which the CO_2_ insertion equilibrium was proposed^13^ (Fig. 2).

All these leading catalysts are cobalt complexes, as it outperforms other metal choices^21,33,35,42^ (Supplementary Table 2). Since the leading commercial epoxide is PO, PO–CO_2_ ROCOP catalysts were investigated first^9,35^. However, the copolymerization of CHO (CHO–CO_2_ ROCOP) is also a commonly used catalyst benchmark in the field, hence catalyst **3** was investigated for CHO–CO_2_ ROCOP^34^.

### Kinetics

Catalysts **1**–**5** were prepared according to literature procedures^13,33,34,41^, characterized using infrared and ultraviolet-visible light spectroscopy and, where possible, using ^1^H and ^13^C NMR spectroscopy (Supplementary Figs. 2–33).

To investigate whether catalysts **1**–**4** exhibit behaviours consistent with a metal-alkoxide and CO_2_ insertion equilibrium, kinetic analyses were conducted using each catalyst. All reactions were conducted in neat epoxide since these conditions are desirable at scale (no organic solvents). Catalysts should also control the polycarbonate molar mass, dispersity and polymer chain end-groups, specifically maintaining activity when using excess (di)alcohols, controlling for (di)hydroxyl polymer end-groups^43^. All polymerizations were therefore conducted in the presence of *trans*-1,2-cyclohexanediol in neat epoxide, that is [catalyst]:[diol]:[epoxide] = 1:20:4,000 at 50 °C.

**Fig. 3:**
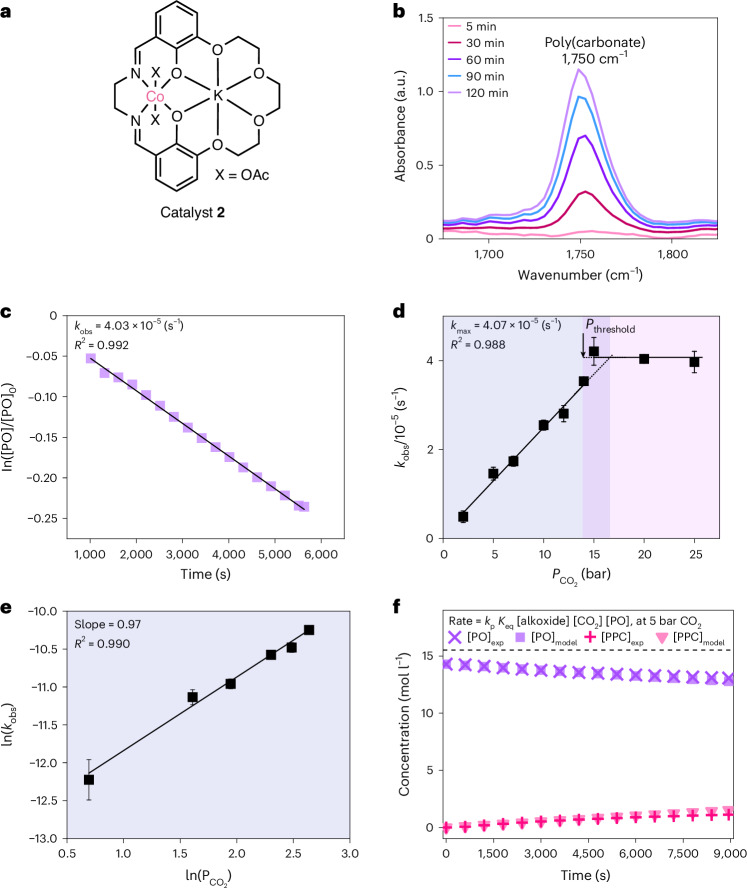
Kinetic analysis conducted using catalyst 2. **a**, Structure of catalyst **2**. **b**, In situ infrared spectrum, showing the peaks corresponding to PPC (1,750 cm^−1^). **c**, Exemplar semi-logarithmic plot of [PO]/[PO]_0_ versus time, where the rate constant, *k*_obs_, is the slope of the graph, using the rate data for the polymerization at 25 bar CO_2_. It has previously been shown that values for such initial rates compare very well with integrated rates determined over wider conversion ranges (20–80%)^51^. **d**, Experimentally determined rates (*k*_obs_) versus *P*(CO_2_). All rate constants were determined as the average of *n* = 2 independent runs, with errors indicated as ± the standard error from the mean, typically falling ±10%. *k*_max_ is the maximum polymerization rate constant, determined as the mean of the *k*_obs_ values in the CO_2_ pressure independent regime. **e**, Plot of ln(*k*_obs_) versus ln(*P*(CO_2_)) showing a gradient of 0.97. All ln(*k*_obs_) values were determined from *k*_obs_ values obtained as the average of *n* = 2 independent runs, with errors indicated as ± the standard error from the mean. **f**, Concentration versus time plot showing the excellent agreement between the experimental data and data generated using the rate law and modelled using COPASI software (Supplementary Fig. 34 and Supplementary Tables 3–6). Source data

Taking catalyst **2** as a representative example of the experiments conducted for each of the catalysts, first, a series of polymerizations were conducted under CO_2_ pressures from 2 bar to 25 bar. At a fixed pressure, polymerizations were monitored using in situ infrared spectroscopy allowing for the calculation of a pseudo first-order rate constant, *k*_obs_, from the semi-logarithmic plot of ln([epoxide]/[epoxide]_0_) versus time (Fig. 3b,c). Experiments were conducted at least in duplicate to enable quantification of errors. Next the *k*_obs_ values were plotted against the applied CO_2_ pressures, showing a linear increase in rate from 2–14 bar (Fig. 3d). The same data plotted as ln(*k*_obs_) versus ln(P(CO_2_)) have a gradient of 0.97, indicating a first-order rate dependence on CO_2_ pressure (Fig. 3e). At pressures above 14 bar CO_2_, the rates were constant and no further increase in *k*_obs_ was observed. Above the threshold pressure (14 bar), *P*_threshold_, there is a zero-order dependence on CO_2_ pressure (Fig. 3d). Polymerization rates were also plotted against [CO_2_], showing the same trends^13,44^ (Supplementary Figs. 43–46).

The experimental kinetic data for all the catalysts were collected and analysed in the same way as for catalyst **2**. All catalysts showed two different regimes of activity versus CO_2_ pressure, and for each catalyst a threshold pressure was identified as the lowest operating pressure for maximum rates (Supplementary Figs. 43–46). These kinetic data indicate that catalysts **1**–**4** all show metal-alkoxide and CO_2_ insertion equilibria (Fig. 1b). These data are interpreted by low pressures resulting in CO_2_ insertion equilibria controlling the effective concentration of the carbonate intermediate, which is the key intermediate in the catalytic rate-determining step. Above the threshold CO_2_ pressure, the equilibrium lies towards the carbonate and maximum rates result^13^.

The CO_2_ insertion equilibrium constant was determined for all five catalysts in the same way. In brief, in the CO_2_ pressure independent regime, the equilibrium lies entirely towards the carbonate intermediate, hence [catalyst]_0_ = [carbonate], assuming the catalyst can only be speciated as an alkoxide or carbonate intermediate (that is, no catalyst decomposition occurs). The carbonate intermediate concentration at a given pressure, [carbonate]_*p*_, was determined from the ratio of the rate constant at that pressure and the maximum rate constant, multiplied by maximum carbonate concentration. Accordingly, the CO_2_ insertion equilibrium constant, *K*_eq_, was determined at each pressure and an average value determined (Supplementary Tables 7–12).

1 $${K}_{{\rm{eq}}}=\frac{[{\rm{carbonate}}]}{[{\rm{alkoxide}}][{{\rm{CO}}}_{2}]}$$

The five catalysts show *K*_eq_ values that vary from 0.25 ± 0.02 M^−1^ for catalyst **4** to 3.10 ± 0.26 M^−1^ for catalyst **3** (Supplementary Tables 7–12). A common rate law was proposed that accounts for the influence of the CO_2_ insertion equilibrium and the concentration of the metal-alkoxide intermediate: this rate law applies at all CO_2_ pressures (equation 2). Next, for each catalyst the experimental conversion versus time data was compared to data modelled using the rate law (Fig. 3f for catalyst **2** and Supplementary Fig. 34). All catalysts showed an excellent agreement between the experimental and kinetic model data over the entire data range. This finding, applicable to all five catalysts, underscores the generality of the rate law and utility of quantifying the CO_2_ insertion equilibria (*K*_eq_).

2 $${\rm{Rate}}={k}_{{\rm{p}}}{K}_{{\rm{eq}}}\left[{\rm{alkoxide}}\right][{{\rm{CO}}}_{2}]\left[{\rm{epoxide}}\right]$$

Variable substrate concentration-rate dependencies, observed with CO_2_ pressure in this work, are also known in other fields, for example enzyme kinetics^45^. The data for catalyst **2** were also fit using a Michaelis–Menten kinetic model. Plotting the initial rate, *v*_i_, against [CO_2_], and fitting with the Michaelis–Menten model, revealed *K*_eq_ = 0.49 M^−1^ and *v*_max_ = 6.6 × 10^−4^ Ms^−1^, which are in good agreement with the values for *K*_eq_ = 0.62 ± 0.09 M^−1^ and *v*_max_ = 5.8 × 10^−4^ Ms^−1^ determined using the rate law presented in this work (Supplementary Fig. 52). The benefit of using our experimental and kinetic methods are that they directly determine the carbon dioxide insertion equilibrium constant *K*_eq_ and the threshold CO_2_ pressure *P*_threshold_, the latter is absent from saturation kinetic models.

### Generality of metal alkoxide and CO_2_ insertion equilibrium

**Fig. 4:**
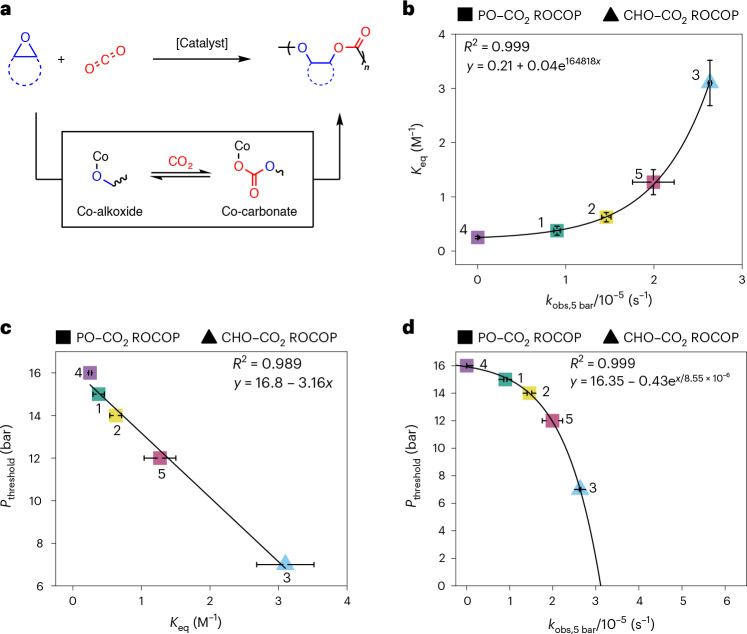
Relating the CO_2_ insertion equilibrium constant, *K*_eq_, to polymerization rates. **a**, General reaction scheme for epoxide–CO_2_ ROCOP showing the proposed CO_2_ insertion equilibrium between a Co-alkoxide and a Co-carbonate intermediate. **b**, Plot showing all catalysts and values for *K*_eq_ versus *k*_obs_ at 5 bar CO_2_ pressure and 50 °C. All *k*_obs_ values were determined as the average of *n* = 2 independent runs, with errors indicated as ± the standard error from the mean, typically falling ±10%. Values for *K*_eq_ and corresponding errors were calculated as indicated in Supplementary Tables 7–12. **c**, Plot showing data for all catalysts with *P*_threshold_ versus *K*_eq_. Values for *K*_eq_ and corresponding errors were calculated as indicated in Supplementary Tables 7–12. **d**, Plot showing data for all catalysts with *P*_threshold_ versus *k*_obs_, at 5 bar CO_2_ pressure and 50 °C. All *k*_obs_ values were determined as the average of *n* = 2 independent runs, with errors indicated as ± the standard error from the mean, typically falling ±10%. In all plots, squares represent PO–CO_2_ ROCOP and triangles represent CHO–CO_2_ ROCOP. The symbol colours correspond to the catalysts colour with their associated numbering as defined in Fig. 2. Data for catalyst 5 were taken from ref. ^13^. Source data

According to the common rate law, the CO_2_ insertion equilibrium constant directly correlates with the polymerization rate. Therefore, we plotted the CO_2_ insertion equilibrium constant, *K*_eq_, for each of catalysts **1**–**5**, against their rates, measured at 5 bar CO_2_ and 50 °C (*k*_obs,5 bar_). The plot reveals a clear exponential correlation between carbon dioxide insertion equilibrium constant and rate (Fig. 4b and Supplementary Fig. 53). The highest performing catalyst (**3**) exhibits the highest CO_2_ insertion equilibrium constant, 3.10 ± 0.26 M^−1^. Conversely, the lowest performing catalyst (**4**) has the lowest CO_2_ insertion equilibrium constant, 0.25 ± 0.02 M^−1^ (Fig. 4b–d).

These activity correlations underline the importance of CO_2_ insertion equilibria across the different catalysts and provide a new way to compare and design better catalysts. To interpret the data, a mechanism whereby the rate-determining step involves catalyst carbonate attack on the epoxide is invoked (Fig. 1b). The concentration of this key catalyst species (metal carbonate) is controlled by the CO_2_ insertion equilibrium. Thus, catalysts with favourable CO_2_ insertion equilibria, for example catalyst **3**, show greater catalytic performance. The slower and less selective catalysts, for example catalysts **1** or **4**, show low CO_2_ insertion equilibrium constants, consistent with higher concentrations of residual alkoxide intermediate.

The threshold CO_2_ pressure, *P*_threshold_, values describe the minimum CO_2_ pressure to achieve the maximum rate. These values are likely to be very important to any process to make polymers^1,4–6^. The values vary considerably for catalysts **1**–**5** from 7–16 bar CO_2_. Plotting, *P*_threshold_ against *K*_eq_, for catalysts **1**–**5** reveals a linear correlation (Fig. 4c). The better performing catalysts have higher equilibrium constants and lower *P*_threshold_: that is, they reach maximum performance at lower CO_2_ pressures. *P*_threshold_ also exponentially correlates to the measured *k*_obs,5 bar_ (Fig. 4d). This kinetic treatment directly links two measurable variables: the rate constant, *k*_obs,5 bar_ and *P*_threshold_, a parameter central to low energy process operation. Such a correlation is particularly important, since it identifies how to maximize performances while minimizing the operating pressure with its associated economic and environmental costs (Supplementary Table 1).

### Prediction of equilibrium parameters

**Fig. 5:**
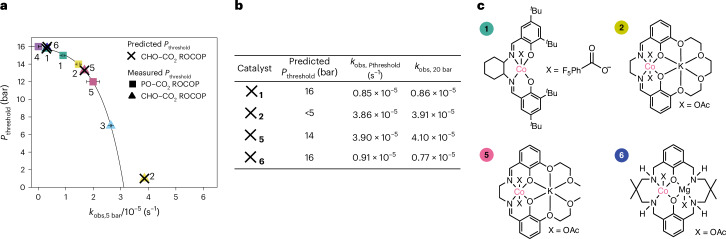
Prediction of threshold pressures for maximum catalytic performance. **a**, Plot of threshold pressure for maximum catalytic performance (*P*_threshold_) versus polymerization rate constant, *k*_obs_, at 5 bar CO_2_ pressure and 50 °C. Squares represent *k*_obs_ for the PO–CO_2_ ROCOP, and triangles for CHO–CO_2_ ROCOP. *k*_obs_ values were determined as the average of *n* = 2 independent runs, with errors indicated as ± the standard error from the mean, typically falling ±10%. Data for catalyst 5 were taken from ref. ^13^. Crosses represent measured values of *k*_obs,5 bar_ and predicted *P*_threshold_ values for catalysts **1**, **2**, **5** and **6** for the CHO–CO_2_ ROCOP (Supplementary Table 13). **b**, Table showing the predicted *P*_threshold_ and the measured catalytic performance at *P*_threshold_ and at a pressure above it (20 bar) for catalysts **1**, **2**, **5** and **6**. The rate data, *k*_obs_, were obtained at 50 °C, using catalyst (0.025 mol%, 2.5 mM), CHO (6 ml, 9.9 M), *trans*-1,2-cyclohexanediol (0.5 mol%, 49 mM). **c**, Structures of the catalysts **1**, **2**, **5** and **6** for which *P*_threshold_ was successfully predicted. The symbol colours in the graphs correspond to the catalysts colour with their associated numbering as defined in Fig. 2. Source data

There are clear correlations between rate, equilibrium and threshold pressure that apply to all five catalytic systems, which span a wide range of different catalyst classes (Fig. 2). One consequence is that these correlations could be generalizable to other epoxide–CO_2_ copolymerizations, conducted isothermally, with experiments in this work all being conducted at 50 °C. It may be that the carbon dioxide insertion *K*_eq_ and *P*_threshold_ are predictable for any catalyst or monomer using a single measurement of rate coefficient, *k*_obs_, which is set in this work at 5 bar. To experimentally test this hypothesis, a previously reported heterodinuclear Co(II)Mg(II) catalyst (**6**), not included in the original catalyst selection, was synthesized and examined for CHO–CO_2_ ROCOP^46^ (Supplementary Fig. 54 and Supplementary Table 13). Catalyst **6** was selected as it showed very high performances in the copolymerization catalysis, including operating at low loadings and temperatures. In addition, catalysts **1**, **2** and **5**, previously investigated for PO–CO_2_ ROCOP, were each examined for a second monomer combination: CHO–CO_2_ ROCOP. First, each catalyst was monitored for CHO–CO_2_ ROCOP, at 5 bar CO_2_ pressure, to obtain the rate coefficient *k*_obs,5 bar_. Using *k*_obs,5 bar_, the CO_2_ insertion equilibrium constant, *K*_eq_, and threshold pressure, *P*_threshold_, were predicted using the exponential relationships that were previously uncovered (Figs. 4 and 5 and Supplementary Table 13). The predicted equilibrium constants for catalysts **1**, **5** and **6** are comparable, with *K*_eq_ = 0.27 M^−1^ (**1**), 0.85 M^−1^ (**5**) and 0.28 M^−1^ (**6**) (Supplementary Table 13). They also show similar, high, values for the predicted *P*_threshold_ = 16 bar (**1** and **6**) and 14 bar (**5**). In contrast, catalyst **2** has a substantially higher predicted CO_2_ insertion equilibrium constant, *K*_eq_ = 23.4 M^−1^ and a notably lower predicted *P*_threshold_ (Fig. 5). To further investigate the general predictability of *K*_eq_, a previously reported organoborane catalyst was tested for CHO–CO_2_ ROCOP^47^ (Supplementary Fig. 54 and Supplementary Table 13). However, the observed activities were too low under these conditions to warrant further equilibrium or rate analysis.

Following the predictions of *P*_threshold_ and *K*_eq_, the accuracy of the predicted values for catalysts **1**, **2**, **5** and **6** was tested experimentally. For catalyst **5**, CHO–CO_2_ ROCOP was investigated at pressures from 5 bar to 25 bar, with the *P*_threshold_ being 14 bar and matching the predicted value (Supplementary Fig. 55). For catalyst **1** and **6**, the value of the predicted pressure threshold is tested by conducting CHO–CO_2_ ROCOP at the predicted *P*_threshold_ (16 bar CO_2_, Fig. 5), and at a higher pressure than the threshold (20 bar CO_2_). If the predicted *P*_threshold_ is accurate, the catalytic performance should remain unchanged and indeed, the *k*_obs_ is equivalent at each of those pressures (*k*_obs,16 bar_ = 0.85 × 10^−5^ s^−1^, *k*_obs,20 bar_ = 0.86 × 10^−5^ s^−1^ for catalyst **1** and *k*_obs,16 bar_ = 0.91 × 10^−5^ s^−1^, *k*_obs,20 bar_ = 0.77 × 10^−5^ s^−1^ for catalyst **6**). Catalyst **5** was applied at pressures of 14 (predicted threshold pressure) and 20 bar; it showed very similar rates at both pressures (*k*_obs,14 bar_ = 3.90 × 10^−5^ s^−1^, *k*_obs,20 bar_ = 4.10 × 10^−5^ s^−1^) once again validating the successful identification of the threshold pressure.

Since catalyst **2** performs far better at 5 bar than the other two catalysts, its *P*_threshold_ was predicted to be below 5 bar. This result implies that catalyst **2** has already achieved its maximum rates at 5 bar. Hence, catalyst **2** showed the same catalytic performance at both 5 bar and 20 bar (*k*_obs,5 bar_ = 3.86 × 10^−5^ s^−1^, *k*_obs,20 bar_ = 3.91 × 10^−5^ s^−1^), strongly suggesting that its *P*_threshold_ is <5 bar (Fig. 5 and Supplementary Table 13). These experiments are surprising since the data generated using PO–CO_2_ ROCOP can be used to successfully predict *P*_threshold_ for CHO–CO_2_ ROCOP and to accelerate identification of the optimum operating conditions, that is, minimum pressure for maximum rate. The results obtained using catalysts **1**, **2**, **5** and **6** for the CHO–CO_2_ ROCOP indicate that the kinetic methods may be further generalizable to other monomers. It is particularly helpful to use a single kinetic evaluation (*k*_obs,5 bar_) to identify and predict the optimum operating conditions for that catalyst.

**Fig. 6:**
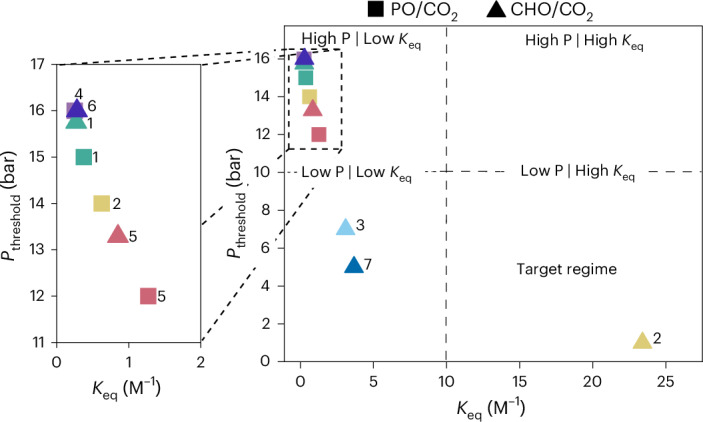
Relating the value of CO_2_ insertion equilibrium constants to threshold pressures for maximum catalytic performance. Plot of the combined data for all catalysts showing the carbon dioxide threshold pressure values, *P*_threshold_, and CO_2_ insertion equilibrium constants, *K*_eq_, determined in this investigation (with colours identifying different catalysts and square markers referring to PO–CO_2_ ROCOP, triangles to CHO–CO_2_ ROCOP). The plot shows that only catalyst **2** applied for CHO–CO_2_ ROCOP achieves the target low pressure (<10 bar) and high insertion equilibrium (>10 M^−1^) performance. Source data

The general applicability of *K*_eq_ as a predictor of catalytic performance is further illustrated by three other known catalysts for which pressure-dependent kinetics were reported^26,27,48^. An antimony catalyst (with bis(triphenylphosphine)iminium chloride co-catalyst) showed an equilibrium constant of <1 bar^−1^ in CHO–CO_2_ ROCOP, resulting in a *P*_threshold_ > 20 bar (Supplementary Fig. 56). A similar *P*_threshold_ above 20 bar was reported for a di-Zn(II) β-diiminate catalyst. By contrast, a zinc β-diiminate catalyst **7** showed a threshold pressure <5 bar CO_2_ pressure^26^. In the original reports no equilibrium constants were determined; however, we estimated *K*_eq_ <1 M^−1^ and *K*_eq_ ~ 3.67 M^−1^ for the zinc catalysts, respectively (Fig. 6, catalyst **7**).

The kinetic prediction method indicates that only catalysts with *k*_obs,5 bar_ > 2.5 × 10^−5^ s^−1^ (*K*_eq_ > 10 M^−1^), are expected to show CO_2_ pressure independent performances below 10 bar. The PO–CO_2_ ROCOP catalysts examined all show *K*_eq_ < 5 M^−1^, and at 5 bar all show performances that depend on carbon dioxide pressure (squares, Fig. 6). Even in the broader literature there are not yet any PO–CO_2_ ROCOP catalysts known or reported to enter the target regime: that is, showing high rates and selectivity at <10 bar pressure. The methods presented here should help accelerate new catalyst testing and identify catalyst structure–performance relationships, particularly focused on how catalyst structure drives carbon dioxide insertion chemistry. In contrast, for CHO–CO_2_ ROCOP, catalyst **2** already shows a *K*_eq_ > 10 M^−1^ and achieves excellent performances at <5 bar CO_2_ pressure (Fig. 6). The literature also reveals other CHO–CO_2_ ROCOP catalysts reported to exhibit high rates at low CO_2_ pressures; these catalysts are recommended for evaluation using the methods reported here to confirm whether pressure independent performances are achieved at <10 bar. Where such criteria are achieved, the catalysts may even function using more dilute CO_2_ sources (that is, <1 bar pressure): a regime that is very rarely explored in this field of catalysis^46,49^.

Both catalyst structure and monomer choice influence the CO_2_ insertion chemistry, highlighting the value in using the CO_2_ insertion parameters, *K*_eq_ and *P*_threshold_, as metrics for catalyst comparison. One benefit of these metrics is that they inform directly on the process operating conditions with respect to catalytic performance. Comparing catalysts using these parameters may be more informative than the conventional use of activity (that is, turnover frequency) as figure of merit. It is well known that such activity measurements are both monomer and condition dependent; in contrast, *K*_eq_ allows for comparisons over a range of CO_2_ pressures and different epoxides. The ability to rapidly predict the carbon dioxide insertion *K*_eq_ or *P*_threshold_ values drastically reduce the experimental work needed to compare new catalysts and to identify the lowest pressure operating conditions for them.

The correlations and the practical experimental protocols should be especially useful in selecting catalysts for larger-scale use, and in identification of their optimum operating conditions^50^. One question raised by this work is the extent to which similar CO_2_ insertion equilibria, and threshold pressures, may apply to other CO_2_ utilization catalyses? The use of the methods outlined in this report is recommended to explore CO_2_ insertion equilibria in other catalytic cycles. The methods may be particularly useful in identifying operable, fast and selective catalysts that are needed to implement large-scale, energy efficient CO_2_ uses.

## Conclusions

The influence of CO_2_ pressure on its insertion reactions into metal-alkoxide bonds was investigated using structurally diverse, high-performance epoxide and carbon dioxide ROCOP catalysts. Systematic experiments, conducted using epoxide and CO_2_ at pressures from 2 bar to 30 bar, revealed that all five catalysts show pressure-dependent CO_2_ insertion equilibria. A rate law, applicable under all pressures, was presented and interpreted by a mechanism accounting for differing extents of carbon dioxide insertion. Two reaction parameters are identified and methods to quantify them are presented: the CO_2_ insertion equilibrium constant (*K*_eq_) and the threshold carbon dioxide pressure above which rates are maximized (*P*_threshold_). Direct correlations were uncovered between catalytic rate and the CO_2_ insertion equilibrium constant and threshold pressure. The data, and parameter inter-relationships, were used to successfully predict the performances for known catalysts using new monomers (CHO–CO_2_). The use of one measurement, rather than full kinetic analyses, substantially reduces the experimental work required to identify the lowest pressure process operating conditions and provides a reliable set of metrics by which to compare catalysts. The findings are particularly important for future catalyst design, specifically the under-recognized importance of design for CO_2_ insertion, and in enabling scale-up of catalysts resulting in the lowest energy demand. Given the importance of CO_2_ insertion equilibria in influencing this field of carbon dioxide copolymerization catalysis, it seems possible, if not likely, that other catalysts may also show related CO_2_ pressure dependence. Identifying and quantifying the lowest energy conditions for such CO_2_ insertions is really important to accelerate delivery of large-scale CO_2_ utilization processes and products.

## Methods

### CHO–CO_2_ and PO–CO_2_ ROCOP procedures and experimental apparatus

The same procedures were followed when conducting the CHO–CO_2_ and PO–CO_2_ ROCOP. A representative method for the CHO–CO_2_ ROCOP is detailed here.

The catalyst (0.015 mmol), *trans*-1,2-cyclohexanediol (35 mg, 0.3 mmol) and mesitylene (21 µL, 0.15 mmol, internal standard for monomer conversion quantification) were dissolved in CHO (6 ml, 59 mmol), under a nitrogen atmosphere. The solution was injected into a 100-ml Parr reactor fitted with an in situ attenuated total reflectance-infrared spectroscopy probe, under a flow of CO_2_. The reactor was pressurized to the desired CO_2_ pressure and heated to 50 °C. Mass flow controllers were used to ensure fixed and constant CO_2_ pressure throughout the reaction. The reaction was monitored by changes to infrared peaks at 1,750 cm^−1^ (polycarbonate) and 1,820 cm^−1^ (cyclic carbonate). The polymerization was stirred continually at the desired temperature and pressure until >30% polycarbonate formation. The reaction was then cooled and quenched by exposure to air and addition of benzoic acid (2 mg, 0.016 mmol). The epoxide conversion data was externally calibrated by ^1^H NMR spectroscopy using a crude reaction aliquot, using the added mesitylene as the internal standard.

The reaction rate coefficient, *k*_obs_ was determined as the gradient of plots of ln([epoxide]/[epoxide]_0_) versus time (Supplementary Figs. 39–42), typical epoxide conversions are 5–20%. For each catalyst, epoxide–CO_2_ polymerizations were conducted under the same reaction conditions ([catalyst]:[diol]:[epoxide] = 1:20:4,000). For catalysts **1** and **4**, [catalyst]:[cocatalyst] = 1:1. Polymerizations were conducted at 50 °C at a fixed CO_2_ pressure, with values systematically increased from 2 bar to 35 bar CO_2_, using the reaction set up outlined above. All reactions were run in duplicate, with generally ±10% error. Using previously reported data for the solubility of CO_2_ in PO and CHO^13,44^, polymerization rates were plotted against both [CO_2_] and CO_2_ pressure.

### Catalyst syntheses

The catalysts **1**–**5** were synthesized according to literature reports. Detailed synthetic procedures for each of catalysts **1**^33^, **2**^34^, **3**^34^, **4**^41^ and **5**^13^ are provided in the Supplementary Information.

## Online content

Any methods, additional references, Nature Portfolio reporting summaries, source data, extended data, supplementary information, acknowledgements, peer review information; details of author contributions and competing interests; and statements of data and code availability are available at 10.1038/s41557-026-02098-6.

## Supplementary information

Supplementary Information Supplementary Methods, Materials and Experimental Details, including Figs. 1–56, Discussion and Tables 1–14.

## Source data

Source Data Fig. 3 Supplementary numerical source data for graphs in Fig. 3.

Source Data Fig. 4 Supplementary numerical source data for graphs in Fig. 4.

Source Data Fig. 5 Supplementary numerical source data for graphs in Fig. 5.

Source Data Fig. 6 Supplementary numerical source data for graphs in Fig. 6.

## Data Availability

Data files for the information contained in the paper and supporting information are open access available via the Oxford University Research Archive at https://ora.ox.ac.uk/objects/uuid:94ebb3ae-29ab-4daa-b186-c9f28600ce48 and 10.5287/ora-prkzkoyj9. Source data are provided with this paper.
