# Supplementary material for: Understanding low-pressure CO2 insertion chemistry in epoxide–CO2 copolymerization catalysis
Source: Nat Chem. 2026 Apr 30;18(5):931–8. doi: 10.1038/s41557-026-02098-6 (PMC13149301; doi:10.1038/s41557-026-02098-6)
Supplement: Supplementary file 1 — Supplementary Methods, Materials and Experimental Details, including Figs. 1–56, Discussion and Tables 1–14. [file 41557_2026_2098_MOESM1_ESM.pdf]

nature chemistry

Supplementary information

https://doi.org/10.1038/s41557-026-02098-6

Understanding low-pressure CO_2 insertion chemistry in epoxide–CO_2 copolymerization catalysis

In the format provided by the authors and unedited

TABLE OF CONTENTS

DATA AVAILABILITY 6

METHODS AND MATERIALS 6

Materials 6

Analytical Methods 6

Calculations and Data for CO_2 Compression Costs 6

Supplementary Table 1 Data for CO_2 Compressor Costs and Electricity Requirements 7

Ligand and Complex Syntheses 8

Synthesis of Catalyst 1[1] 8

Syntheses of Catalysts 2 and 3[2] 8

Synthesis of Catalyst 4[5] 9

Synthesis of Catalyst 5[3] 10

ADDITIONAL INFORMATION 12

Supplementary Figure 1 Overview of reported catalysts for PO–CO_2 ROCOP, according to their optimum operating conditions (pressure and temperature). 12

Supplementary Table 2 Leading Catalysts for PO–CO_2 ROCOP 13

Supplementary Figure 2 ^{1}H NMR Spectrum of Catalyst 1. 16

Supplementary Figure 3 ^{13}C^{13} NMR Spectrum of Catalyst 1 17

Supplementary Figure 4 2D ^{1}H -^{13}C HSQC Spectrum of Catalyst 1. 17

Supplementary Figure 5 2D ^{1}H -^{13}C HMBC Spectrum of Catalyst 1. 18

Supplementary Figure 6 2D ^{1}H -^{1}H COSY Spectrum of Catalyst 1. 18

Supplementary Figure 7 IR Spectrum of Catalyst 1. 19

Supplementary Figure 8 UV/Vis Spectrum of Catalyst 1 (0.125 mM in MeCN). 19

Supplementary Figure 9 ^{1}H NMR Spectrum of Catalyst 2. 20

Supplementary Figure 10 ^{13}C{^{1}H} NMR Spectrum of Catalyst 2. 20

Supplementary Figure 11 2D ^{1}H -^{13}C HSQC Spectrum of Catalyst 2. 21

Supplementary Figure 12 2D ^{1}H -^{13}C HMBC Spectrum of Catalyst 2. 21

Supplementary Figure 13 2D ^{1}H -^{1}H COSY Spectrum of Catalyst 2. 22

Supplementary Figure 14 IR Spectrum of Catalyst 2. **22**

Supplementary Figure 15 UV/Vis Spectrum of Catalyst 2 (0.125 mM in MeCN). **23**

Supplementary Figure 16 2D ^{1}H NMR Spectrum of Catalyst 3. **23**

Supplementary Figure 17 ^{13}C{^{1}H} NMR Spectrum of Catalyst 3. **24**

Supplementary Figure 18 2D ^{1}H -^{13}C HSQC Spectrum of Catalyst 3. **24**

Supplementary Figure 19 2D ^{1}H -^{13}C HMBC Spectrum of Catalyst 3. **25**

Supplementary Figure 20 2D ^{1}H -^{1}H COSY Spectrum of Catalyst 3. **25**

Supplementary Figure 21 IR Spectrum of Catalyst 3. **26**

Supplementary Figure 22 UV/Vis Spectrum of Catalyst 3 (0.125 mM in MeCN). **26**

Supplementary Figure 23 ^{1}H NMR Spectrum of 2,3-dimethoxybenzene-1,4-dicarbaldehyde. **27**

Supplementary Figure 24 ^{1}H NMR Spectrum of crude 2,3-dihydroxybenzene-1,4-dicarbaldehyde. **27**

Supplementary Figure 25 IR Spectrum of Catalyst 4. **28**

Supplementary Figure 26 UV/Vis Spectrum of Catalyst 4 (0.125 mM in MeCN). **28**

Supplementary Figure 27 ^{1}H NMR Spectrum of Catalyst 5. **29**

Supplementary Figure 28 ^{13}C{^{1}H} Spectrum of Catalyst 5. **29**

Supplementary Figure 29 2D ^{1}H -^{13}C HSQC Spectrum of Catalyst 5. **30**

Supplementary Figure 30 2D ^{1}H -^{13}C HMBC Spectrum of Catalyst 5. **30**

Supplementary Figure 31 2D ^{1}H -^{1}H COSY Spectrum of Catalyst 5. **31**

Supplementary Figure 32 IR Spectrum of Catalyst 5. **31**

Supplementary Figure 33 UV/Vis Spectrum of Catalyst 5 (0.125 mM in MeCN). **32**

POLYMERIZATION AND KINETIC DATA 33

Modelling of Rate Laws for Epoxide–CO_2 ROCOP with Catalysts 1-4 (Using COPASI) **33**

Supplementary Figure 34 Modelling for epoxide–CO_2 ROCOP with catalysts 1-4 (using COPASI). **34**

Supplementary Figure 35 Representative GPC trace for PO–CO_2 ROCOP with catalyst 1 + PPNCl at 50 °C, 5 bar CO_2, 1:20:4000:1 catalyst 1:diol:PO:PPNCl. **35**

Supplementary Figure 36 Representative GPC trace for PO–CO_2 ROCOP with catalyst 2 at 50 °C, 5 bar CO_2, 1:20:4000 catalyst 2:diol:PO. 35

Supplementary Figure 37 Representative GPC trace for CHO–CO_2 ROCOP with catalyst 3 at 50 °C, 5 bar CO_2, 1:20:4000 catalyst 3:diol:PO. 36

Supplementary Figure 38 Representative GPC trace for PO–CO_2 ROCOP with catalyst 4 + [$PhNMe_2H$][$B(C_6F_5)_4$] at 50 °C, 5 bar CO_2, 1:20:4000:1 Catalyst 4:diol:PO:[$PhNMe_2H$][$B(C_6F_5)_4$]. 36

Supplementary Figure 39 Exemplar semilogarithmic plot of ln([PO]/[PO]$_0$) *vs* time for catalyst 1 and a PPNCl co-catalyst. 37

Supplementary Figure 40 Exemplar semilogarithmic plot of ln([PO]/[PO]$_0$) vs time for catalyst 2. 37

Supplementary Figure 41 Exemplar semilogarithmic plot of ln([CHO]/[CHO]$_0$) vs time for catalyst 3. 38

Supplementary Figure 42 Exemplar semilogarithmic plot of ln([PO]/[PO]$_0$) vs time with catalyst 4 and a [$PhNMe_2H$][$B(C_6F_5)_4$] co-catalyst. 38

Supplementary Table 3 Polymerization data for PO–CO_2 ROCOP with catalyst 1 and a PPNCl co-catalyst. 39

Supplementary Table 4 Polymerization data for PO–CO_2 ROCOP with catalyst 2. 40

Supplementary Table 5 Polymerization data for CHO–CO_2 ROCOP with catalyst 3. 40

Supplementary Table 6 Polymerization data for PO–CO_2 ROCOP with catalyst 4 with a [$PhNMe_2H$][$B(C_6F_5)_4$] co-catalyst. 41

Supplementary Figure 43 Plots of k_{obs} *vs*. [CO_2] and k_{obs} *vs*. P(CO_2) for PO–CO_2 ROCOP with catalyst 1 with PPNCl co-catalyst. k_{obs} values were determined as the average of n=2 independent runs, with errors indicated as ± the standard error from the mean, typically falling ± 10%. 42

Supplementary Figure 44 Plots of k_{obs} *vs*. [CO_2] for PO–CO_2 ROCOP with catalyst 2. k_{obs} values were determined as the average of n=2 independent runs, with errors indicated as ± the standard error from the mean, typically falling ± 10%. 42

Supplementary Figure 45 Plots of k_{obs} *vs*. [CO_2] and k_{obs} *vs*. P(CO_2) for CHO–CO_2 ROCOP with catalyst 3. k_{obs} values were determined as the average of n=2 independent runs, with errors indicated as ± the standard error from the mean, typically falling ± 10%. 43

Supplementary Figure 46 Plots of k_{obs} *vs*. [CO_2] and k_{obs} *vs*. P(CO_2) for PO–CO_2 ROCOP with catalyst 4 with a [$PhNMe_2H$][$B(C_6F_5)_4$] co-catalyst. k_{obs} values were determined as the average of n=2 independent runs, with errors indicated as ± the standard error from the mean, typically falling ± 10%. 43

Supplementary Figure 47 Plots of ln(k_{obs}) *vs*. ln([CO_2]) and ln(k_{obs}) *vs*. ln(P(CO_2)) for PO–CO_2 ROCOP with catalyst 1 and a PPNCl co-catalyst. All ln(k_{obs}) values were determined

from k_{obs} values obtained as the average of n=2 independent runs, with errors indicated as ± the standard error from the mean. 44

Supplementary Figure 48 Plot of ln(k_{obs}) *vs*. ln([CO_2]) for PO–CO_2 ROCOP with catalyst 2. All ln(k_{obs}) values were determined from k_{obs} values obtained as the average of n=2 independent runs, with errors indicated as ± the standard error from the mean. 44

Supplementary Figure 49 Plots of ln(k_{obs}) *vs*. ln([CO_2]) and ln(k_{obs}) *vs*. ln(P(CO_2)) for CHO–CO_2 ROCOP with catalyst 3. All ln(k_{obs}) values were determined from k_{obs} values obtained as the average of n=2 independent runs, with errors indicated as ± the standard error from the mean. 45

Supplementary Figure 50 Plots of ln(k_{obs}) *vs*. ln([CO_2]) and ln(k_{obs}) *vs*. ln(P(CO_2)) for PO–CO_2 ROCOP with catalyst 4 and a [$PhNMe_2H$][$B(C_6F_5)_4$] co-catalyst. All ln(k_{obs}) values were determined from k_{obs} values obtained as the average of n=2 independent runs, with errors indicated as ± the standard error from the mean. 45

CALCULATING THE CO_2 INSERTION EQUILIBRIUM CONSTANT, K_{EQ} 46

Supplementary Table 7 Calculation of K_{eq} for PO–CO_2 ROCOP with catalyst 1 and a PPNCl co-catalyst. 47

Supplementary Table 8 Calculation of K_{eq} for PO–CO_2 ROCOP with catalyst 2. 47

Supplementary Table 9 Calculation of K_{eq} for CHO–CO_2 ROCOP with catalyst 3. 48

Supplementary Table 10 Calculation of K_{eq} for PO–CO_2 ROCOP with catalyst 4 and a [$PhNMe_2H$][$B(C_6F_5)_4$] co-catalyst. 48

Supplementary Table 11 Calculation of ΔK_{eq} for CHO–CO_2 ROCOP with catalyst 3. 49

Supplementary Table 12 Comparison of the equilibrium constants reported in this work, in units of concentration and in units of pressure. 49

Supplementary Figure 51 a) Plot of the IR absorbance peak area at 2336 cm^{-1} obtained after CO_2 saturation of CO_2 in CHO at pressures of 4 bar, 5 bar, 10 bar, 15 bar and 20 bar CO_2 *vs*. CO_2 pressure. b) Integrated mass flow *vs*. time when pressurizing neat CHO to 20 bar CO_2. c) Plot of the IR absorbance peak area at 2336 cm^{-1} obtained after CO_2 saturation in 1:4000 catalyst 5:CHO, at pressures of 4 bar, 5 bar, 10 bar, 15 bar and 20 bar CO_2 *vs*. CO_2 pressure. d) Integrated mass flow *vs*. time when pressurising a solution of 1:4000 catalyst 5:CHO to 20 bar CO_2. 50

Michaelis Menten Kinetic Model for Catalyst 2 51

Supplementary Figure 52 Non-linear Fitting of Polymerization Rate vs [CO_2] to Obtain K_{eq}. 51

Supplementary Figure 53 Plot of ln(K_{eq}) vs. $k_{obs,5bar}$. All ln(k_{obs}) values were determined from k_{obs} values obtained as the average of n=2 independent runs, with errors indicated as ± the standard error from the mean. Values for K_{eq} and corresponding errors were calculated as indicated in Supplementary Tables 7-11, and values for ln(K_{eq}) were obtained from these values, with errors propagated accordingly. 52

PREDICTION OF EQUILIBRIUM PARAMETERS FOR FURTHER CATALYSTS 53

Supplementary Figure 54 Structures of Catalysts 6 and 8, synthesized according to literature procedures. 53

Supplementary Table 13 Polymerization Rate Data for Prediction of $P_{threshold}$ and K_{eq} using Catalysts 1, 2, 5, 6 and 8 for CHO–CO_2 ROCOP. 53

Supplementary Figure 55 k_{obs} *vs*. P(CO_2) for CHO–CO_2 ROCOP with catalyst 5, identifying $P_{threshold}$ = 14 bar CO_2. 54

Supplementary Figure 56 Three additional catalysts, reported in the literature, for which experimental evidence for a CO_2 insertion equilibrium have been reported. 55

HIGHER PRESSURE INVESTIGATION OF PO–CO_2 ROCOP USING CATALYST 5 56

Supplementary Table 14 Polymerization Rate Data for PO–CO_2 ROCOP using Catalyst 5, for pressures above and including the identified $P_{threshold}$ value. 56

REFERENCES 57

Data Availability

Data files for the information contained in the manuscript and supporting information are open access via https://ora.ox.ac.uk/objects/uuid:94ebb3ae-29ab-4daa-b186-c9f28600ce48 with the DOI: 10.5287/ora-prkzkoyj9 (resolving to https://dx.doi.org/10.5287/ora-prkzkoyj9).

Methods and Materials

Materials

All reagents and solvents were obtained from commercial suppliers and used as received, unless otherwise stated. Anhydrous solvents were obtained from a solvent purification system (SPS), degassed using three freeze-pump-thaw cycles and stored over 3 Å molecular sieves, under a N_2 atmosphere. Anhydrous DMSO was stored over 3 Å molecular sieves, under a N_2 atmosphere for a week before being used. The (*R,R*)-(salcy)Co(II) complex for synthesis of catalyst **1** was sourced from Sigma Aldrich and dried under high vacuum prior to use.[1] The proligands for syntheses of catalysts **2**, **3** and **5** were sourced from Manchester Organics Ltd; the proligand for catalyst **2** and **3** was dried under high vacuum prior to use and the proligand for catalyst **5** was used as received.[2,3] PO and CHO were dried over two nights, by stirring over calcium hydride, and subsequently purified by fractional distillation, then by degassing with N_2, and stored under a N_2 atmosphere. Research-grade CO_2 for the copolymerizations (BOC, CP grade, 99.9995 %) was dried, passing through two drying columns (VICI Metronics Carbon Dioxide Purifier) in series, at 50 bar pressure.

Analytical Methods

The ^{1}H, ^{13}C {^{1}H}, COSY, HSQC and HMBC NMR spectra for the catalysts were measured using a Bruker Avance III spectrometer (^{1}H 500 MHz, ^{13}C 126 MHz) equipped with a 11.75 T magnet, at 298 K. ^{1}H NMR spectra for monitoring reaction progress (and synthesis of intermediate species) was performed using a 400 MHz Bruker Avance III HD Nanobay spectrometer equipped with a 9.4 T magnet, at 298 K.

UV-Vis spectra were recorded on an Ocean optics FLAME-S-UV-VIS spectrometer, connected to an Ocean optics DH-2000-BAL UV-VIS-NIR light source, using a 1 cm quartz cuvette, at room temperature. Measurements were taken inside a nitrogen filled glovebox, using an ocean optics cuvette holder.

FT-IR spectra were conducted with a Shimadzu IRSpirit spectrometer (inside a N_2 glove box), using a single reflection ATR accessory.

Gel permeation chromatography (GPC) was performed on a Shimadzu LC-20AD instrument, with two mixed bed PSS SDV linear S columns, in series, at 40 °C. THF was used as the eluent, with a flow rate of 1 mL/ min. Molar mass calibration was conducted with a narrow molar mass polystyrene standards.

Calculations and Data for CO_2 Compression Costs

The energy requirements and capital costs for the pressurization of CO_2 were estimated using Aspen Plus V14. An input CO_2 flow (2750 kg/h, 1 bar, 298 K) was fed into a compressor set to either 5 bar, 10 bar, 20 bar or 50 bar. The input CO_2 flow of 2750 kg/h was selected based on the input CO_2 flow rate for a representative 50,000 tonne/year poly(propylene carbonate) production plant for which a detailed life cycle assessment was reported.[4] In these calculations a starting CO_2 pressure of 1 bar was assumed. The CO_2 compression energy requirement and capital equipment cost for the compressor was estimated using the Aspen Process Economic Analyzer. The global warming potential of the required electricity was calculated using the 'market for medium voltage-electricity, GB' inventory from the ecoinvent v3.9.1 database and the IPCC 2021 GWP100 method, with Brightway v2.8 software.

Supplementary Table 1 Data for CO_2 Compressor Costs and Electricity Requirements

Calculations were made using Aspen Plus V14.

Pressure	Electricity (kWh)	Electricity per kg CO_2 (kWh/kg$_{CO2}$)	GWP (kg CO_2 eq/kg$_{CO2}$)*	Output Temperature (°C)	Installed Estimated Compressor Cost ($)
5	114.731844	0.041721	0.012285	187.290632	1,272,400.00
10	176.790793	0.064288	0.01893	267.266349	1,378,700.00
20	247.518937	0.090007	0.026504	354.360814	1,631,600.00
50	355.713351	0.12935	0.038089	481.32214	1,871,400.00

* From ecoinvent database, market for medium voltage electricity, GB 1 kWh = 0.294463 kg CO_2 eq

Ligand and Complex Syntheses

Synthesis of Catalyst 1[1]

Catalyst **1** was synthesized according to the following literature procedure:[1] A solution of pentafluorobenzoic acid (210 mg, 0.99 mmol), and (*R,R*)-(salcy)Co(II) complex (600 mg, 0.99 mmol) in toluene (15 mL) was stirred at 25 ℃ for 16 h. The solvent was removed *in vacuo* from the dark brown solution, and the resulting solid suspended in pentane (150 mL). The solution was then filtered and the dark green solid (624 mg, 77%) was dried *in vacuo*, then stored under a nitrogen atmosphere. Synthesis of the desired complex was confirmed by ^{1}H NMR and ^{13}C NMR spectroscopy, UV/Vis spectroscopy and IR spectroscopy (Supplementary Figures 1-7). ^{1}H NMR (500 MHz, DMSO-d_6, 298 K) δ 7.79 (s, 2H, N=C$\underline{H}$ (e)), 7.47 (m, 2H, *meta*-C(sp^2)-$\underline{H}$ (c, d)), 7.43 (m, 2H, *meta*-C(sp^2)-$\underline{H}$ (c, d)), 3.60 (m, 2H, N-C$\underline{H}$ (h)), 3.06 (m, 2H, C$\underline{H}_2$ (f, g)), 2.00 (m, 2H, C$\underline{H}_2$ (f, g)), 1.90 (m, 2H, C$\underline{H}_2$ (f, g)), 1.73 (s, 18H, C$\underline{H}_3$ (b)), 1.58 (m, 2H, C$\underline{H}_2$ (f, g)), 1.30 (s, 18H, C$\underline{H}_3$ (a)). ^{13}C NMR (126 MHz, DMSO-d_6, 298 K) δ 165.4 (*ipso*-$\underline{C}$(sp^2)-H (l)), 164.2 (O=$\underline{C}$ (m)), 161.7 (N=$\underline{C}$H (e)), 141.4 (*ortho*-$\underline{C}$(sp^2)-C (l)), 135.5 (*para*-$\underline{C}$(sp^2)-C (i)), 128.8 (*meta*-$\underline{C}$(sp^2)-C (c, d)), 128.4 (*meta*-$\underline{C}$(sp^2)-C (c, d)), 118.2 (*ortho*-$\underline{C}$(sp^2)-C (j)), 68.9 (N-$\underline{C}$H$_2$ (e)), 35.4 ($\underline{C}$-CH$_3$ (s)), 33.2 ($\underline{C}$-CH$_3$ (r)), 31.1 ($\underline{C}$H$_3$ (b)), 30.0 ($\underline{C}$H$_3$ (a)), 29.7 ($\underline{C}$-CH$_2$ (f, g)), 23.9 ($\underline{C}$-CH$_2$ (f, g)).

Syntheses of Catalysts 2 and 3[2]

Catalysts **2** and **3** were synthesized according to the literature procedure,[2] which is detailed here only for the synthesis for catalyst **3** (N.B. Catalyst **2** requires KOAc addition instead of NaOAc):

A solution of NaOAc (105 mg, 1.28 mmol), Co(OAc)$_2$ (227 mg, 1.28 mmol) and the proligand (500 mg, 1.28 mmol), in acetonitrile (15 mL) was prepared, under nitrogen atmosphere. The resulting orange solution was stirred at 25 ℃ for 30 mins, then ethylene diamine (85 µL, 1.28 mmol) was added resulting in a colour change to darkened orange/brown. The mixture was stirred for 16 h at 25 ℃, before it was opened to air and acetic acid was added (150 µL, 2.56 mmol) to achieve oxidation to the Co(III) species. The crude product was purified via azeotropic distillation with toluene (3 x 50 mL) and pentane (3 x 50 mL), and the resulting complex was precipitated from a dichloromethane solution with pentane (60:40 DCM: pentane). A dark brown solid was isolated (560 mg, 71%) and dried overnight in vacuo, then stored under a nitrogen atmosphere. Synthesis of the desired complex was confirmed by ^{1}H NMR and ^{13}C NMR spectroscopy, and IR spectroscopy (Supplementary Figures 8-21).

Catalyst 2 ^{1}H NMR (500 MHz, CDCl$_3$, 298 K) δ 7.70 (s, 2H, N=CH (i)), 6.84 (d, J = 7.8 Hz, 2H, meta-C(sp^2)-H (h, g)), 6.70 (d, J = 7.7 Hz, 2H, meta-C(sp^2)-H (h, g)), 6.40 (t,

J = 7.7 Hz, 2H, para-C(sp^2)-H (f)), 4.32 (m, 4H, N-CH_2 (e)), 4.18 (m, 4H, O-CH_2 (c, d)), 3.95 (m, 4H, O-CH_2 (c, d)), 3.84 (m, 4H, O-CH_2 (b)), 1.46 (s, 6H, O=C-CH_3 (a)).

Catalyst 2 ^{13}C {^{1}H} NMR (126 MHz, $CDCl_3$, 298 K) δ 179.6 (O=C (m)), 164.7 (N=CH (i)), 157.2 (ipso-C(sp^2)-H (l)), 152.1 (ortho-C(sp^2)-H (j)), 126.0 (meta-C(sp^2)-H (g, h)), 119.0 (ortho-C(sp^2)-C (k)), 112.6 (meta-C(sp^2)-H (g, h)), 112.3 (para-C(sp^2)-H (f)), 77.3 (O-CH_2 (b)), 70.3 (O-CH_2 (c, d)), 66.0 (O-CH_2 (c, d)), 59.3 (N-CH_2 (e)), 24.8 (O=C-CH_3 (a)).

Catalyst 3 ^{1}H NMR (500 MHz, $CDCl_3$, 298 K) δ 7.77 (s, 2H, N=CH (i)), 6.88 (d, 2H, meta-C(sp^2)-H (h, g)), 6.79 (d, 2H, meta-C(sp^2)-H (h, g)), 6.45 (t, J = 7.8 Hz, 2H, para-C(sp^2)-H (f)), 4.39 – 4.33 (m, 4H, N-CH_2 (e)), 4.24 – 4.17 (m, 4H, O-CH_2 (c, d)), 3.99 – 3.94 (m, 4H, O-CH_2 (c, d)), 3.90 – 3.84 (m, 4H, O-CH_2 (b)), 1.44 (s, 6H, O=C-CH_3 (a)).

Catalyst 3 ^{13}C {^{1}H} NMR (126 MHz, $CDCl_3$, 298 K) δ 179.6 (O=C (m)), 164.9 (N=CH (i)), 156.8 (ipso-C(sp^2)-H (l)), 152.1 (ortho-C(sp^2)-H (j)), 126.5 (meta-C(sp^2)-H (g, h)), 119.3 (ortho-C(sp^2)-C (k)), 114.9 (meta-C(sp^2)-H (g, h)), 112.9 (para-C(sp^2)-H (f)), 69.3 (O-CH_2 (b)), 69.1 (O-CH_2 (c, d)), 67.3 (O-CH_2 (c, d)), 59.1 (N-CH_2 (e)), 24.9 (O=C-CH_3 (a)).

Synthesis of Catalyst 4[5]

Catalyst **4** was synthesized according to the following literature procedure.[5] First the 2, 3-dihydroxybenzene-1,4-dicarbaldehyde was synthesized according to literature procedure:[6] TMEDA (9 mL, 60 mmol, 5 equiv) was added to a solution of 1,2-dimethoxybenzene (1.5 mL, 12 mmol) in diethyl ether (100 mL). The mixture was held in an ice bath (0 ℃). nBuLi 2.5 M in hexane (24 mL, 60 mmol) was added slowly over 2 min and the mixture stirred during reflux for 20 h. After the metalation, the reaction mixture was allowed to warm to room temperature and DMF (4.6 mL, 60 mmol) was added to the mixture and left to stir for 16 h. The reaction mixture was hydrolysed with water (30 mL) and 3 M HCl (5 mL). The organic layer was extracted with chloroform (3 x 15 mL) and dried (over $MgSO_4$), filtered and concentrated to give a reddish brown oil. The residue was chromatographed on silica gel resulting in a pale yellow solid (R_f = 0.61, $CHCl_3$). This was then recrystallized from dichloromethane and hexane (1:1) to give the pale yellow solid product of 2,3-dimethoxybenzene-1,4-dicarbaldehyde (630 mg, 27%). Synthesis of the desired compound was confirmed by ^{1}H NMR spectroscopy (Supplementary Figure 22). **^{1}H NMR** (400 MHz, $CDCl_3$, 298 K) δ 10.45 (s, 2H, O=C$\underline{H}$), 7.63 (s, 2H, C(sp^2)-$\underline{H}$), 4.06 (s, 6H, C$\underline{H}_3$).

Then, boron tribromide (1.2 mL, 0.01 mmol, 4 equiv) was added to a solution of the 2,3-dimethoxybenzene-1,4-dicarbaldehyde (630 mg, 3.24 mmol, 1 equiv) in dichloromethane (50 mL) under a nitrogen atmosphere. The reaction mixture was stirred for 4 h at room temperature, then water (50 mL) was added and the reaction mixture was stirred for a further 16 h. The mixture was extracted with chloroform (3x 40 mL), and the organic layer was dried ($MgSO_4$), filtered and concentrated to give a

dark yellow solid. The solid was recrystallized from chloroform/hexane to give the yellow solid product of 2,3-dihydroxybenzene-1,4-dicarbaldehyde (420 mg, 77%). Synthesis of the desired compound was confirmed by ^{1}H NMR spectroscopy (Supplementary Figure 23). **^{1}H NMR** (400 MHz, $CDCl_3$) δ 10.44 (s, 2H, O=C<u>H</u>), 8.01 (s, 2H, C(sp^2)-O<u>H</u>), 7.64 (s, 2H, C(sp^2)-<u>H</u>).

Finally, catalyst **4** was synthesized by metalation of the 2, 3-dihydroxybenzene-1,4-dicarbaldehyde:[5] To a solution of 2,3-dihydroxybenzene-1,4-dicarbaldehyde (420 mg, 2.52 mmol, 3 equiv) in methanol (15 mL) was added to a solution of $Ca(OAc)_2.H_2O$ (150 mg, 0.84 mmol, 1 equiv) in methanol (15 mL). The reaction mixture was stirred at 70 ℃, for 2 h, before a solution of (*R,R*)-1,2-cyclohexanediamine *L*-tartrate (730 mg, 2.78 mmol, 3.3 equiv), and K_2CO_3 (380 mg, 2.78 mmol, 3.3 equiv) in methanol (15 mL), was added. The reaction mixture was stirred at 70 ℃ for 18 h. The solvent was removed *in vacuo* from the bright red solution and then redissolved in a minimum volume of chloroform before being filtered. The solvent was again removed *in vacuo* and the residue was redissolved in methanol (25 mL). A solution of $Co(OAc)_2.4H_2O$ (630 mg, 2.52 mmol, 3 equiv) in methanol (15 mL) was added and the resulting mixture was stirred at 70 ℃ for 2 h. The solvent was removed *in vacuo* from the dark red-purple solution, yielding a reddish purple solid (820 mg, 86%). Melting point analysis (> 300 ℃, matching reported literature value[5]) was completed, and UV/Vis and IR spectra were obtained (Supplementary Figures 24 and 25).

Synthesis of Catalyst 5[3]

Catalyst **5** was synthesized according to the previously reported procedure:[3] Ethylenediamine (93 µL, 1.28 mmol) was added to a solution of 2-hydroxy-3-(2-methoxyethoxy)benzaldehyde (500 mg, 2.6 mmol) in methanol (100 mL). The resulting bright yellow solution was stirred for 16 h before the solvent was removed *in vacuo* and the product precipitated from a dichloromethane solution with pentane. The residue was dried under vacuum for 16 h (556 mg, 52 %). Next a solution of KOAc (118 mg, 1.2 mmol), $Co(OAc)_2$ (213 mg, 1.2 mmol) and the proligand (500 mg, 1.2 mmol) in acetonitrile (120 mL) was prepared under nitrogen atmosphere. The resulting orange solution was stirred at 25 ℃ for 16 h before it was opened to air and acetic acid was added (137 µL, 2.4 mmol) to achieve oxidation to the Co(III) species. The reaction mixture was stirred at 25 ℃ for 72 h. The crude product was purified via azeotropic distillation with toluene (3x 50 mL) and pentane (3x 50 mL), and the resulting complex was precipitated from a dichloromethane solution with pentane. A light brown solid was isolated (355 mg, 71%) and dried overnight *in vacuo*, then stored under a nitrogen atmosphere. Synthesis of the desired complex was confirmed by ^{1}H NMR and ^{13}C NMR spectroscopy, and IR spectroscopy (Supplementary Figures 26-32). **^{1}H NMR** (500 MHz, $CDCl_3$, 298 K) δ 7.65 (s, 2H, N=C<u>H</u> (i)), 6.87 (d, *J* = 7.9 Hz, 2H, *meta*-C(sp^2)-<u>H</u> (h, g)), 6.78 (d, *J* = 7.7 Hz, 2H, *meta*-C(sp^2)-<u>H</u> (h, g)), 6.41 (t, *J* = 7.7 Hz, 2H, *para*-C(sp^2)-<u>H</u> (f)), 4.32 (s, 4H, N-C<u>H</u>$_2$ (e)), 4.21 – 4.13 (m, 4H, O-C<u>H</u>$_2$ (d)), 3.86 – 3.78 (m, 4H, O-C<u>H</u>$_2$ (c)), 3.48 (s, 6H, O-C<u>H</u>$_3$ (b)), 1.44 (s, 6H, O=C-C<u>H</u>$_3$ (a)). **^{13}C {^{1}H} NMR** (126 MHz, $CDCl_3$, 298 K) δ 179.4 (O=<u>C</u> (m)), 164.8 (N=<u>C</u>H (i)), 158.0 (*ipso*-<u>C</u>(sp^2)-H

(l)), 152.2 (*ortho*-C(sp^2)-H (j)), 138.0 (*meta*-C(sp^2)-H (g, h)), 127.0 (*meta*-C(sp^2)-H (g, h)), 119.4 (*ortho*-C(sp^2)-C (k)), 115.3 (*meta*-C(sp^2)-H (g, h)), 112.4 (*para*-C(sp^2)-H (f)), 70.9 (O-CH_2 (c)), 67.3 (O-CH_2 (d)), 59.1 (N-CH_2 (e)), 58.4 (O-CH_3 (b)), 24.8 (O=C-CH_3 (a)).

Additional Information

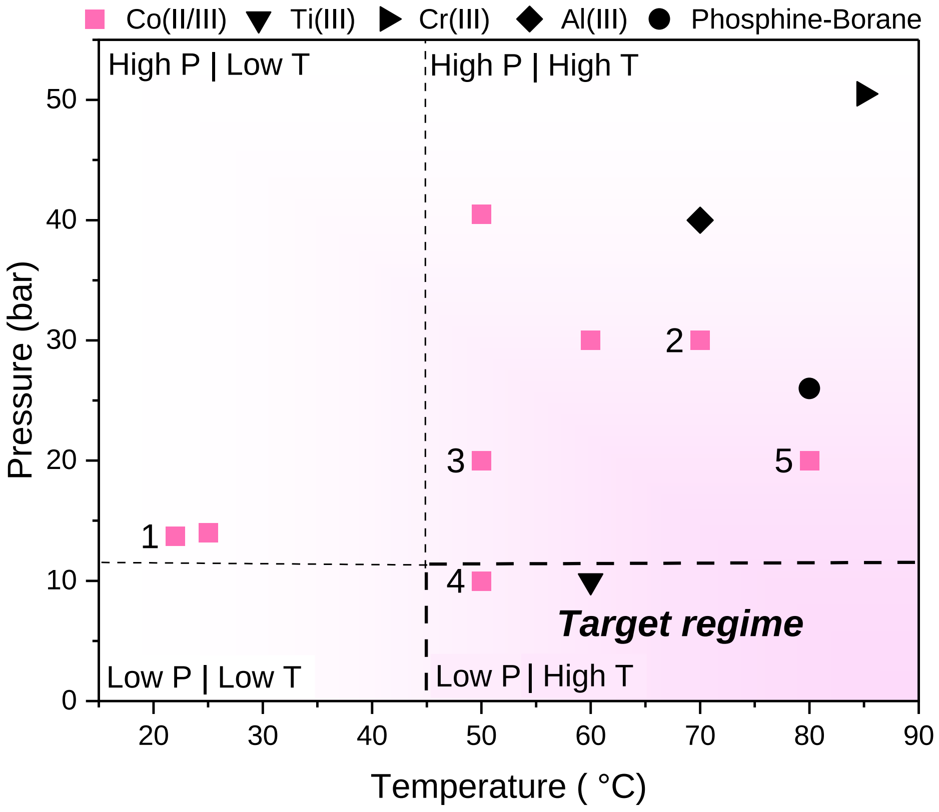

Supplementary Figure 1 Overview of reported catalysts for PO–CO_2 ROCOP, according to their optimum operating conditions (pressure and temperature).

Optimum operating conditions are defined as the conditions for which the highest activity and selectivity were reported in the original publications. The data and structures associated with this plot is listed in Supplementary Table 2.

Supplementary Table 2 Leading Catalysts for PO–CO_2 ROCOP

Catalyst	P / bar	T / °C	Reported below 10 bar	Author and Year
$L_1Co(III)$ + PPNCl (**1**) 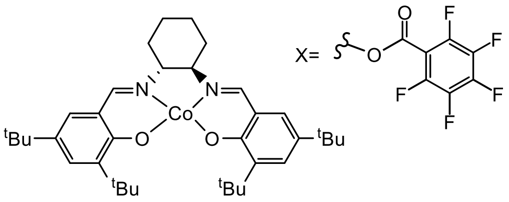	13.7	22	N	Coates, 2005[1]
$L_2Co(III)K(I)$ (**2**) 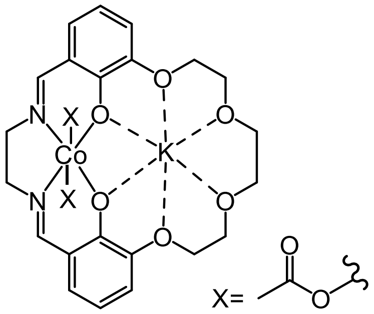	30	70	Y	Williams, 2020[2]
$L_2Co(III)Na(I)$ (**3**) 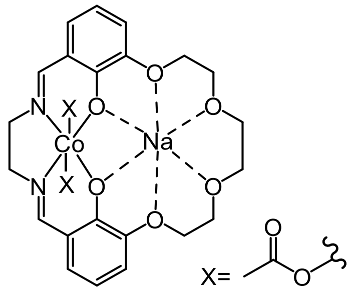	20	50	Y	Williams, 2020[2]
$L_3Co(II)_3Ca(II)$ + $[PhNMe_2H][B(C_6F_5)_4]$ (**4**) 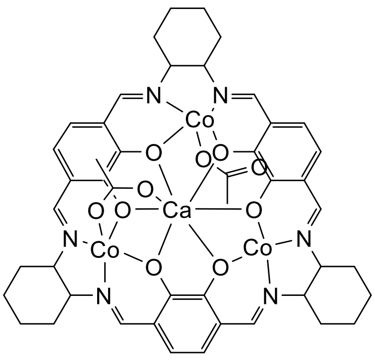	10	50	Y	Mashima, 2023[5]

L_4Co(III)K(I) open (**5**)<br>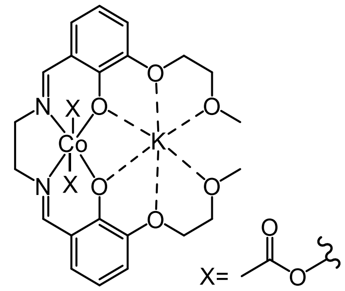	20	80	Y	Williams, 2024[3]
L_5Ti(IV) + PPNCl<br>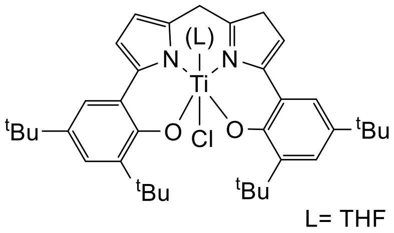	10	60	Y	Nozaki, 2011[7]
L_6Co(III)<br>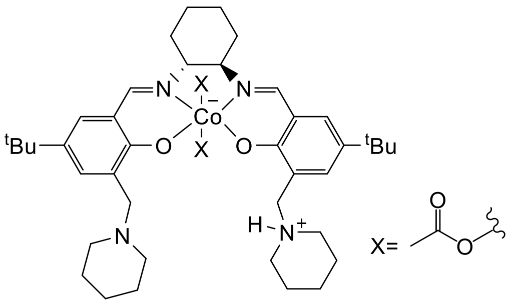	14	25	N	Nozaki, 2006[8]
Phosphine borane	26	80	N	Zhang, 2023[9]
L_7Al(III)<br>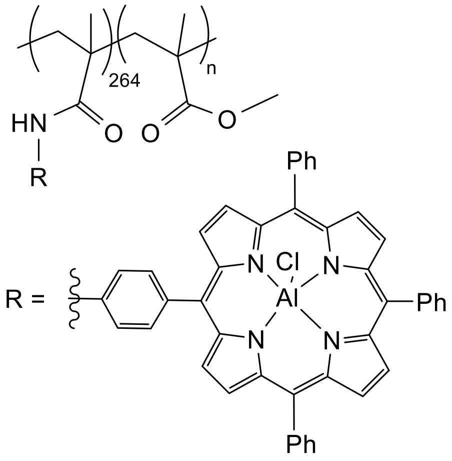	40	70	N	Wang, 2023[10]
L_8Co(III)	40.5	50	Y	Wang, 2012[11]

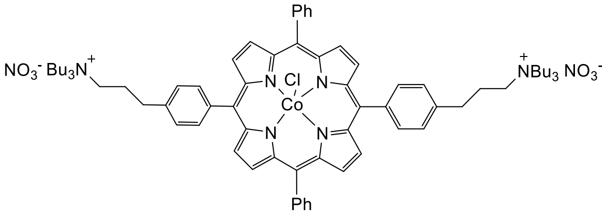				
$L_9Cr(III)$ 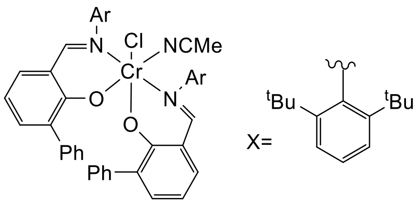	50.5	85	N	Darensbourg, 2003[12]

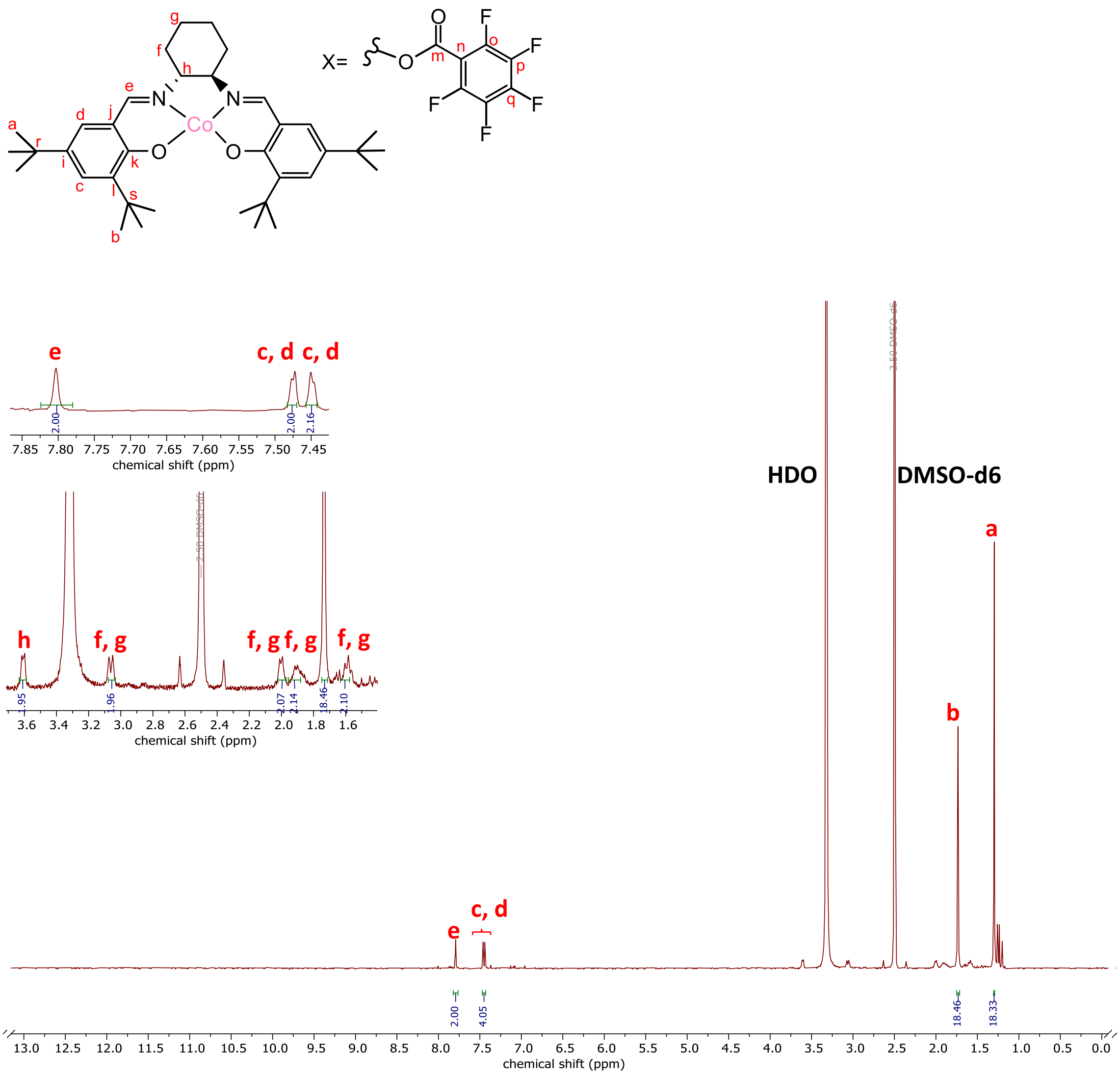

Supplementary Figure 2 ^{1}H NMR Spectrum of Catalyst 1.

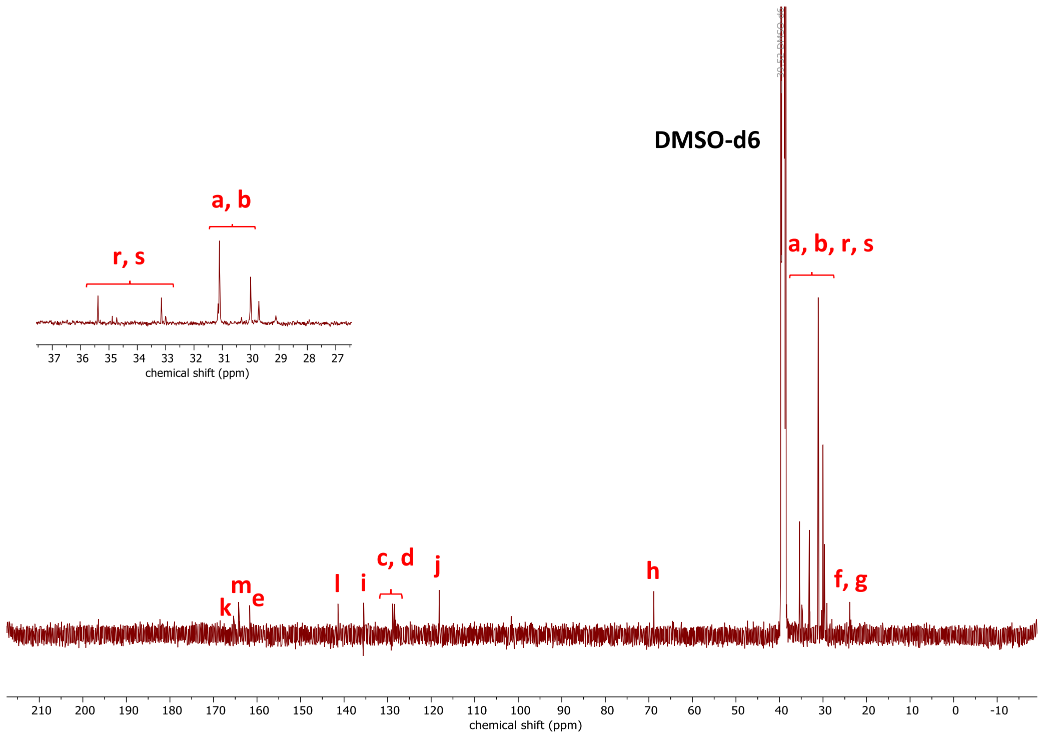

Supplementary Figure 3 $^{13}C^{13}$ NMR Spectrum of Catalyst 1

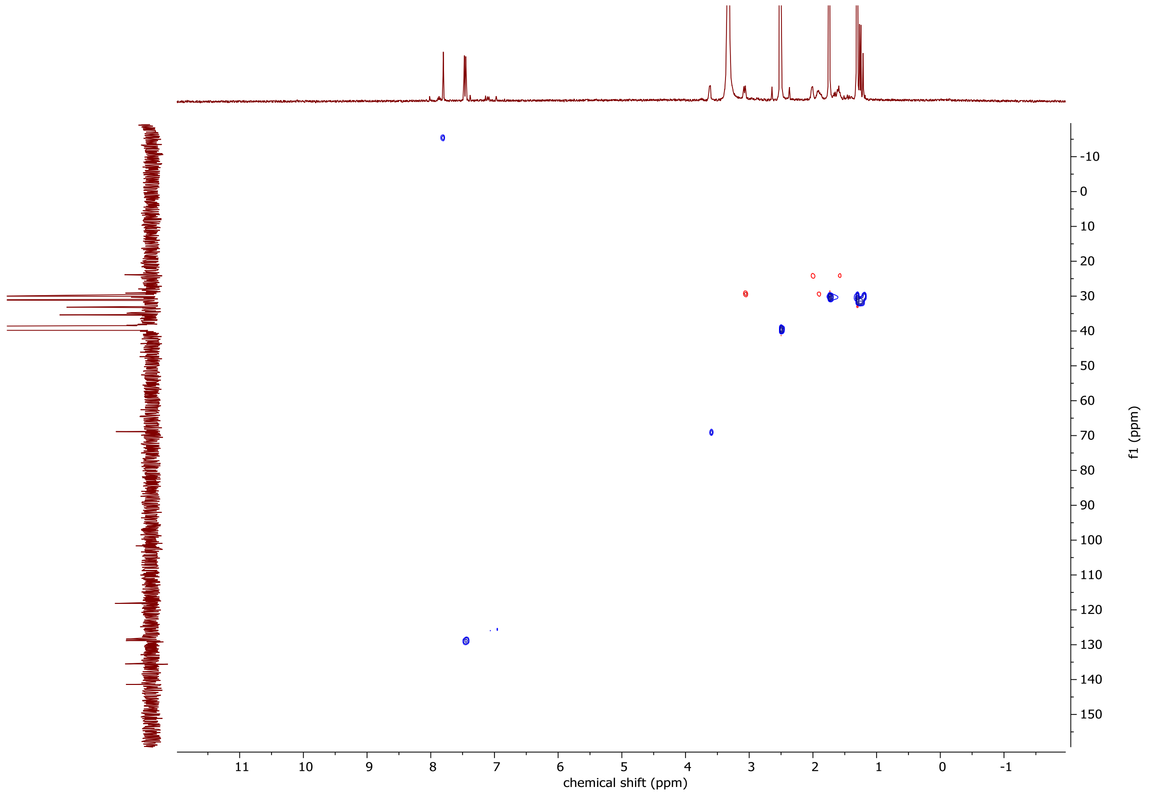

Supplementary Figure 4 2D ^{1}H -^{13}C HSQC Spectrum of Catalyst 1.

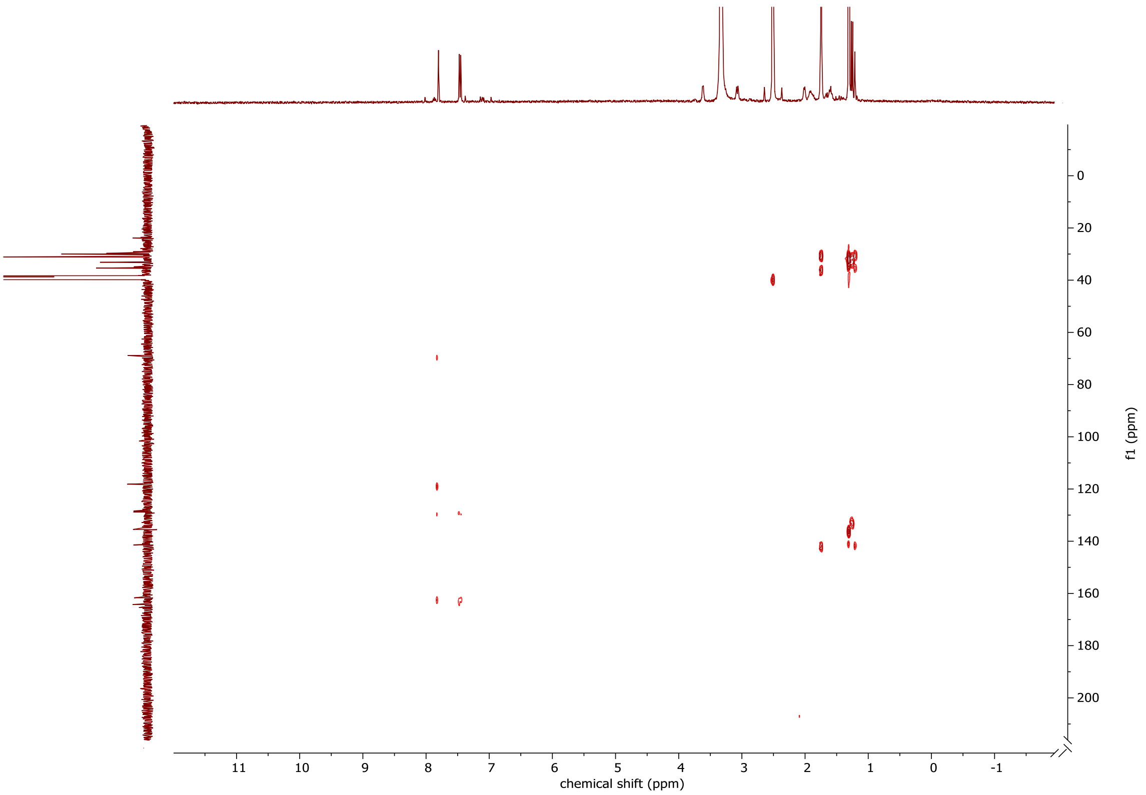

Supplementary Figure 5 2D ^{1}H -^{13}C HMBC Spectrum of Catalyst 1.

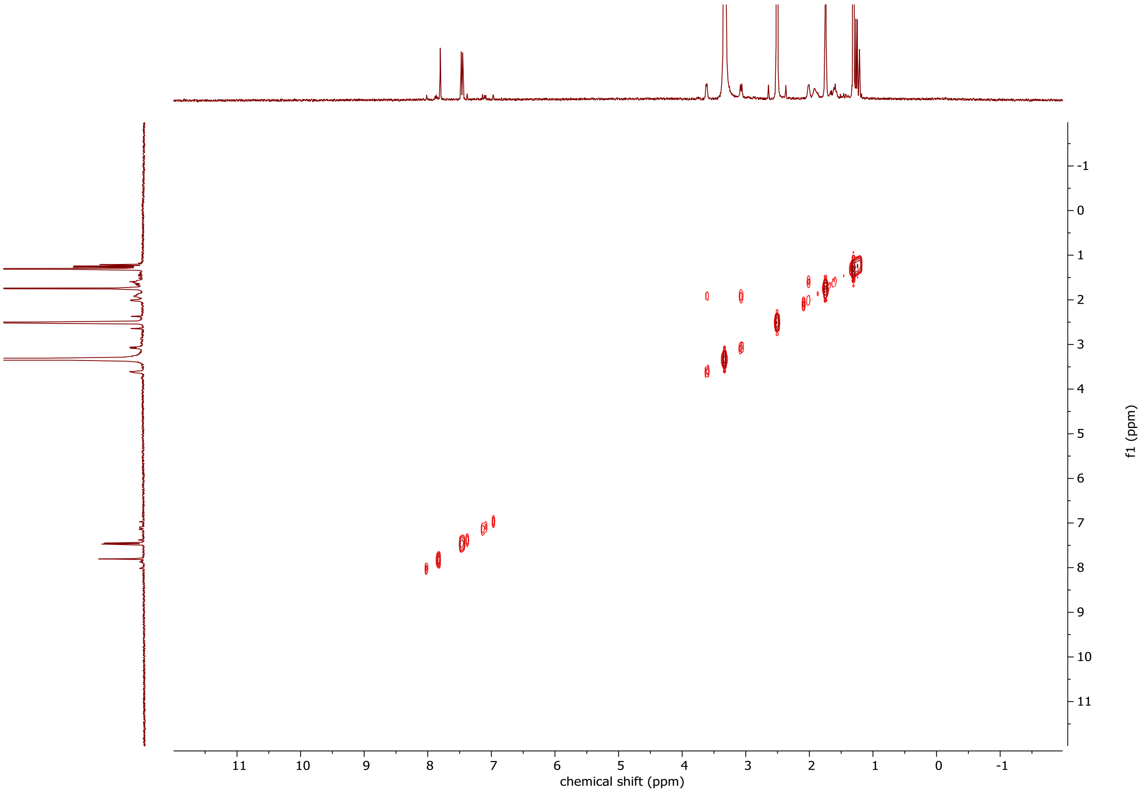

Supplementary Figure 6 2D ^{1}H -^{1}H COSY Spectrum of Catalyst 1.

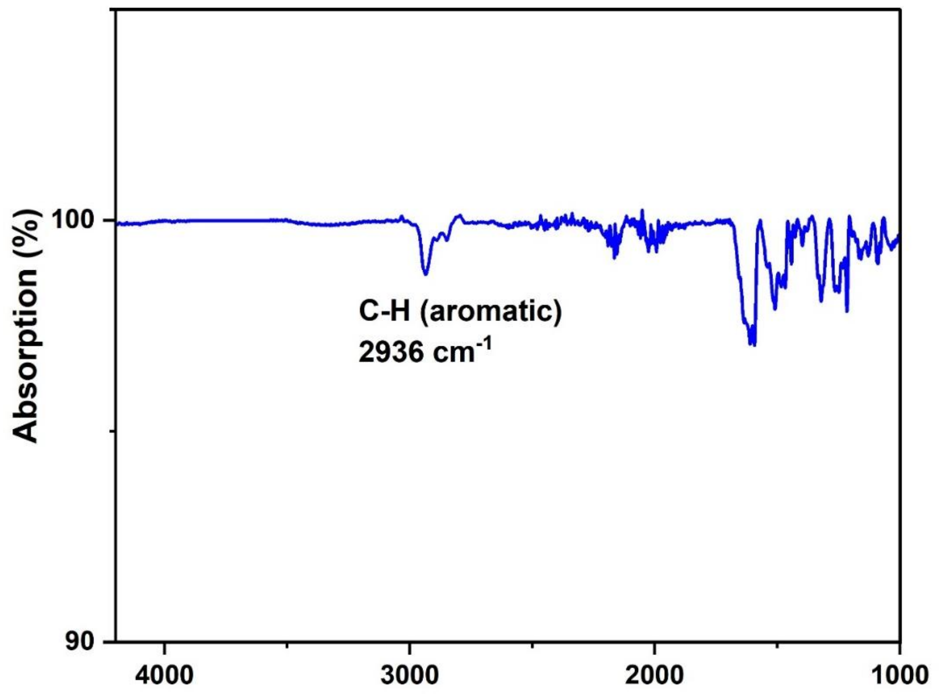

Supplementary Figure 7 IR Spectrum of Catalyst 1.

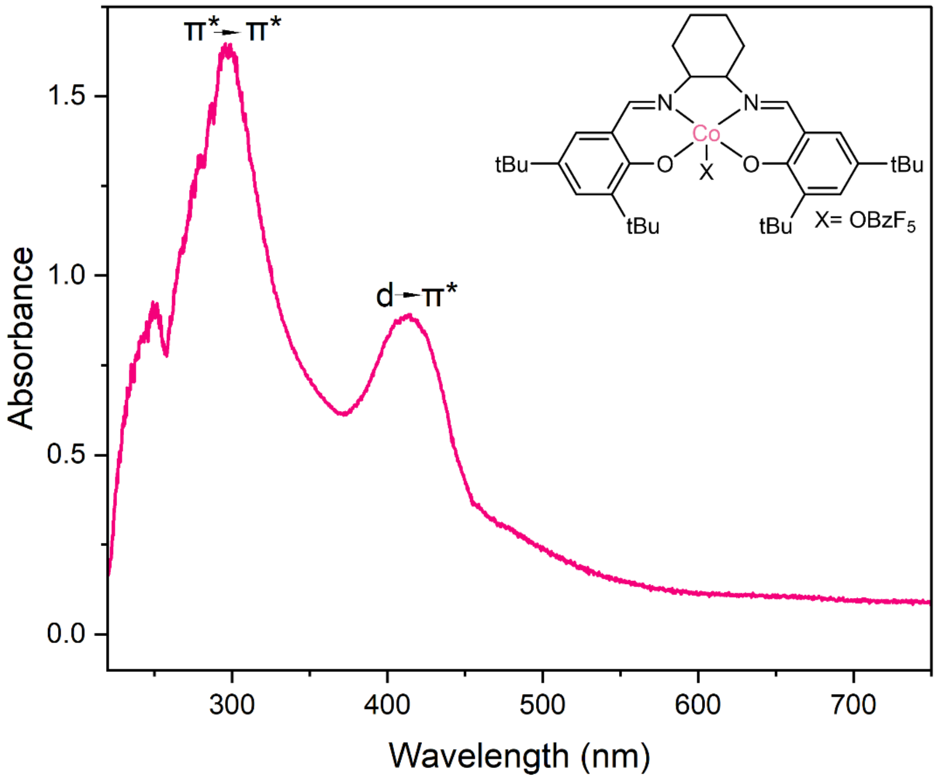

Supplementary Figure 8 UV/Vis Spectrum of Catalyst 1 (0.125 mM in MeCN).

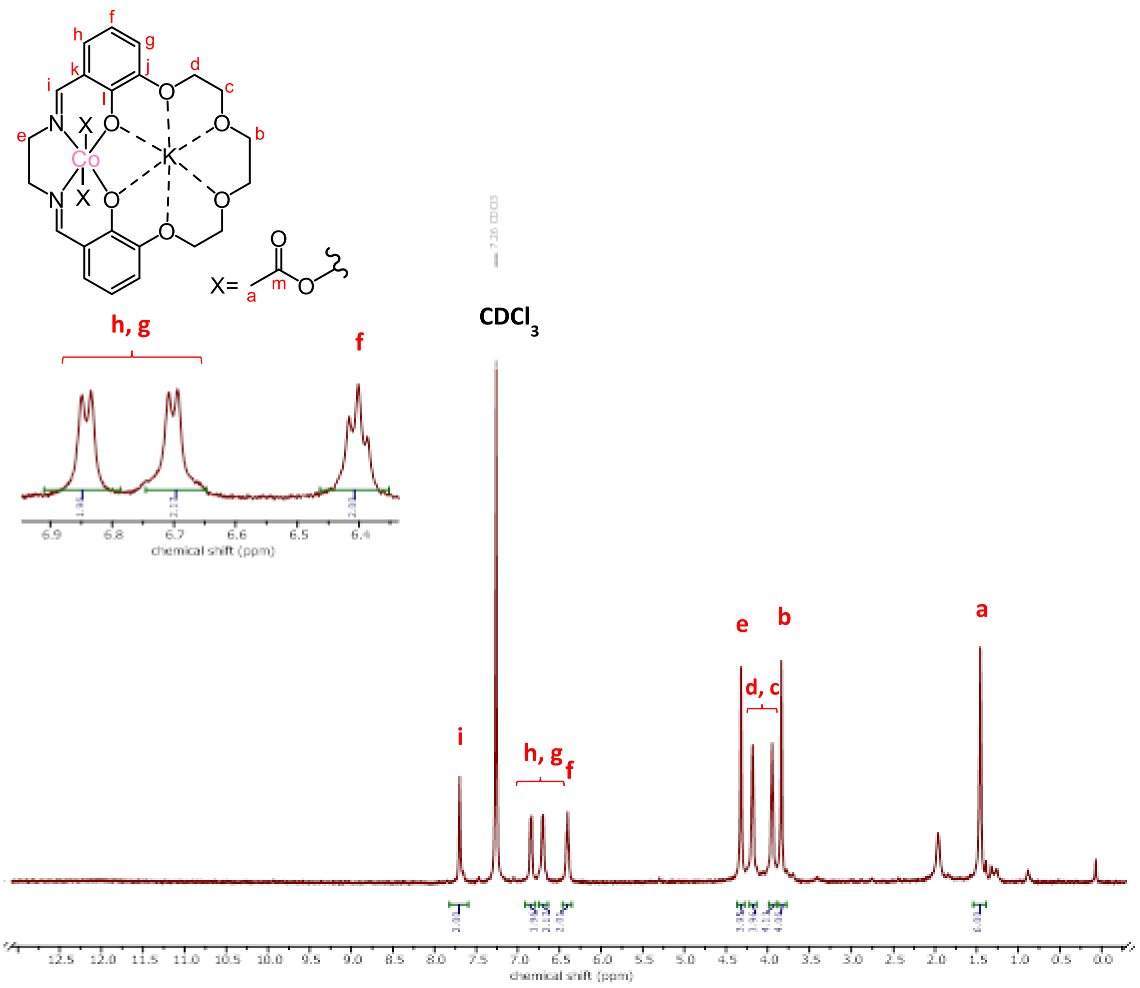

Supplementary Figure 9 ^{1}H NMR Spectrum of Catalyst 2.

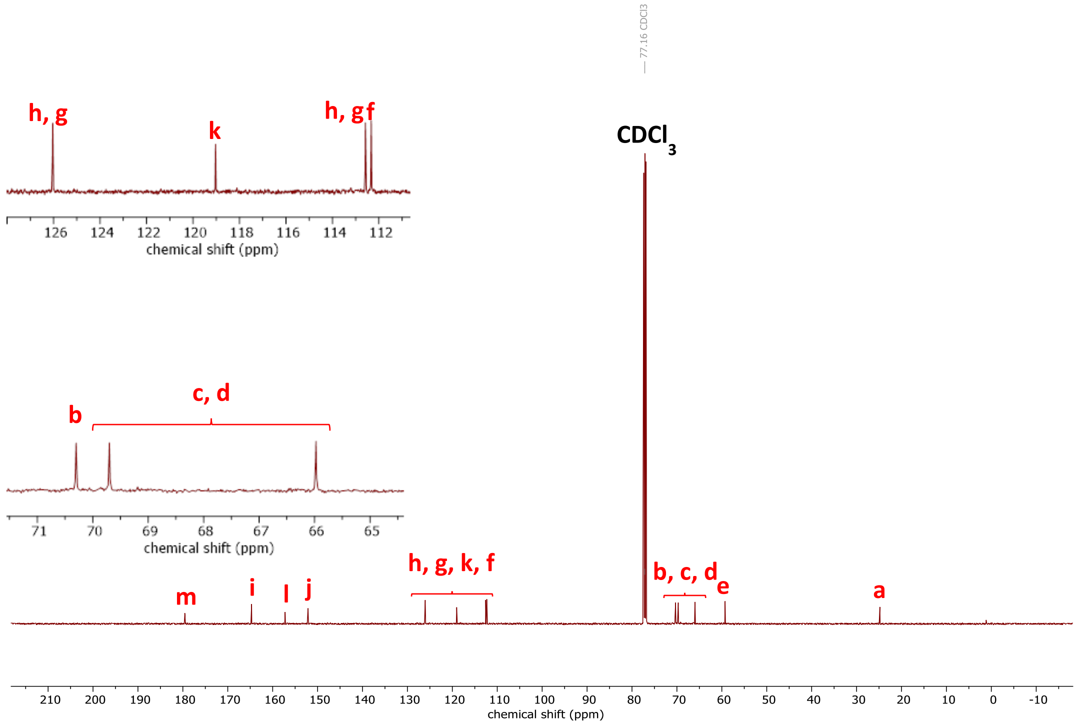

Supplementary Figure 10 ^{13}C{^{1}H} NMR Spectrum of Catalyst 2.

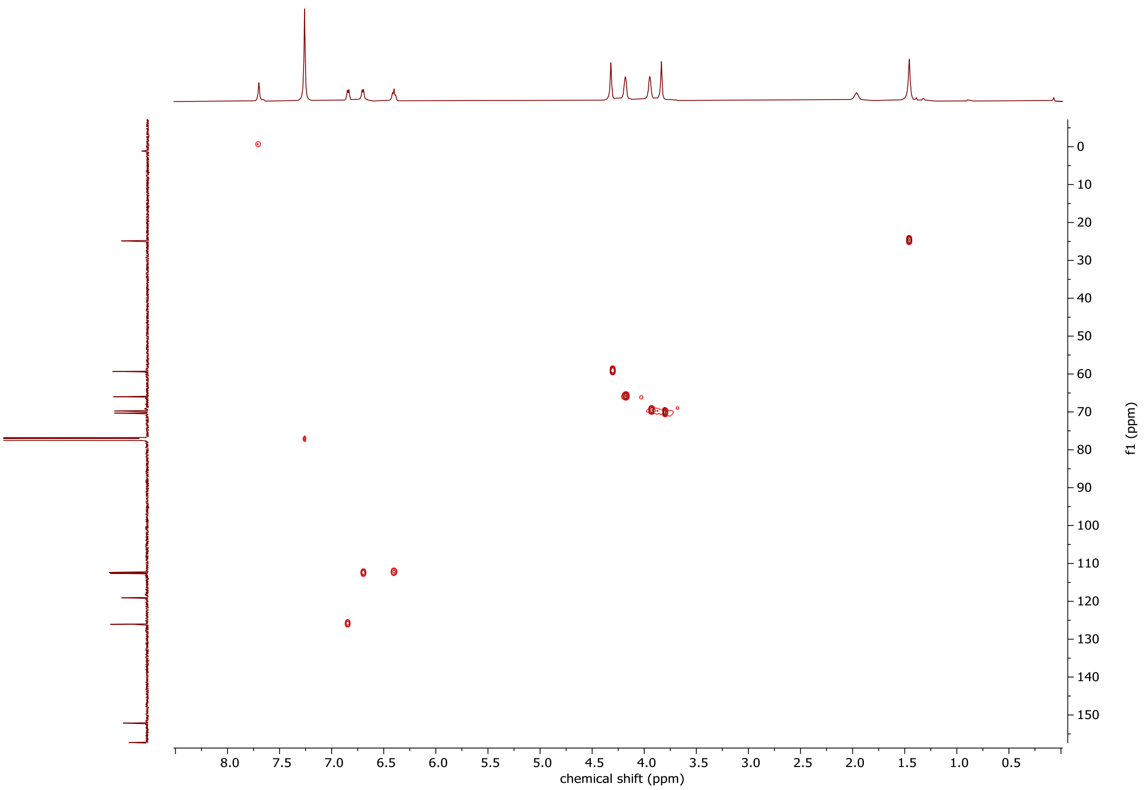

Supplementary Figure 11 2D ^{1}H -^{13}C HSQC Spectrum of Catalyst 2.

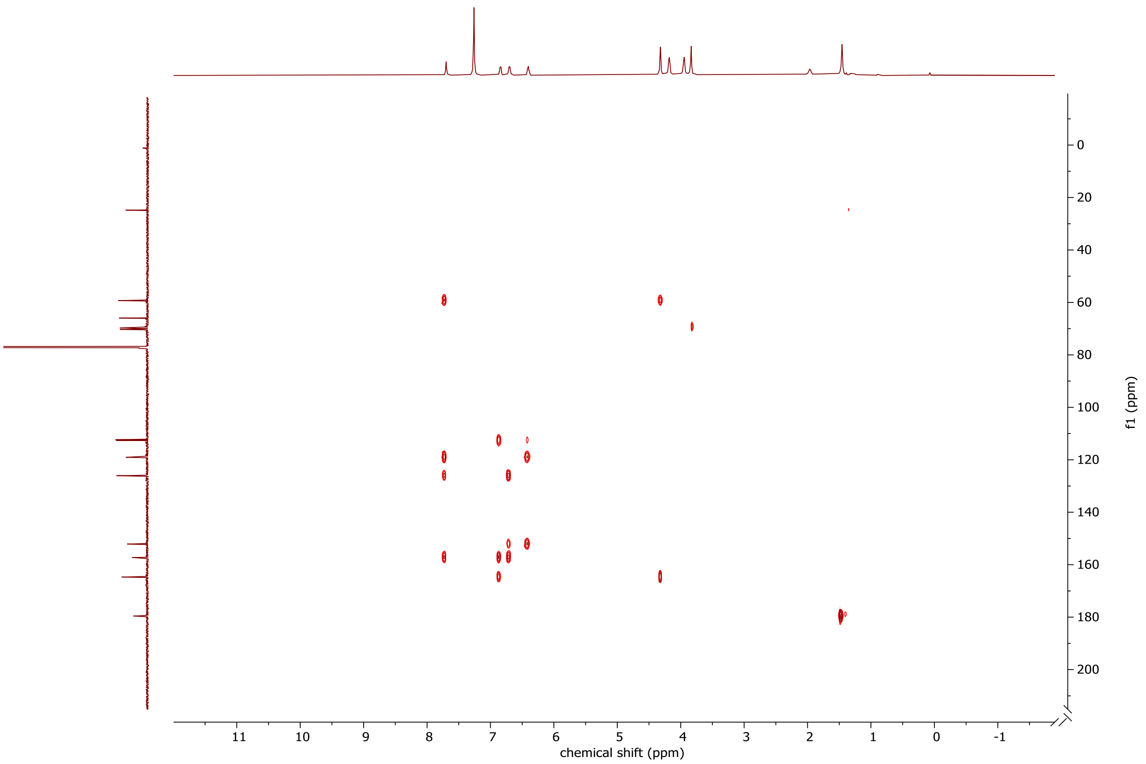

Supplementary Figure 12 2D ^{1}H -^{13}C HMBC Spectrum of Catalyst 2.

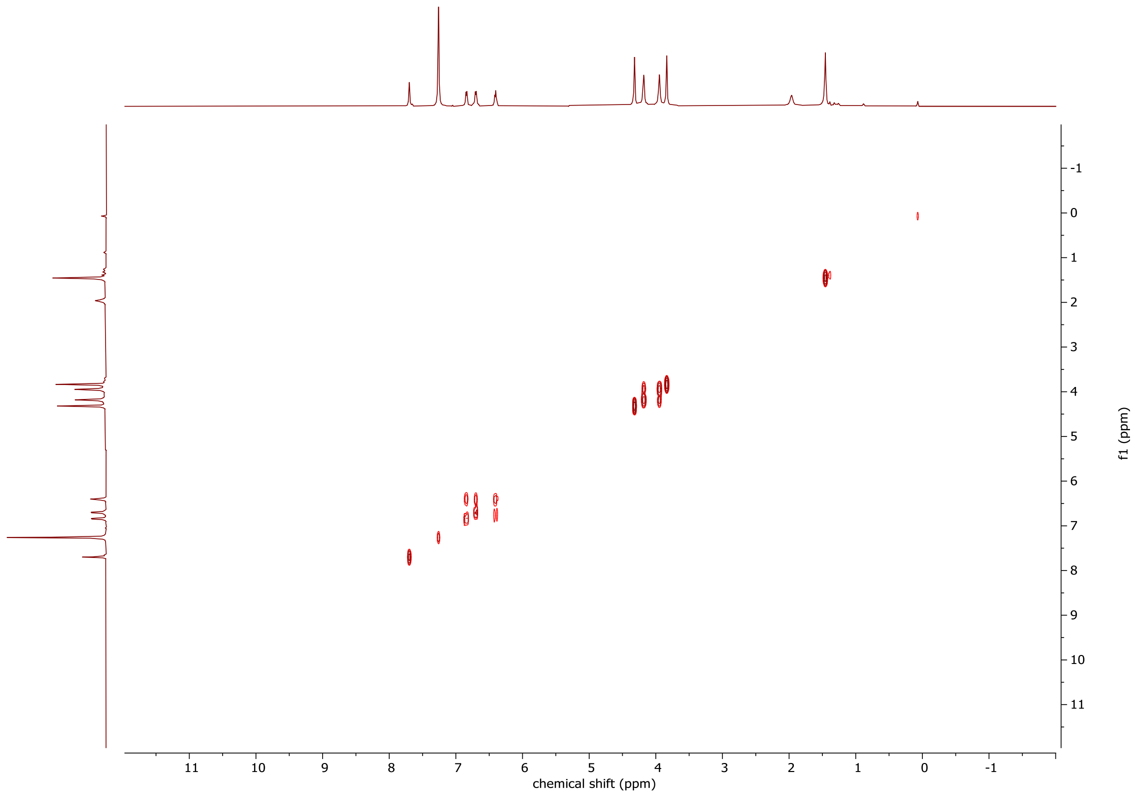

Supplementary Figure 13 2D ^{1}H -^{1}H COSY Spectrum of Catalyst 2.

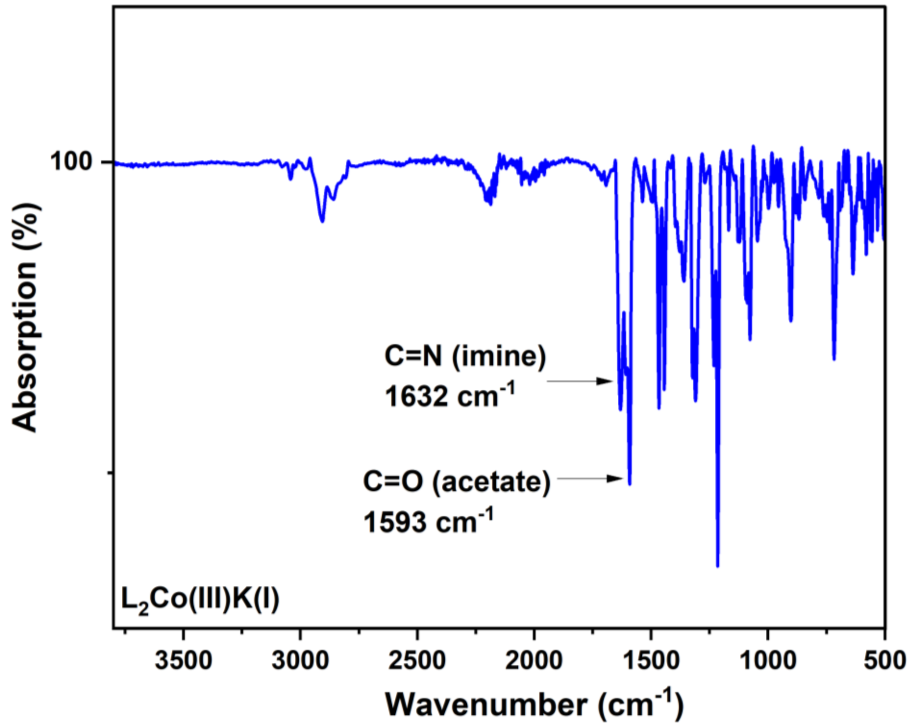

Supplementary Figure 14 IR Spectrum of Catalyst 2.

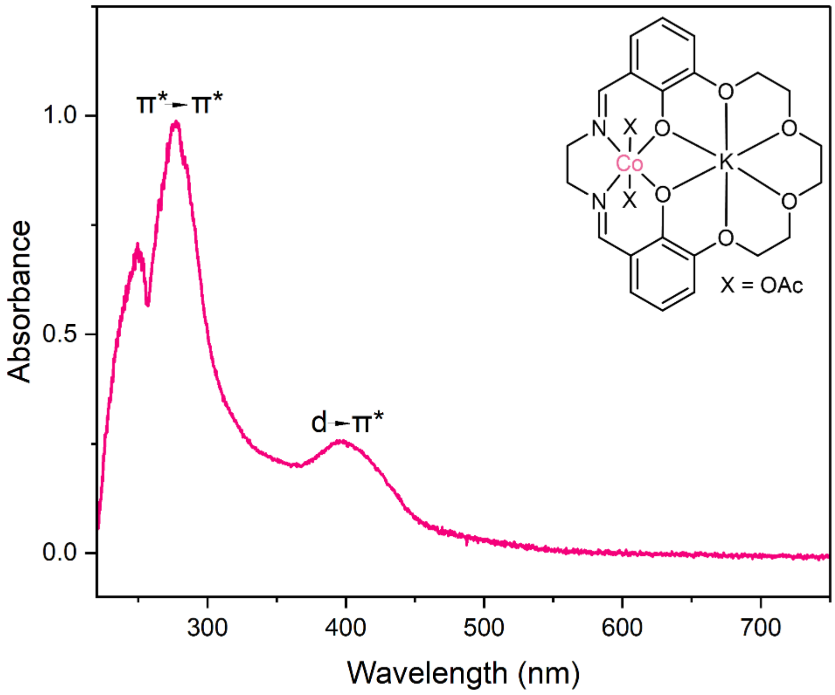

Supplementary Figure 15 UV/Vis Spectrum of Catalyst 2 (0.125 mM in MeCN).

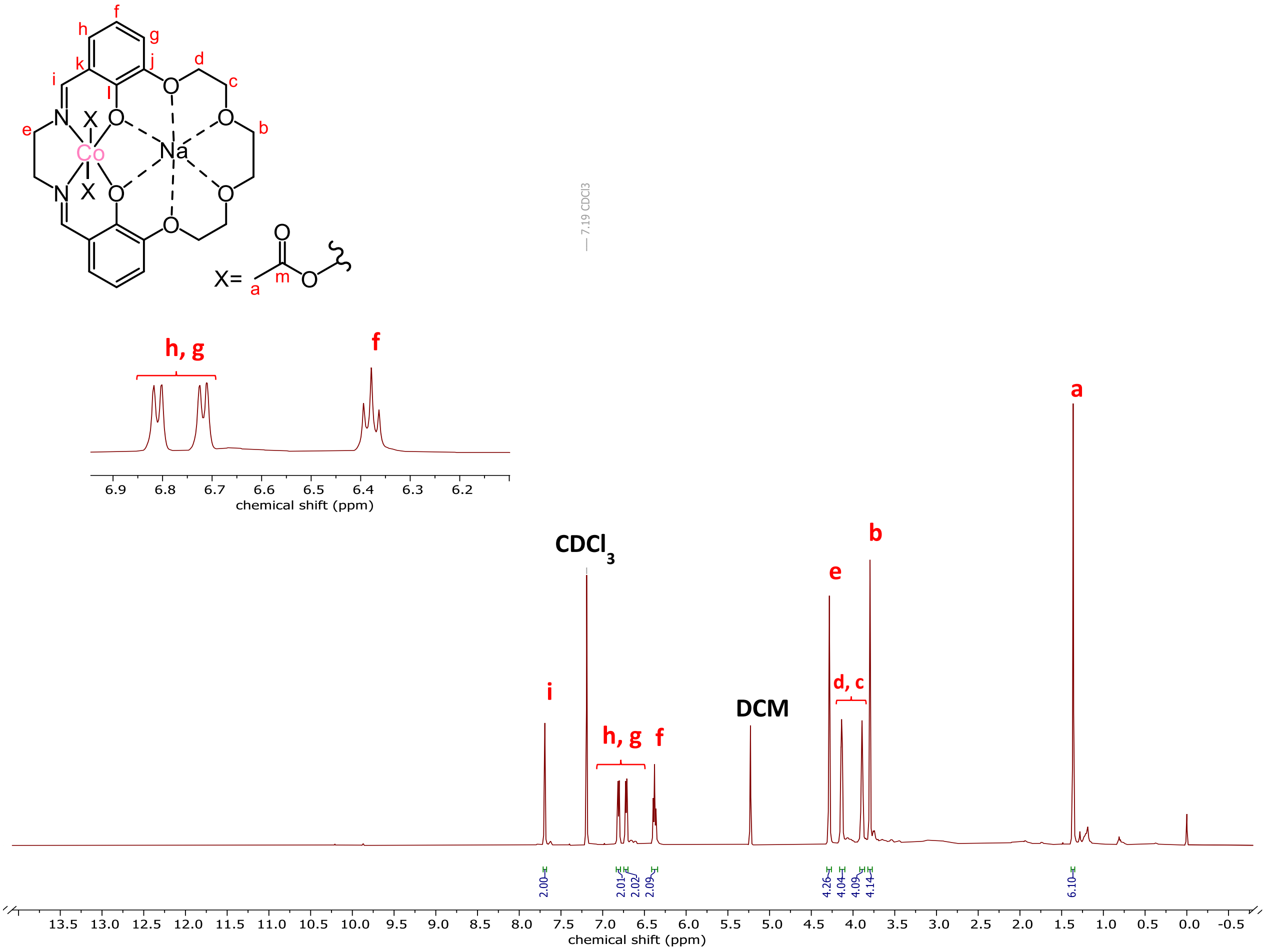

Supplementary Figure 16 2D ^{1}H NMR Spectrum of Catalyst 3.

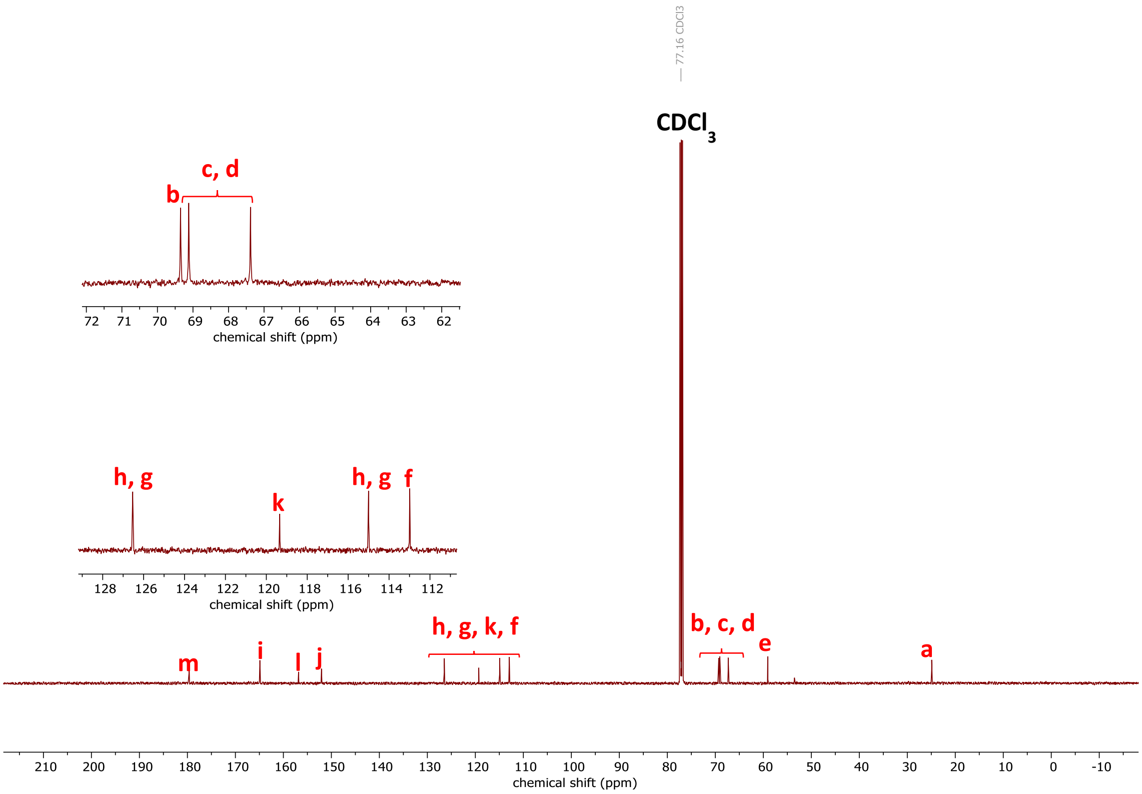

Supplementary Figure 17 $^{13}C\{^{1}H\}$ NMR Spectrum of Catalyst 3.

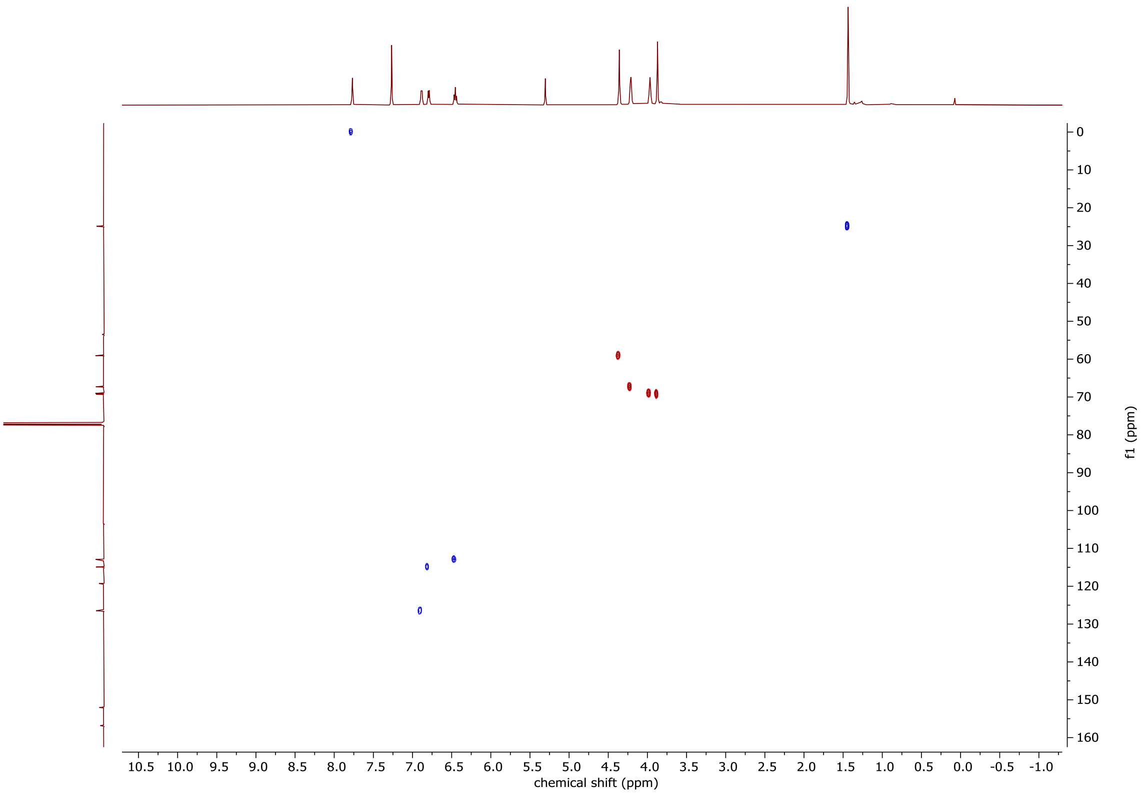

Supplementary Figure 18 2D ^{1}H -^{13}C HSQC Spectrum of Catalyst 3.

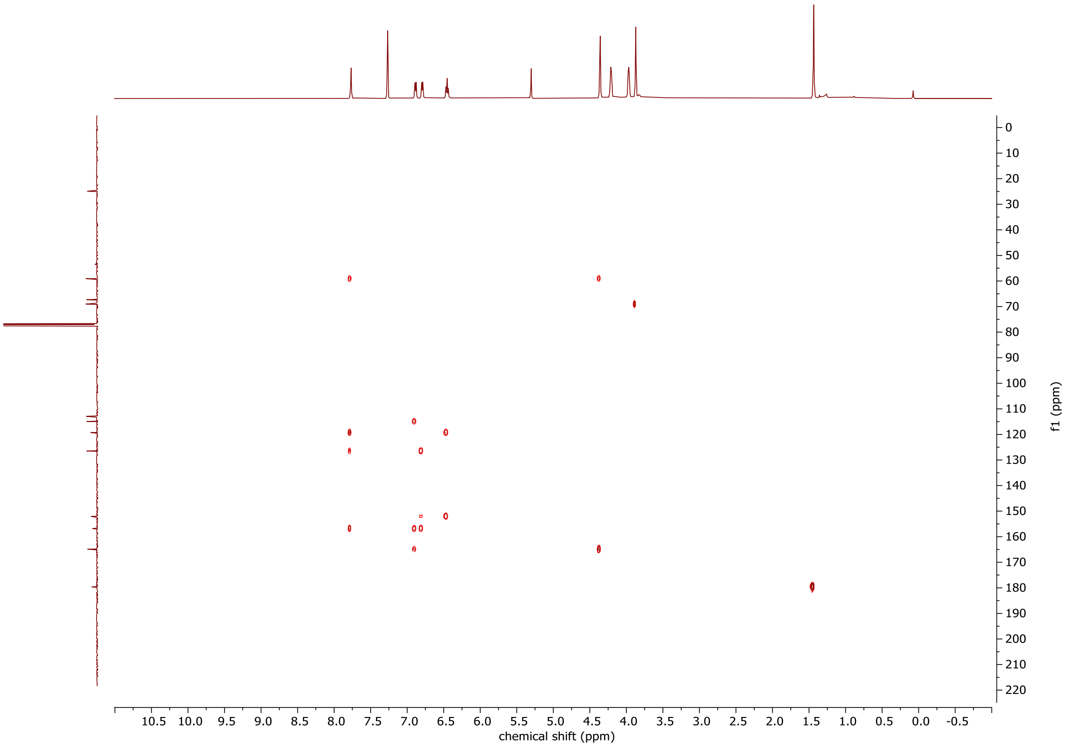

Supplementary Figure 19 2D ^{1}H -^{13}C HMBC Spectrum of Catalyst 3.

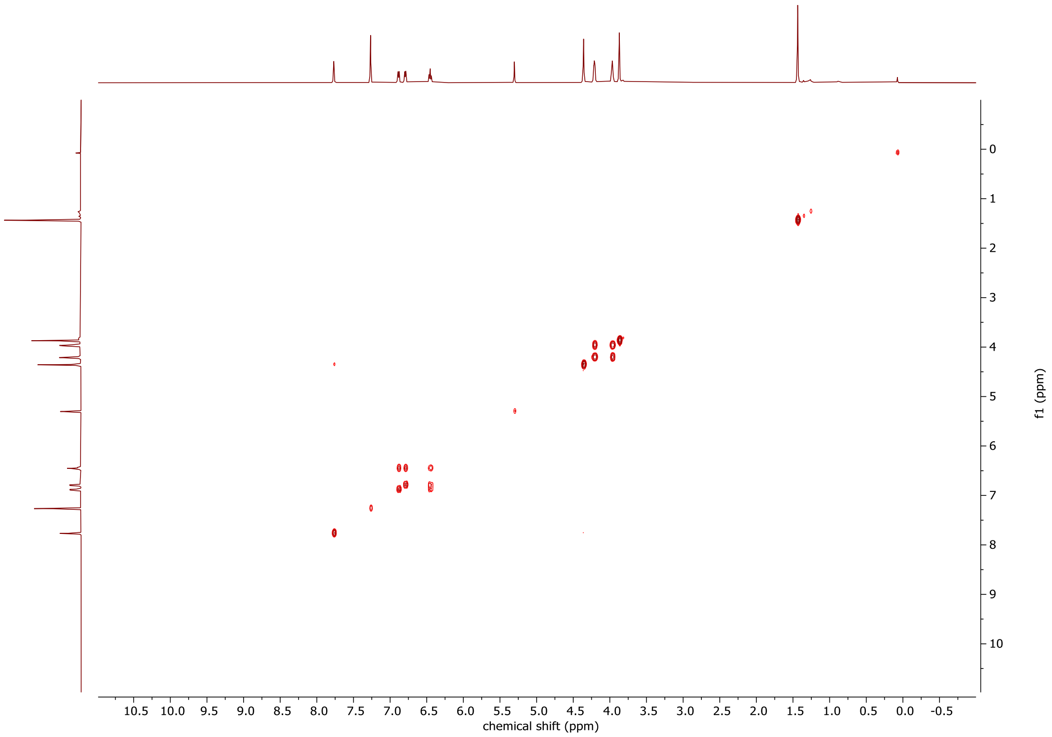

Supplementary Figure 20 2D ^{1}H -^{1}H COSY Spectrum of Catalyst 3.

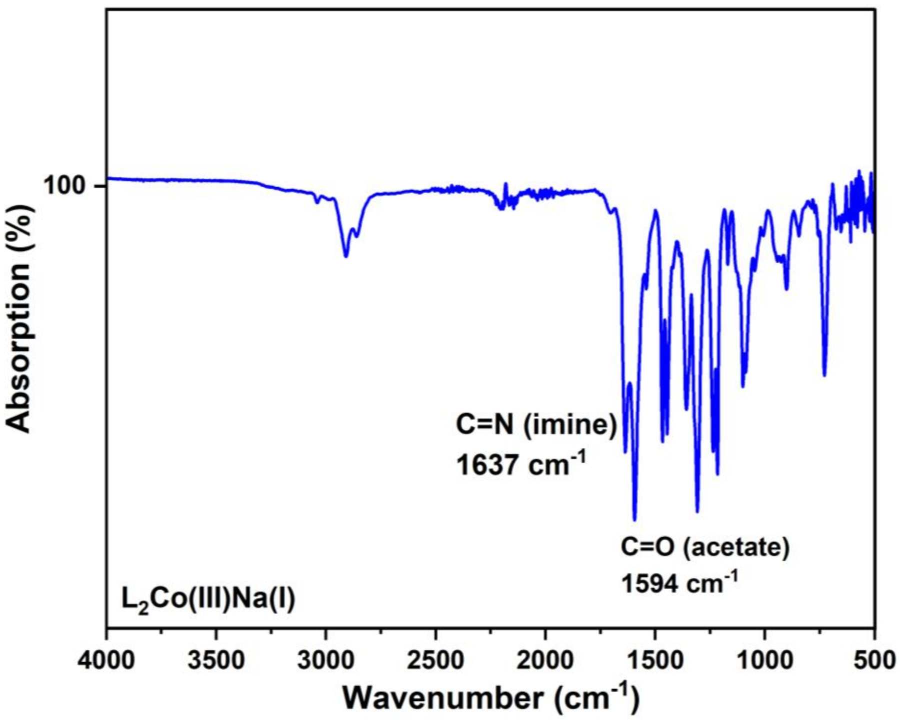

Supplementary Figure 21 IR Spectrum of Catalyst 3.

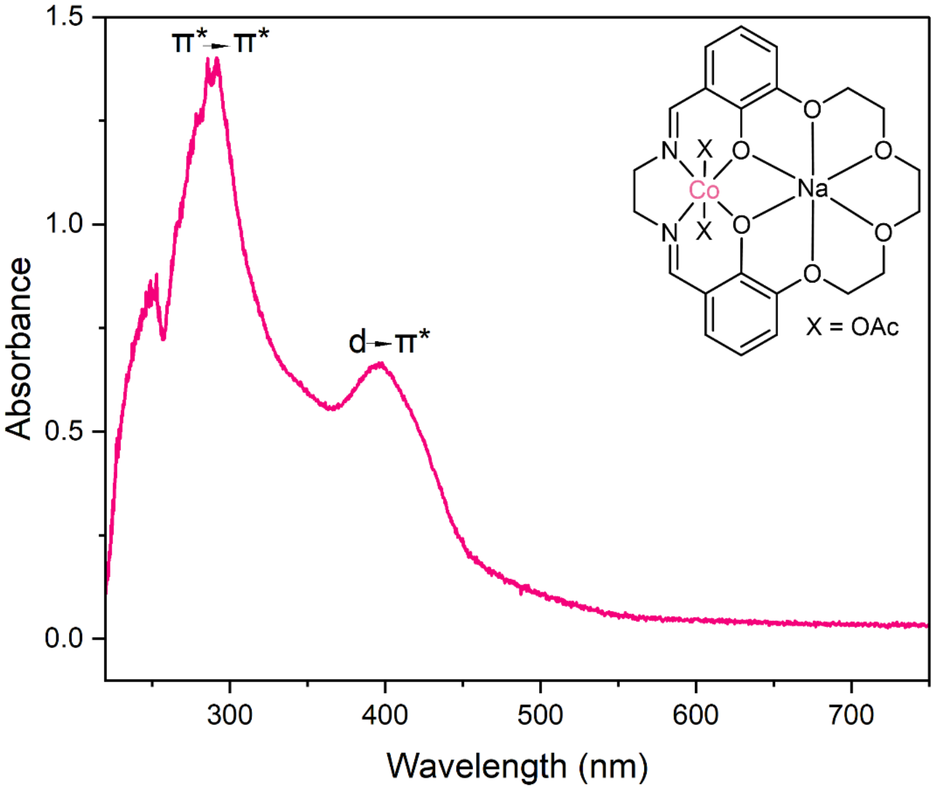

Supplementary Figure 22 UV/Vis Spectrum of Catalyst 3 (0.125 mM in MeCN).

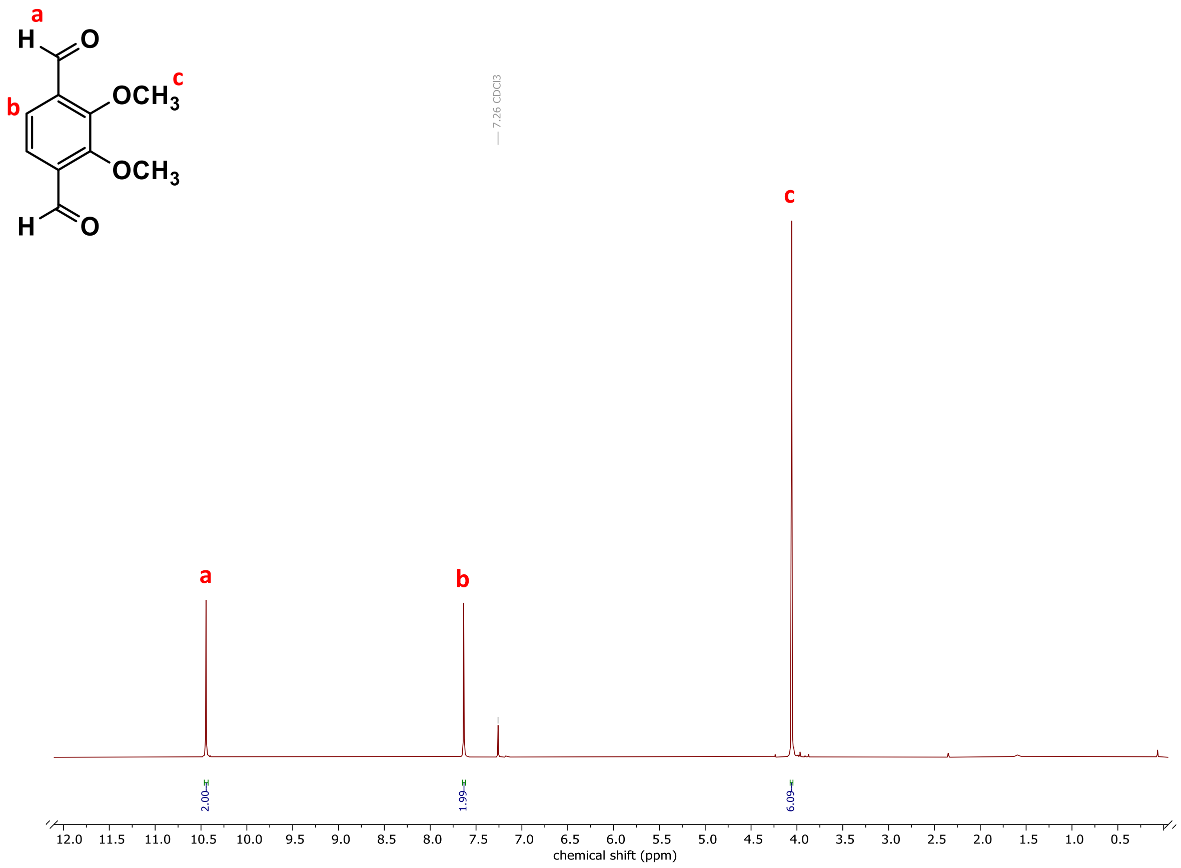

Supplementary Figure 23 ^{1}H NMR Spectrum of 2,3-dimethoxybenzene-1,4-dicarbaldehyde.

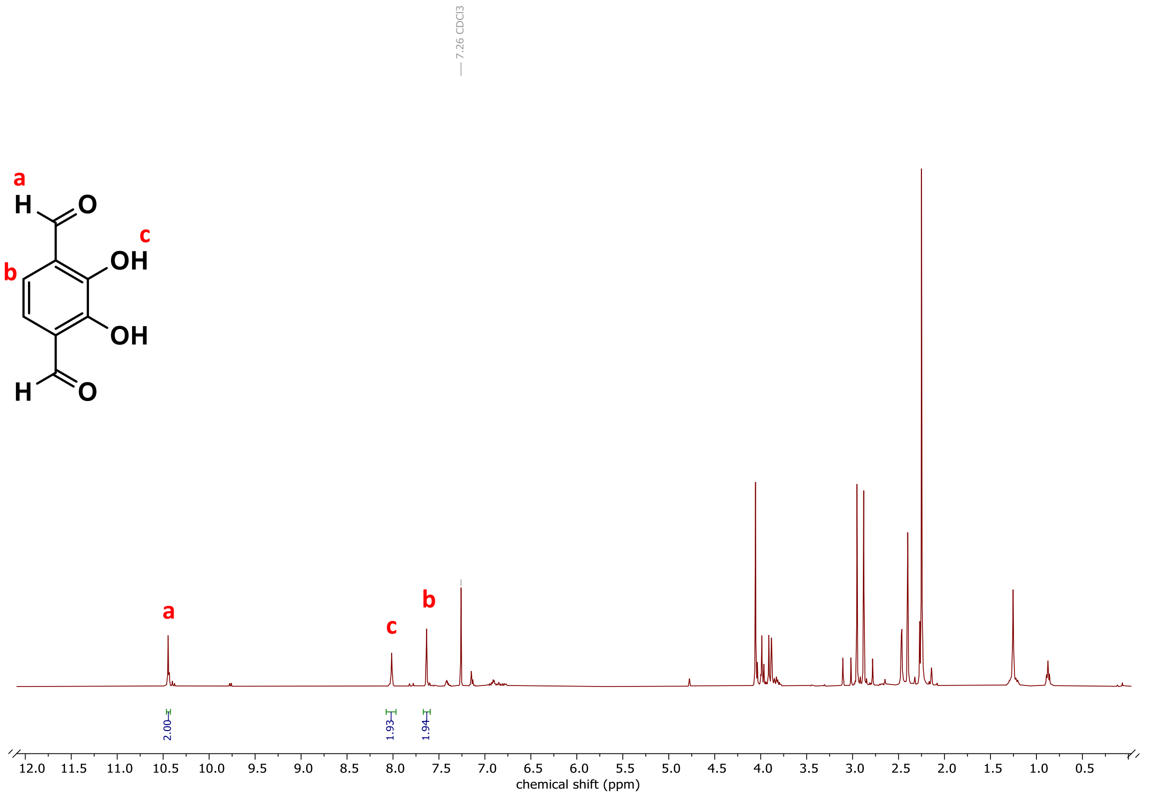

Supplementary Figure 24 ^{1}H NMR Spectrum of crude 2,3-dihydroxybenzene-1,4-dicarbaldehyde.

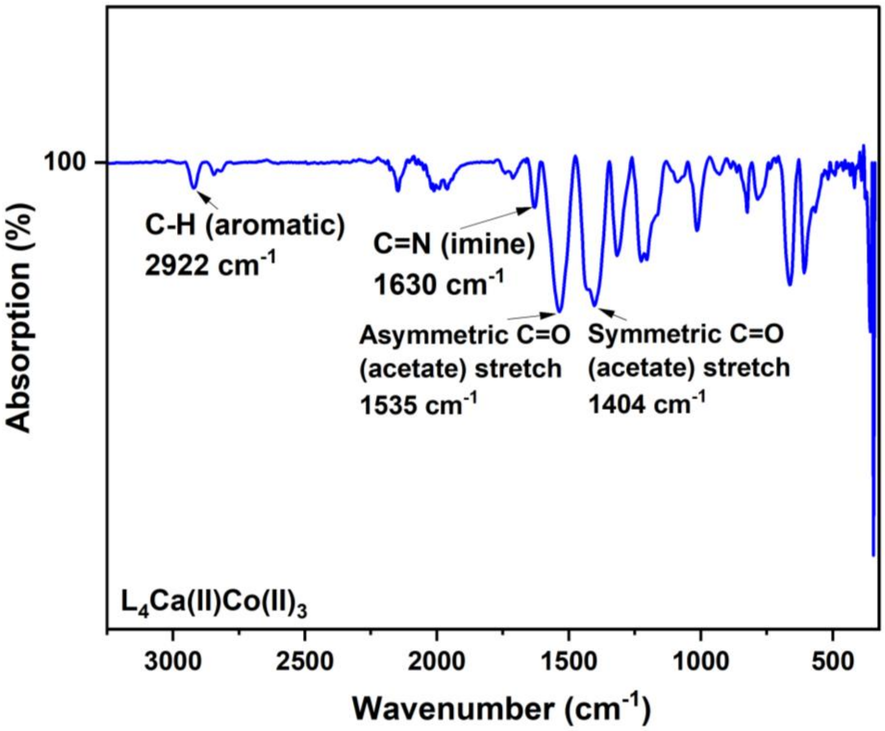

Supplementary Figure 25 IR Spectrum of Catalyst 4.

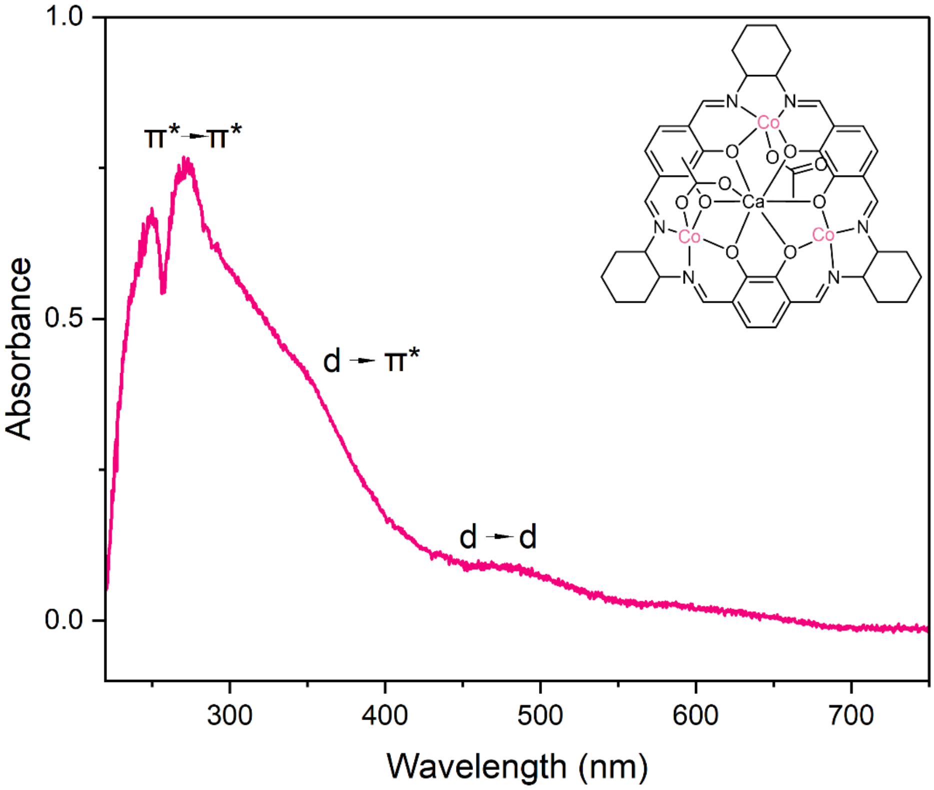

Supplementary Figure 26 UV/Vis Spectrum of Catalyst 4 (0.125 mM in MeCN).

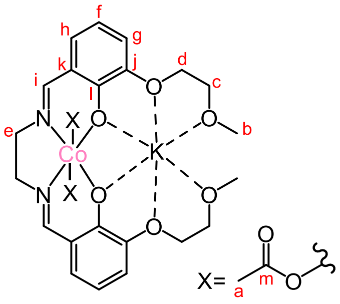

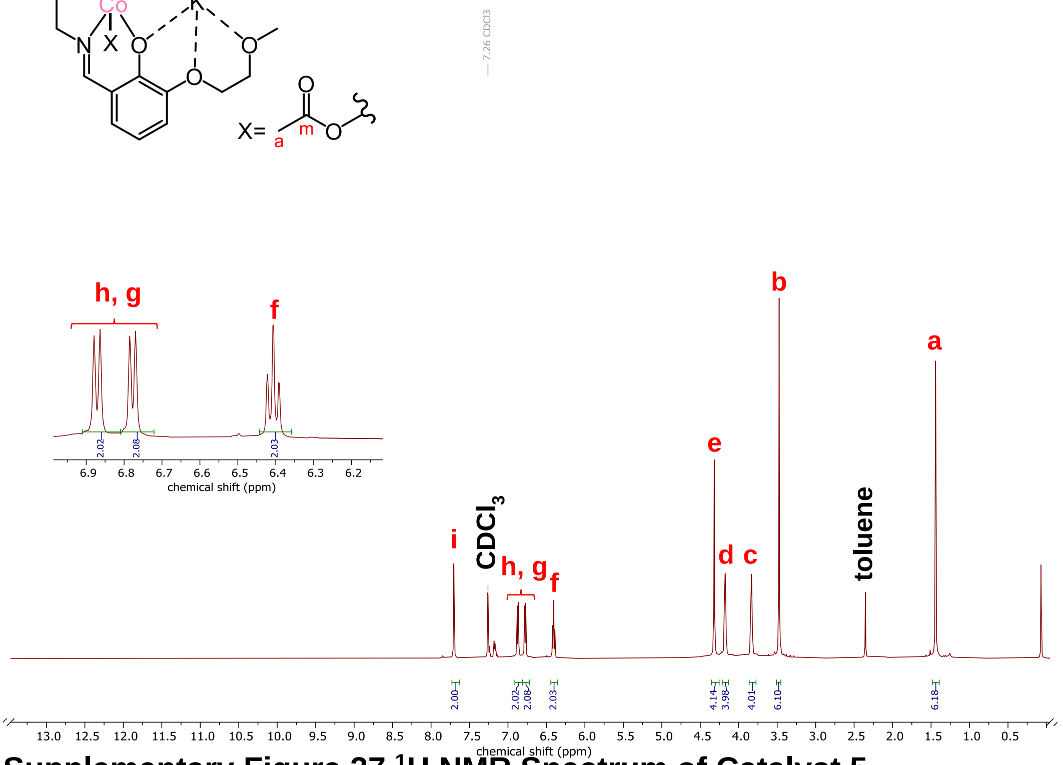

Supplementary Figure 27 ^{1}H NMR Spectrum of Catalyst 5.

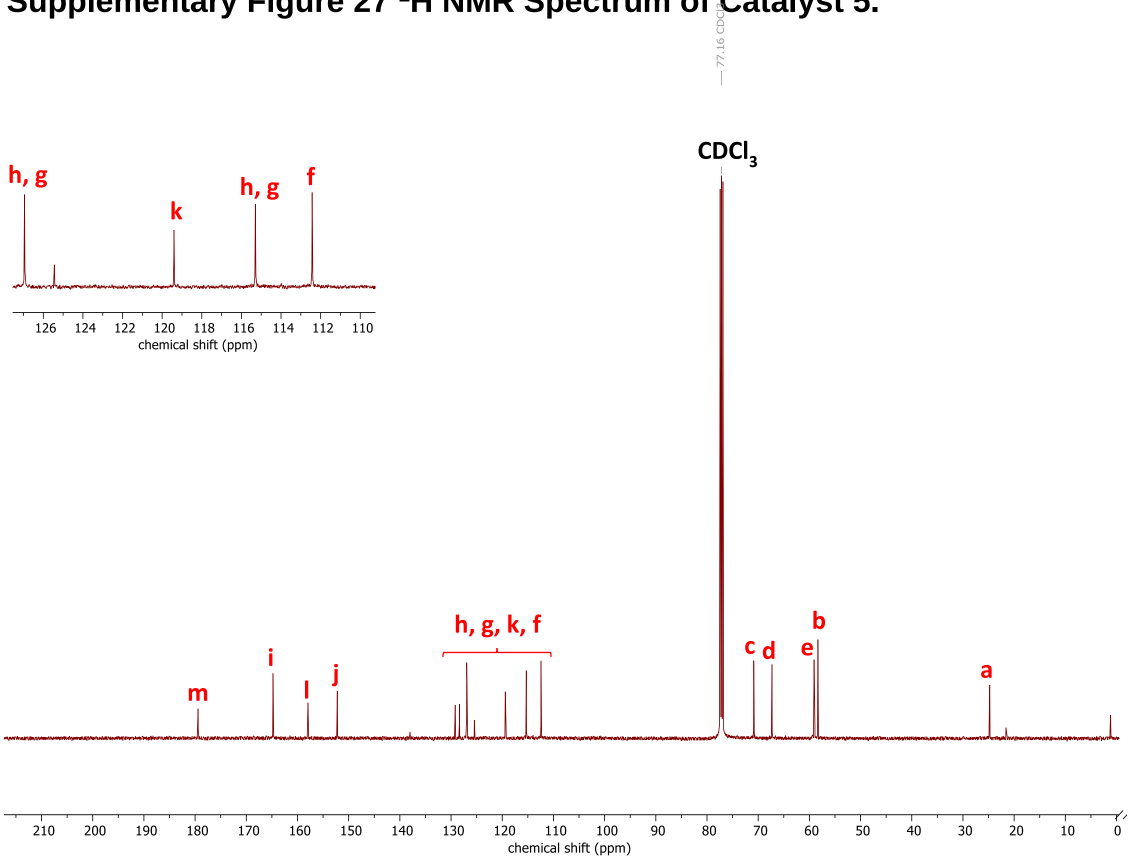

Supplementary Figure 28 $^{13}C\{^{1}H\}$ Spectrum of Catalyst 5.

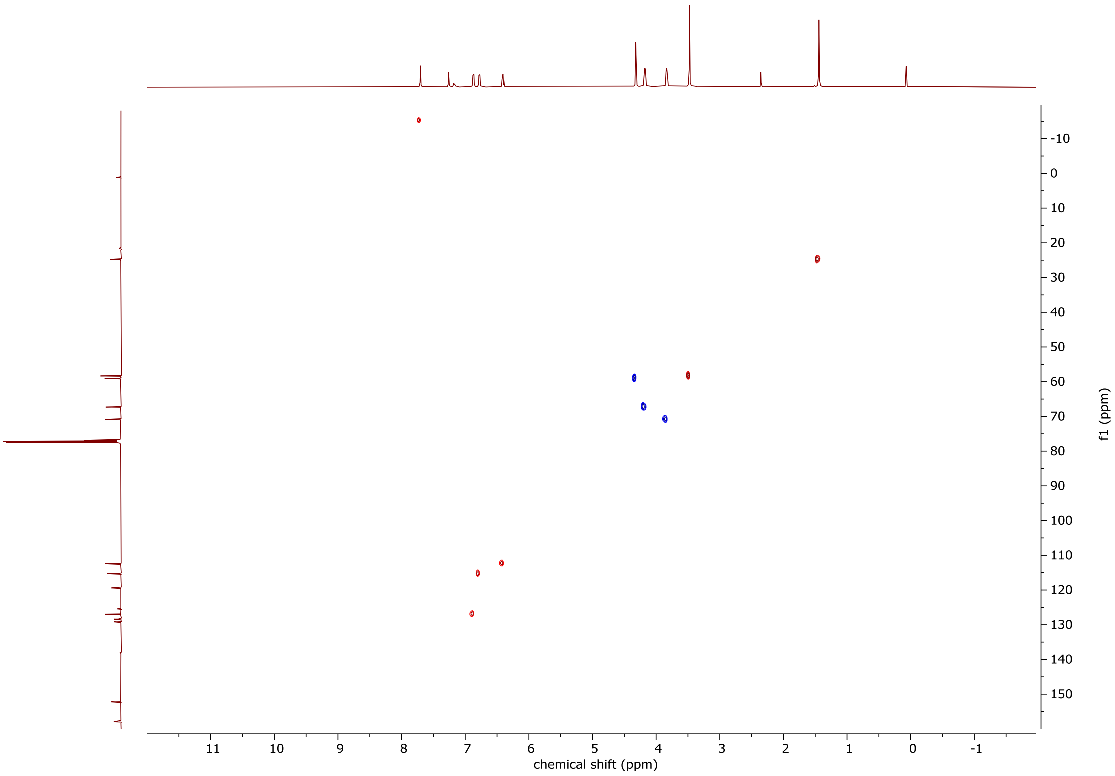

Supplementary Figure 29 2D ^{1}H -^{13}C HSQC Spectrum of Catalyst 5.

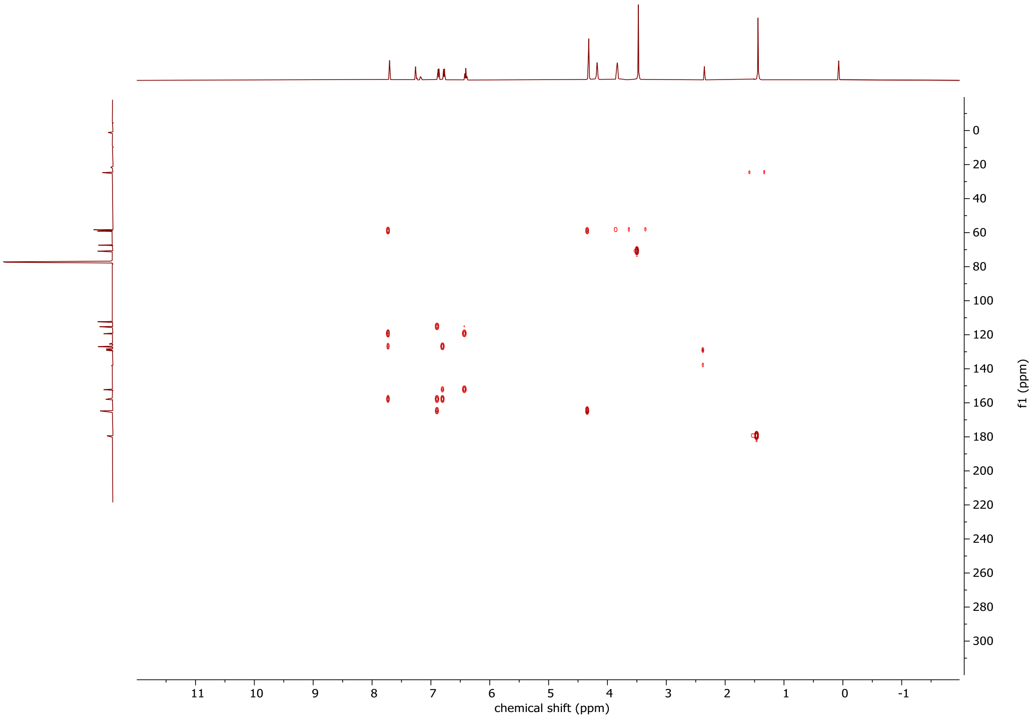

Supplementary Figure 30 2D ^{1}H -^{13}C HMBC Spectrum of Catalyst 5.

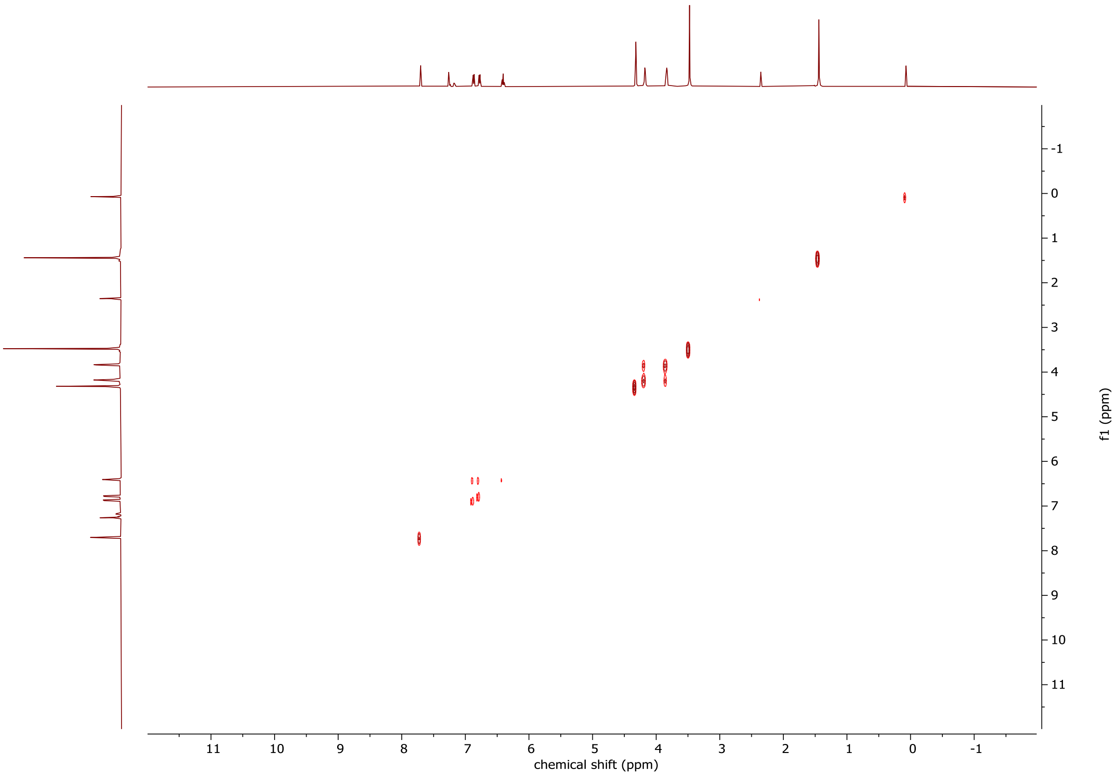

Supplementary Figure 31 2D ^1H -^1H COSY Spectrum of Catalyst 5.

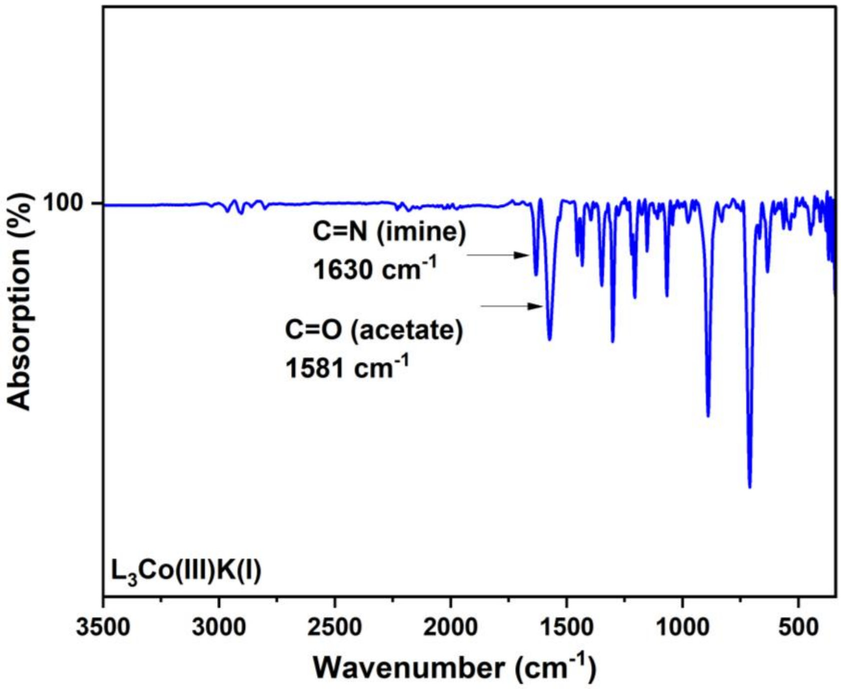

Supplementary Figure 32 IR Spectrum of Catalyst 5.

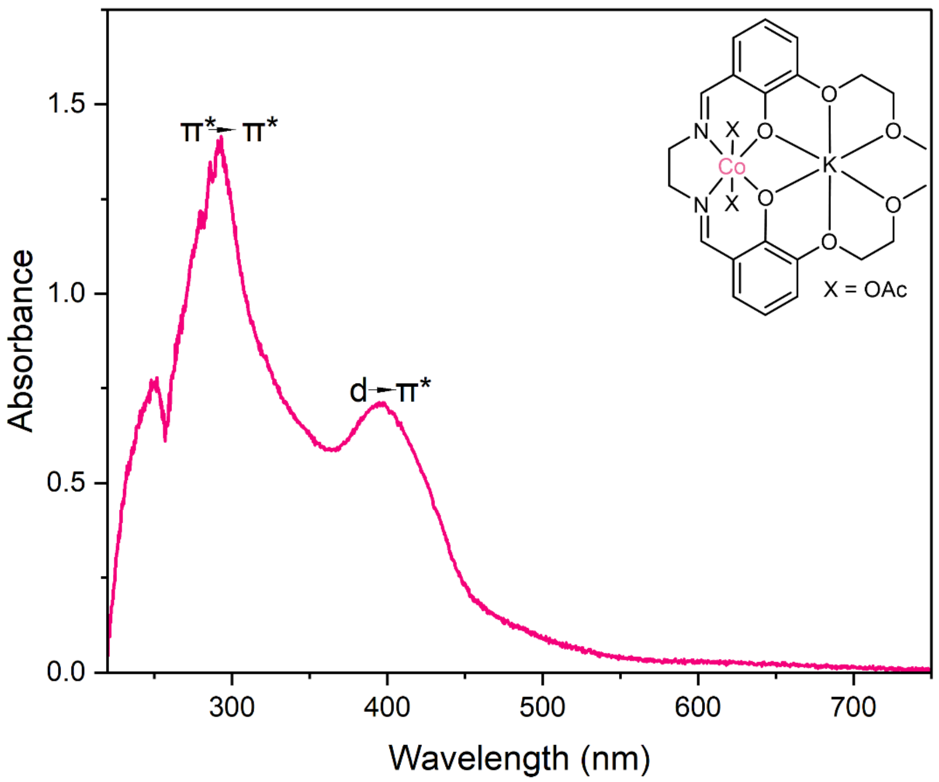

Supplementary Figure 33 UV/Vis Spectrum of Catalyst 5 (0.125 mM in MeCN).

Polymerization and Kinetic Data

Modelling of Rate Laws for Epoxide–CO_2 ROCOP with Catalysts 1-4 (Using COPASI)

The use of the equilibrium constant, K_{eq}, allowed for all the kinetic data to be treated using a common rate law (3), applicable across all pressures, and accounting for the influence of the CO_2 insertion equilibrium. The observation of two regimes of CO_2 pressure dependence ('linear' first-order, and 'plateau' zero-order) was also used to model the data with two further rate laws examining these two regimes ((4) and (5)).

The modelled and experimental rate data for each of these three rate laws is presented in Supplementary Figure 37.

$$\text{rate}=k_p K_{eq}[\text{alkoxide}][CO_2][PO] \quad (3)$$

$$\text{rate in the 'linear' regime} = k_p[\text{cat}]_0[\text{epoxide}][CO_2] \quad (4)$$

$$\text{rate in the 'plateau' regime} = k_p[\text{cat}]_0[\text{epoxide}] \quad (5)$$

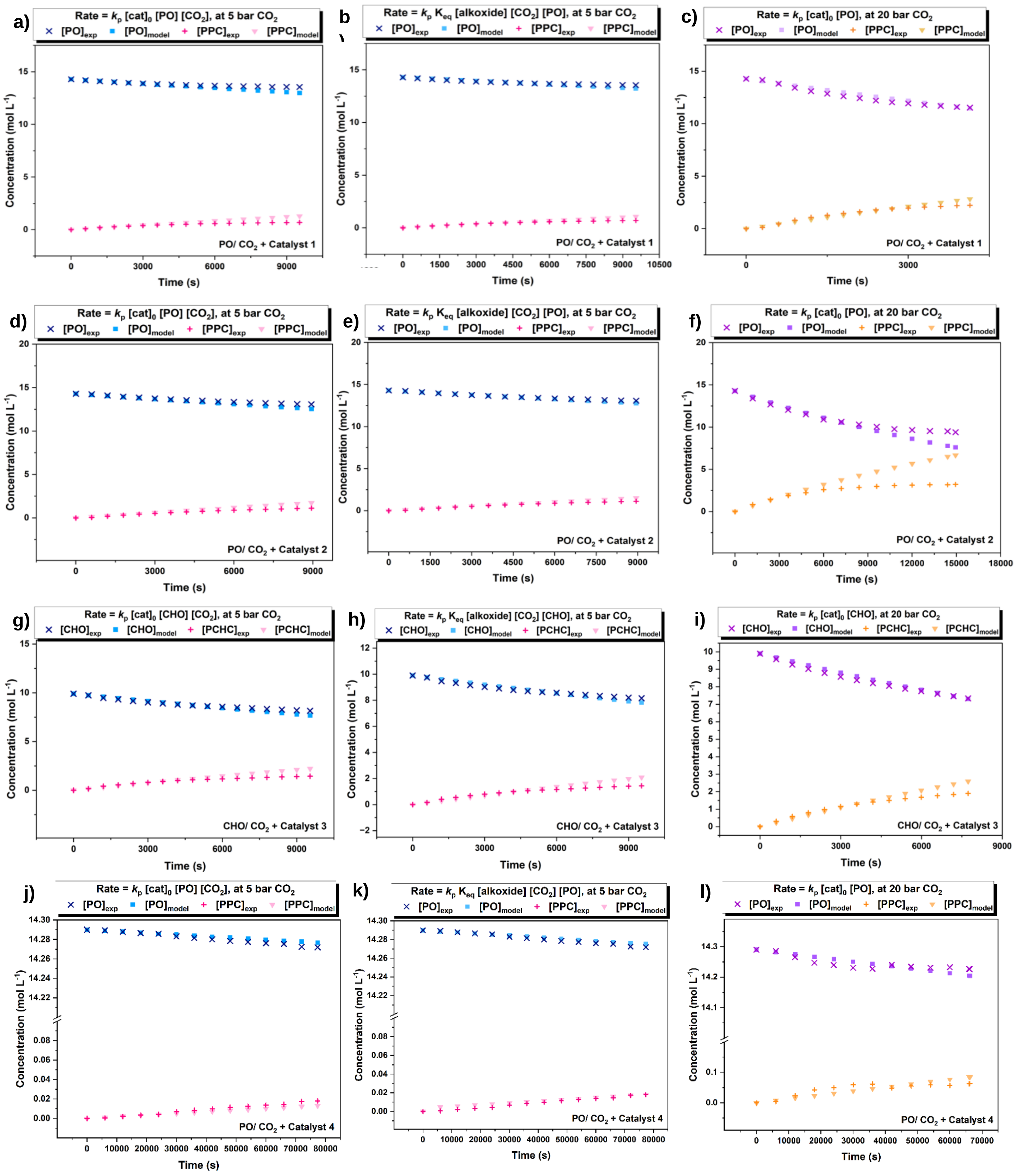

Supplementary Figure 34 Modelling for epoxide–CO_2 ROCOP with catalysts 1-4 (using COPASI). Experimental and modelled data points are shown. For clarity, every tenth datapoint from the datasets in a)-i) are shown, and every hundredth datapoint from the datasets in j)-l) are shown. a), d), g) & j) model the experimental rate law at 5 bar CO_2. b), e), h) & k) model the unified rate law at 5 bar CO_2. c), f), i) & l) model the experimental rate law at 20 bar CO_2.

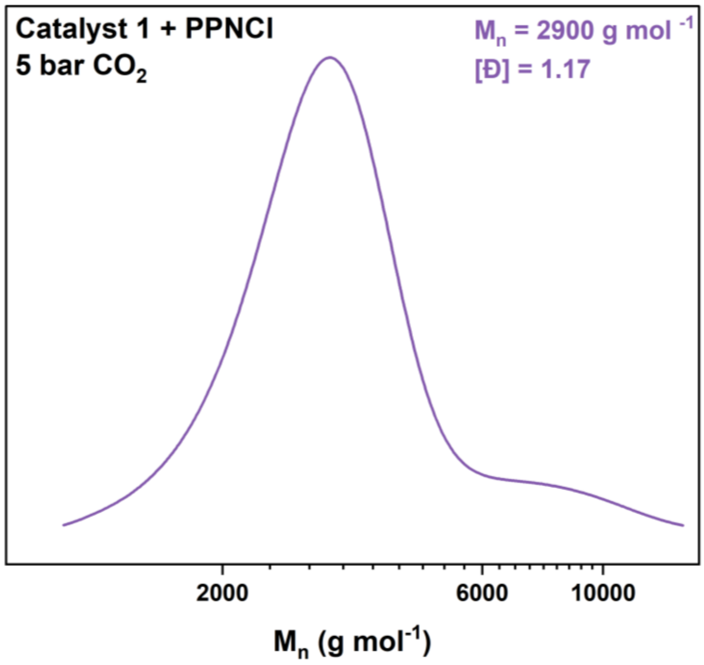

Supplementary Figure 35 Representative GPC trace for PO–CO_2 ROCOP with catalyst 1 + PPNCl at 50 °C, 5 bar CO_2, 1:20:4000:1 catalyst 1:diol:PO:PPNCl.

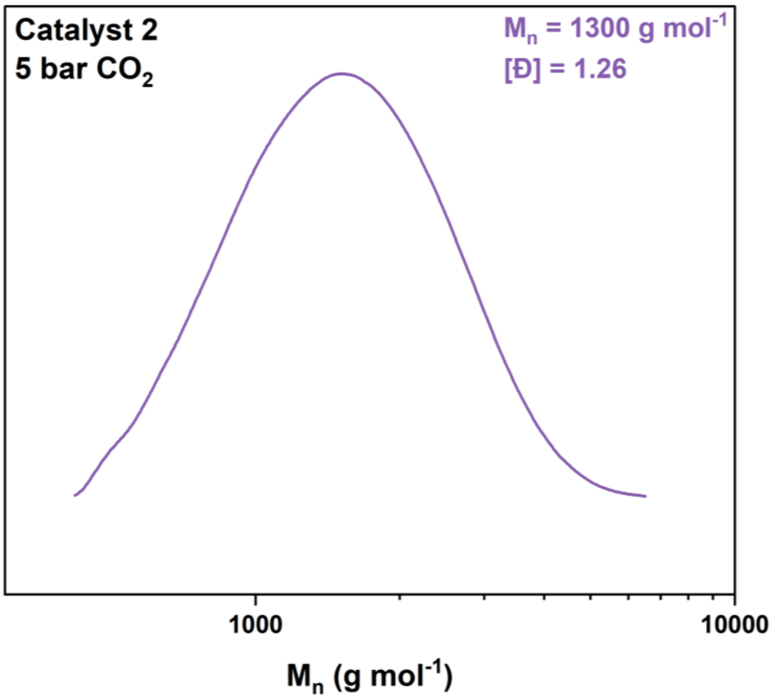

Supplementary Figure 36 Representative GPC trace for PO–CO_2 ROCOP with catalyst 2 at 50 °C, 5 bar CO_2, 1:20:4000 catalyst 2:diol:PO.

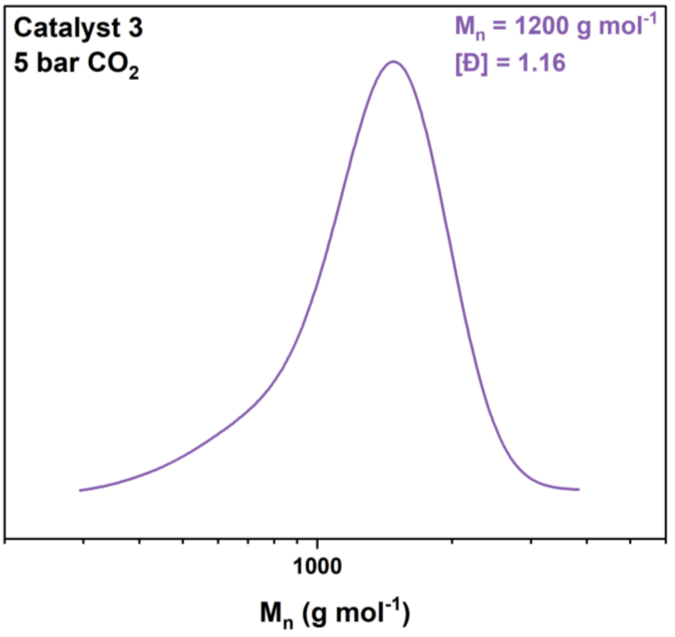

Supplementary Figure 37 Representative GPC trace for CHO–CO_2 ROCOP with catalyst 3 at 50 °C, 5 bar CO_2, 1:20:4000 catalyst 3:diol:PO.

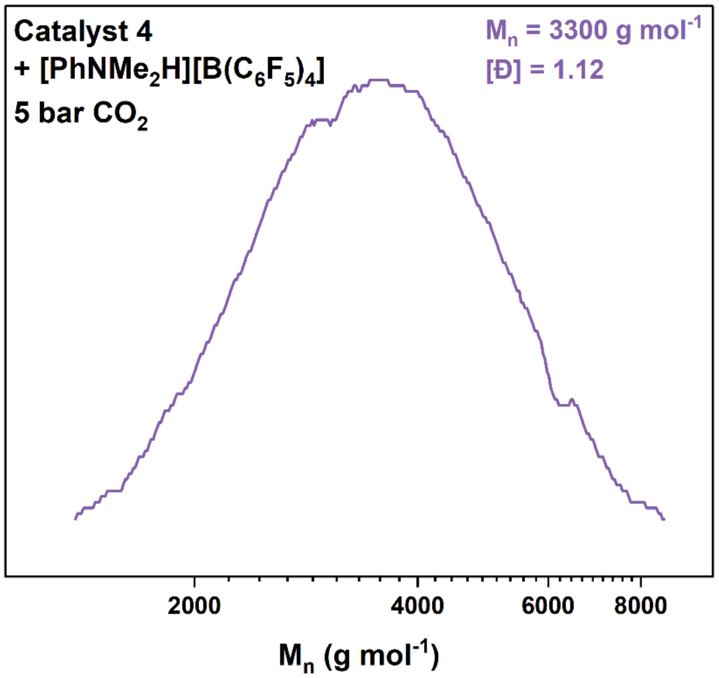

Supplementary Figure 38 Representative GPC trace for PO–CO_2 ROCOP with catalyst 4 + [PhNMe$_2$H][B(C$_6$F$_5$)$_4$] at 50 °C, 5 bar CO_2, 1:20:4000:1 Catalyst 4:diol:PO:[PhNMe$_2$H][B(C$_6$F$_5$)$_4$].

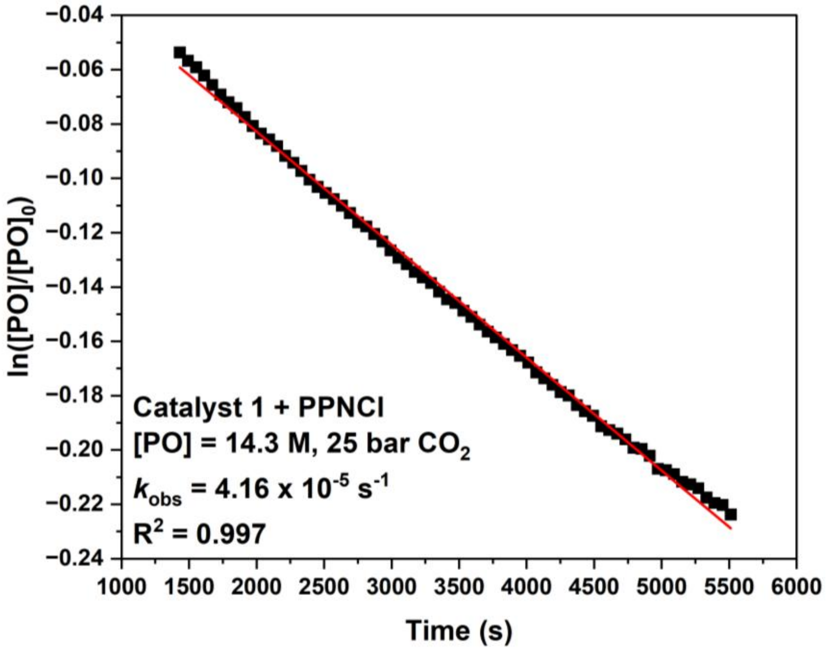

Supplementary Figure 39 Exemplar semilogarithmic plot of ln([PO]/[PO]$_0$) *vs* time for catalyst 1 and a PPNCl co-catalyst.

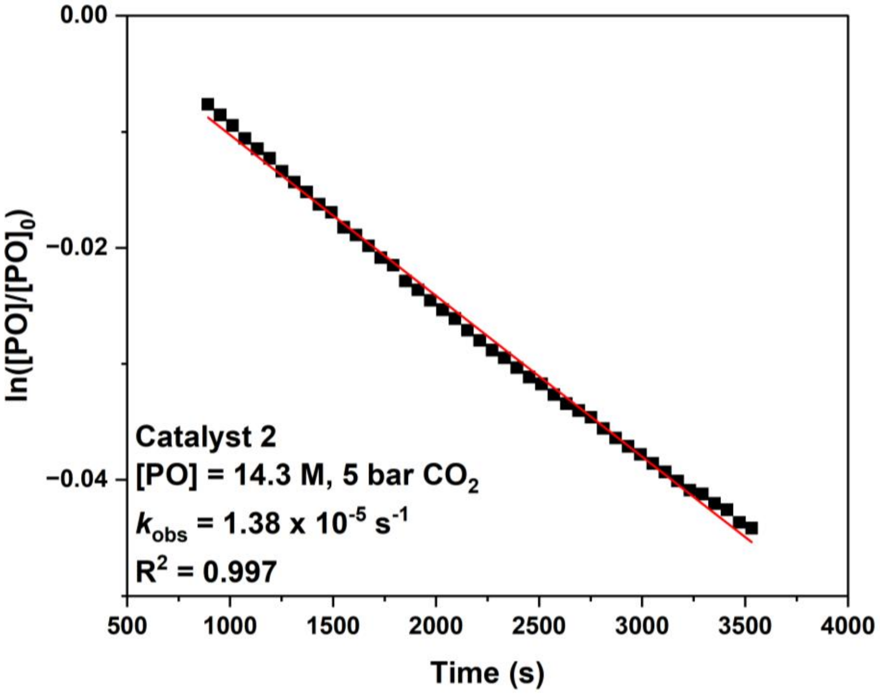

Supplementary Figure 40 Exemplar semilogarithmic plot of ln([PO]/[PO]$_0$) vs time for catalyst 2.

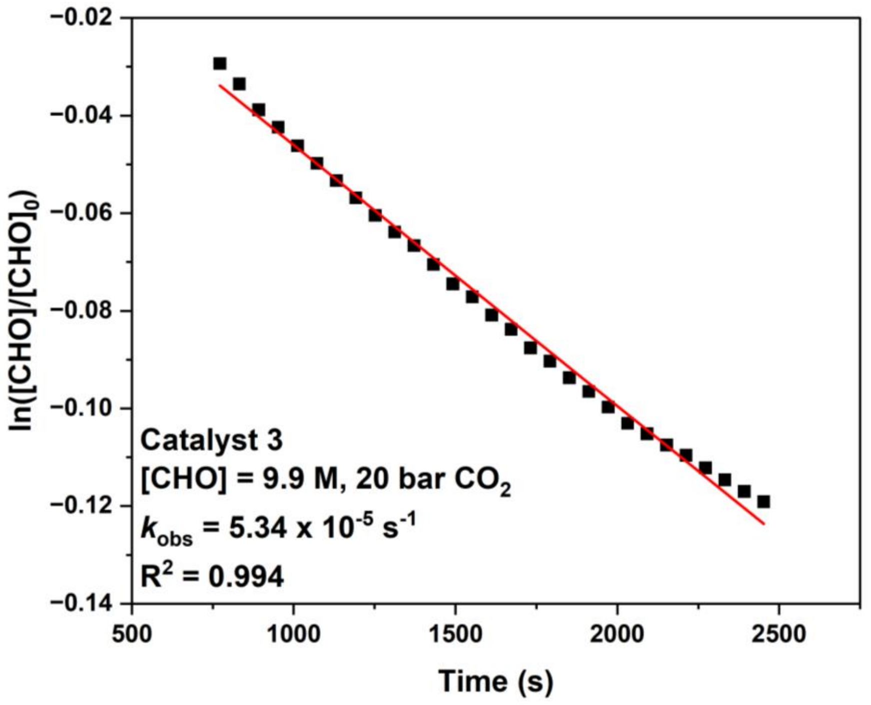

Supplementary Figure 41 Exemplar semilogarithmic plot of ln([CHO]/[CHO]$_0$) vs time for catalyst 3.

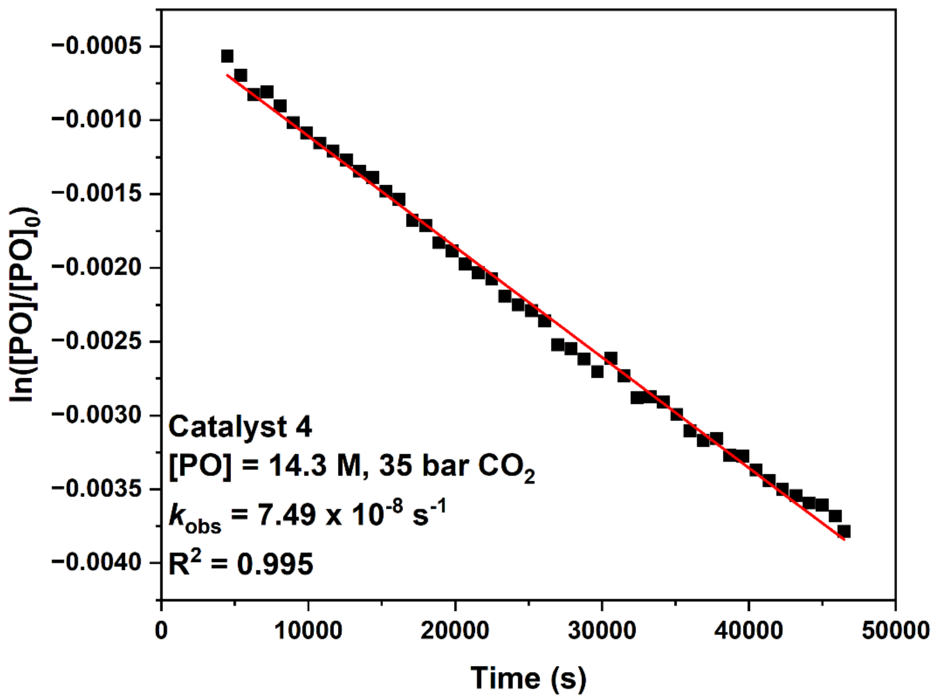

Supplementary Figure 42 Exemplar semilogarithmic plot of ln([PO]/[PO]$_0$) vs time with catalyst 4 and a [$PhNMe_2H$][$B(C_6F_5)_4$] co-catalyst.

Supplementary Table 3 Polymerization data for PO–CO_2 ROCOP with catalyst 1 and a PPNCl co-catalyst.

#	$P(CO_2)$ (bar)	TON [a]	TOF (h^{-1})[b]	PPC Select. (%)[c]	CO_2 Select. (%)[d]	k_{obs} / 10^{-5} (s^{-1})[e]
1	5	424 ± 25	125 ± 2	78 ± 1	> 99	0.90 ± 0.04
2	10	709 ± 14	342 ± 68	75 ± 4	> 99	1.75 ± 0.15
3	12	722 ± 31	465 ± 45	88 ± 3	> 99	2.68 ± 0.19
4	14	896 ± 22	592 ± 29	88 ± 1	> 99	3.63 ± 0.14
5	15	1257 ± 77	535 ± 38	83 ± 1	> 99	3.95 ± 0.22
6	20	1029 ± 61	715 ± 42	86 ± 2	> 99	5.38 ± 0.03
7	25	876 ± 86	601 ± 100	96 ± 1	> 99	4.90 ± 0.52
8	30	1077 ± 140	668 ± 34	94 ± 1	> 99	6.05 ± 0.75
9	35	1021 ± 33	663 ± 68	95 ± 1	> 99	5.27 ± 0.61

Polymerization conditions: 50 ℃, catalyst (0.025 mol%, 3.6 mM), PPNCl co-catalyst (0.025 mol%), PO (6 mL, 14.3 M), *trans*-1,2-cyclohexanediol (0.5 mol%, 71 mM) under fixed CO_2 pressure. [a]TON was calculated from ^{1}H NMR spectroscopy of PPC (4.92 ppm, 1H), PC (4.77 ppm, 1H) and poly(propylene) oxide (3.46-3.64 ppm, 3H) against mesitylene (0.25 mol%, 36 mM) as an internal standard. [b]Calculated with TOF = TON/time. [c]Polymer selectivity was determined by dividing the sum of ^{1}H NMR integrals for polycarbonate and polyether against the sum of integrals for polycarbonate, cyclic carbonate and polyether. [d]CO_2 selectivity was determined by dividing the sum of ^{1}H NMR integrals for polycarbonate and cyclic carbonate against the sum of integrals for polycarbonate, cyclic carbonate and polyether. [e]k_{obs} determined as the gradient of the plot of $\ln[PO]_t/[PO]_0$ *vs.* time.

Supplementary Table 4 Polymerization data for PO–CO_2 ROCOP with catalyst 2.

#	P(CO_2) (bar)	TON [a]	TOF (h^{-1})[b]	PPC Select. (%)[c]	CO_2 Select. (%)[d]	k_{obs} / 10^{-5} (s^{-1})[e]
1	2	413 ± 47	114 ± 1	63 ± 4	98 ± 1	0.49 ± 0.01
2	5	654 ± 195	277 ± 55	84 ± 5	>99	1.46 ± 0.05
3	7	845 ± 49	242 ± 7	96	>99	1.74 ± 0.15
4	10	638 ± 70	334 ± 8	95 ± 1	>99	2.55 ± 0.03
5	12	754 ± 60	304 ± 31	93	>99	2.82 ± 0.01
6	14	751 ± 59	387 ± 34	94	>99	3.54 ± 0.01
7	15	822 ± 107	476 ± 14	98	>99	4.21 ± 0.01
8	20	1203 ± 112	448 ± 71	98 ± 1	>99	4.04 ± 0.13
9	25	1073 ± 151	449 ± 26	97 ± 1	>99	3.97 ± 0.01

Polymerization conditions: 50 ℃, catalyst (0.025 mol%, 3.6 mM), PO (6 mL, 14.3 M), trans-1,2-cyclohexanediol (0.5 mol%, 71 mM) at fixed CO_2 pressure. [a]TON was calculated from ^{1}H NMR spectroscopy of PPC (4.92 ppm, 1H), PC (4.77 ppm, 1H) and poly(propylene) oxide (3.46-3.64 ppm, 3H) against mesitylene (0.25 mol%, 36 mM) as an internal standard. [b]Calculated with TOF = TON/time. [c]Polymer selectivity was determined by dividing the sum of ^{1}H NMR integrals for polycarbonate and polyether by the sum of integrals for polycarbonate, cyclic carbonate and polyether. [d]CO_2 selectivity was determined by dividing the sum of ^{1}H NMR integrals for polycarbonate and cyclic carbonate by the sum of integrals for polycarbonate, cyclic carbonate and polyether. [e]kobs determined as the gradient of the plot of $\ln[PO]_t/[PO]_0$ *vs.* time.

Supplementary Table 5 Polymerization data for CHO–CO_2 ROCOP with catalyst 3.

#	P(CO_2) (bar)	TON [a]	TOF (h^{-1})[b]	PPC Select. (%)[c]	CO_2 Select. (%)[d]	k_{obs} / 10^{-5} (s^{-1})[e]
1	2	707 ± 47	216 ± 37	> 99	> 99	1.31 ± 0.01
2	4	739 ± 22	262 ± 12	> 99	> 99	1.81 ± 0.08
3	5	679 ± 24	249 ± 11	> 99	> 99	2.64 ± 0.02
4	7	1005 ± 9	349 ± 43	> 99	> 99	3.72 ± 0.23
5	10	990 ± 45	432 ± 31	> 99	> 99	4.00 ± 0.08
6	15	1105 ± 22	439 ± 24	> 99	> 99	4.41 ± 0.19
7	20	980 ± 49	435 ± 30	> 99	> 99	4.64 ± 0.51
8	25	1210 ± 133	535 ± 19	> 99	> 99	4.44 ± 0.22
9	30	1197 ± 16	473 ± 1	> 99	> 99	4.31 ± 0.003

Polymerization conditions: 50 ℃, catalyst (0.025 mol%, 2.5 mM), CHO (6 mL, 9.9 M), *trans*-1,2-cyclohexanediol (0.5 mol%, 71 mM) at fixed CO_2 pressure. [a]TON was calculated from ^{1}H NMR spectroscopy of PCHC (4.59 ppm, 2H) against mesitylene (0.25 mol%, 36 mM) as an internal standard. [b]Calculated with TOF = TON/time. [c]Polymer selectivity was determined by dividing the sum of ^{1}H NMR integrals for polycarbonate and polyether against the sum of integrals for polycarbonate, cyclic carbonate and polyether. [d]CO_2 selectivity was determined by dividing the sum of ^{1}H NMR integrals for polycarbonate and cyclic carbonate against the sum of integrals for polycarbonate, cyclic carbonate and polyether. [e]k_{obs} determined as the gradient of the plot of $\ln[CHO]_t/[CHO]_0$ *vs.* time.

Supplementary Table 6 Polymerization data for PO–CO_2 ROCOP with catalyst 4 with a [$PhNMe_2H$][$B(C_6F_5)_4$] co-catalyst.

#	$P(CO_2)$ (bar)	TON [a]	TOF (h^{-1})[b]	PPC Select. (%)[c]	CO_2 Select. (%)[d]	k_{obs} / 10^{-8} (s^{-1})[e]
1	5	79 ± 4	5 ± 0.4	4 ± 1	93 ± 3	1.11 ± 0.02
2	7	88 ± 1	6 ± 0.6	5 ± 1	89 ± 1	2.02 ± 0.61
3	10	84 ± 12	5 ± 0.4	6 ± 1	96 ± 2	3.14 ± 0.23
4	15	78 ± 6	5 ± 0.06	11	95 ± 1	4.14 ± 0.61
5	16	85 ± 9	6 ± 0.4	15	91 ± 3	6.22 ± 0.21
6	20	109 ± 8	6 ± 0.04	17	88 ± 1	7.82 ± 0.86
7	25	74 ± 11	4 ± 0.2	19 ± 1	89 ± 1	9.54 ± 0.40
8	35	99 ± 3	6 ± 1	17 ± 1	92 ± 3	7.36 ± 0.10

Polymerization conditions: 50 ℃, catalyst (0.025 mol%, 3.6 mM), [$PhNMe_2H$][$B(C_6F_5)_4$] co-catalyst (0.025 mol%), PO (6 mL, 14.3 M), *trans*-1,2-cyclohexanediol (0.5 mol%, 71 mM) at fixed CO_2 pressure. [a]TON was calculated from ^{1}H NMR spectroscopy of PPC (4.92 ppm, 1H), PC (4.77 ppm, 1H) and poly(propylene) oxide (3.46-3.64 ppm, 3H) against mesitylene (0.25 mol%, 36 mM) as an internal standard. [b]Calculated with TOF = TON/time. [c]Polymer selectivity was determined by dividing the sum of ^{1}H NMR integrals for polycarbonate and polyether against the sum of integrals for polycarbonate, cyclic carbonate and polyether. [d]CO_2 selectivity was determined by dividing the sum of ^{1}H NMR integrals for polycarbonate and cyclic carbonate against the sum of integrals for polycarbonate, cyclic carbonate and polyether. [e]k_{obs} determined as the gradient of the plot of $\ln[PO]_t/[PO]_0$ *vs.* time.

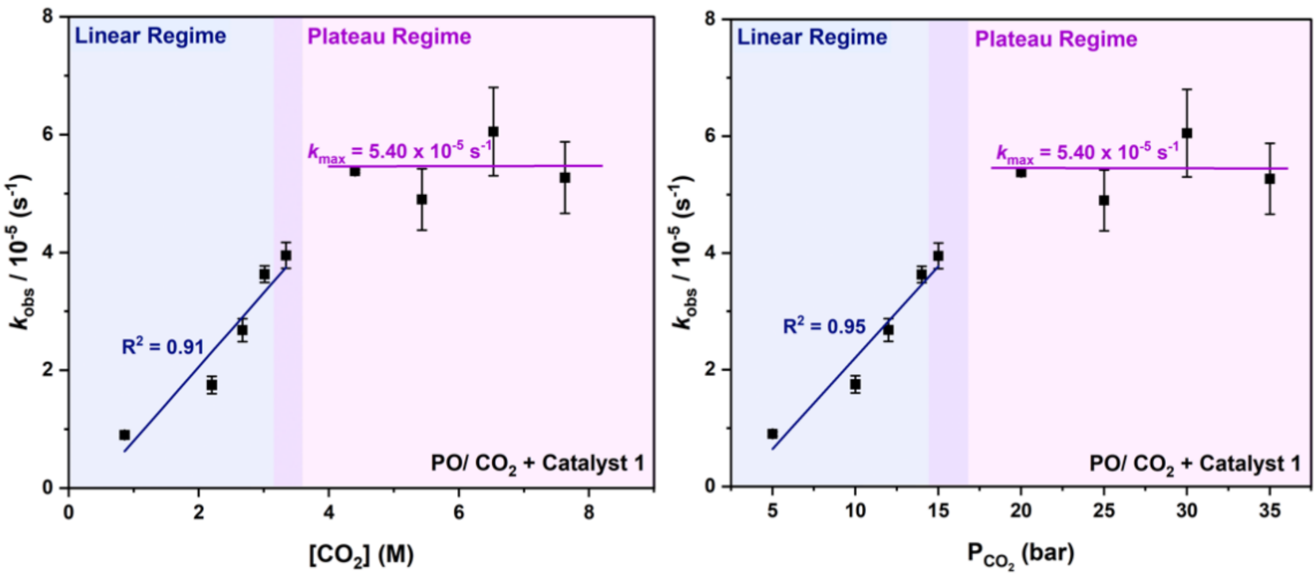

Supplementary Figure 43 Plots of k_{obs} *vs*. [CO_2] and k_{obs} *vs*. P(CO_2) for PO–CO_2 ROCOP with catalyst 1 with PPNCl co-catalyst. k_{obs} values were determined as the average of n=2 independent runs, with errors indicated as ± the standard error from the mean, typically falling ± 10%.

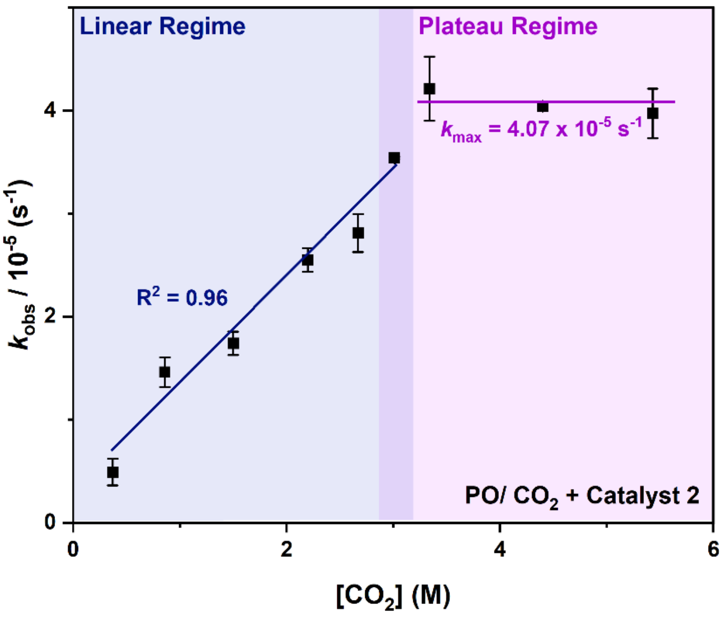

Supplementary Figure 44 Plots of k_{obs} *vs*. [CO_2] for PO–CO_2 ROCOP with catalyst 2. k_{obs} values were determined as the average of n=2 independent runs, with errors indicated as ± the standard error from the mean, typically falling ± 10%.

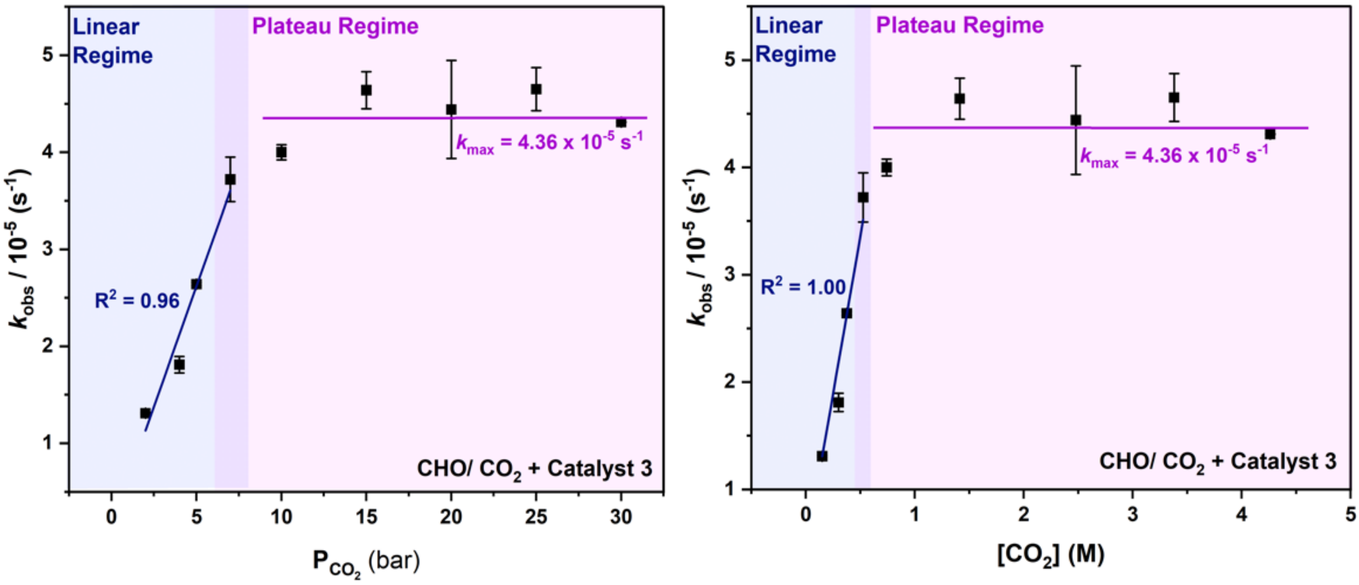

Supplementary Figure 45 Plots of k_{obs} *vs*. [CO_2] and k_{obs} *vs*. P(CO_2) for CHO–CO_2 ROCOP with catalyst 3. k_{obs} values were determined as the average of n=2 independent runs, with errors indicated as ± the standard error from the mean, typically falling ± 10%.

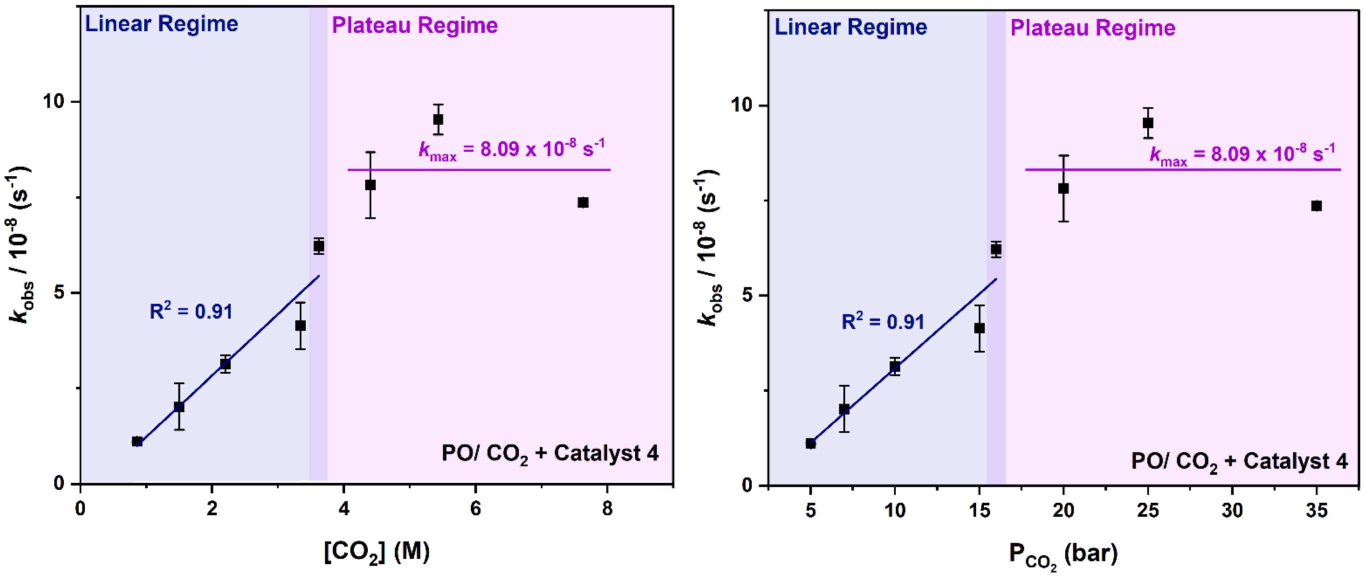

Supplementary Figure 46 Plots of k_{obs} *vs*. [CO_2] and k_{obs} *vs*. P(CO_2) for PO–CO_2 ROCOP with catalyst 4 with a [$PhNMe_2H$][$B(C_6F_5)_4$] co-catalyst. k_{obs} values were determined as the average of n=2 independent runs, with errors indicated as ± the standard error from the mean, typically falling ± 10%.

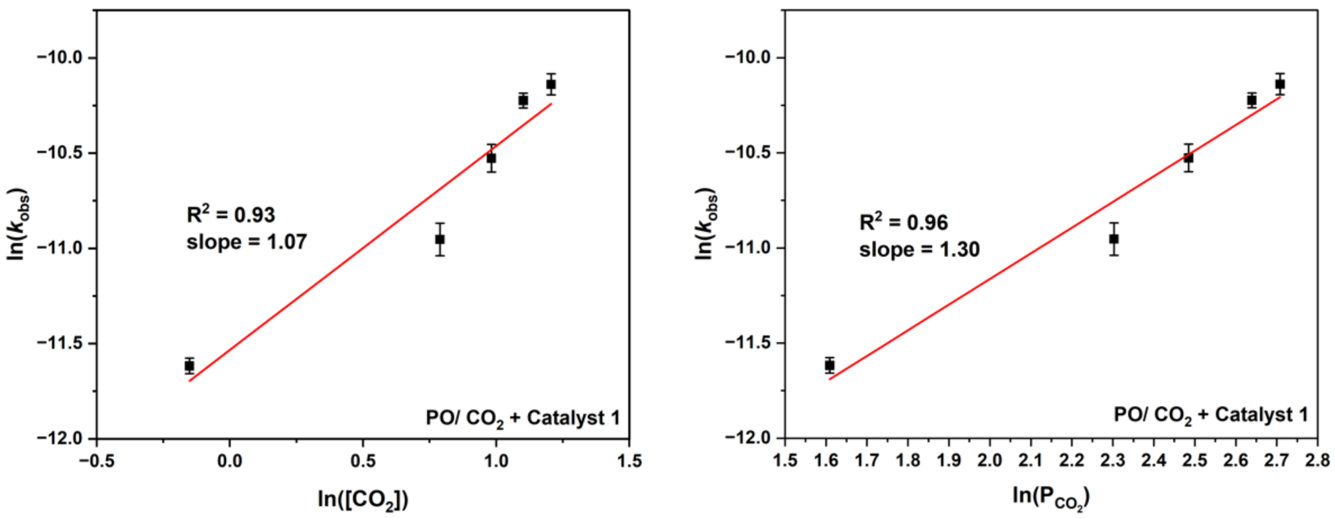

Supplementary Figure 47 Plots of ln(k_{obs}) *vs*. ln([CO_2]) and ln(k_{obs}) *vs*. ln(P(CO_2)) for PO–CO_2 ROCOP with catalyst 1 and a PPNCl co-catalyst. All ln(k_{obs}) values were determined from k_{obs} values obtained as the average of n=2 independent runs, with errors indicated as ± the standard error from the mean.

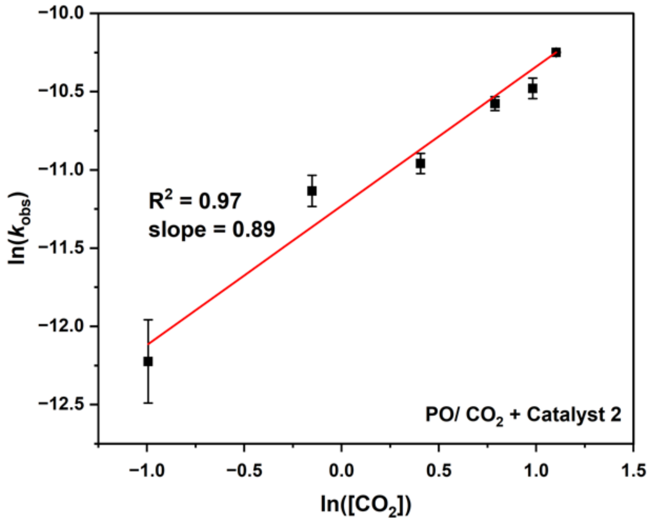

Supplementary Figure 48 Plot of ln(k_{obs}) *vs*. ln([CO_2]) for PO–CO_2 ROCOP with catalyst 2. All ln(k_{obs}) values were determined from k_{obs} values obtained as the average of n=2 independent runs, with errors indicated as ± the standard error from the mean.

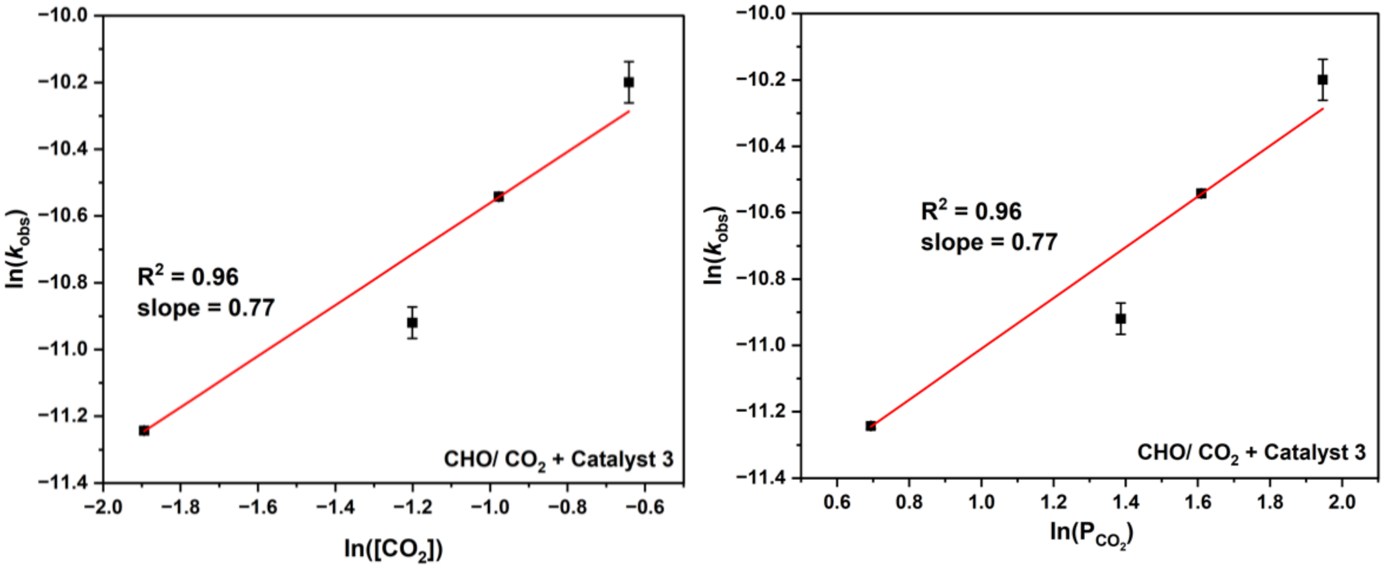

Supplementary Figure 49 Plots of ln(k_{obs}) *vs*. ln([CO_2]) and ln(k_{obs}) *vs*. ln(P(CO_2)) for CHO–CO_2 ROCOP with catalyst 3. All ln(k_{obs}) values were determined from k_{obs} values obtained as the average of n=2 independent runs, with errors indicated as ± the standard error from the mean.

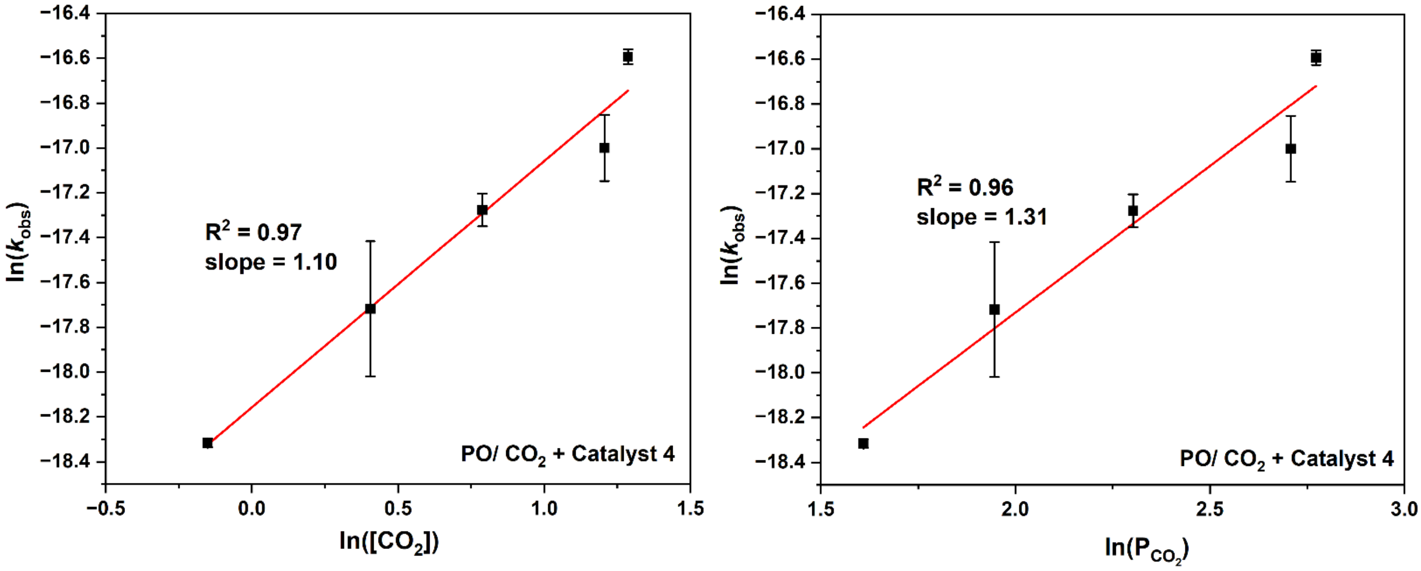

Supplementary Figure 50 Plots of ln(k_{obs}) *vs*. ln([CO_2]) and ln(k_{obs}) *vs*. ln(P(CO_2)) for PO–CO_2 ROCOP with catalyst 4 and a [$PhNMe_2H$][$B(C_6F_5)_4$] co-catalyst. All ln(k_{obs}) values were determined from k_{obs} values obtained as the average of n=2 independent runs, with errors indicated as ± the standard error from the mean.

Calculating the CO_2 insertion Equilibrium Constant, K_{eq}

Using the same method as reported in reference [3], the average rate constant observed in the 'plateau' regime corresponds to the maximum observable rate, at any pressure, for those conditions (temperature, catalyst loading). Assuming that in the plateau regime, the carbon dioxide equilibrium lies completely towards the carbonate intermediate, then $[\text{catalyst}]_0$ = [carbonate]. Given that the catalyst can only be speciated as an alkoxide or carbonate intermediate, the concentration of carbonate intermediate at a given pressure, $[\text{carbonate}]_p$, is determined from the rate observed at that pressure, rate_p, the maximum observed rate, rate_{max}, and the maximum carbonate concentration, $[\text{carbonate}]_{max}$ (which is equal to the initial catalyst concentration, $[\text{cat}]_0$), according the below expression (1):

$$\frac{\text{rate}_p}{\text{rate}_{max}} \times [\text{carbonate}]_{max} = [\text{carbonate}]_p \qquad (1)$$

Thus, there is a linear relationship between [carbonate] and $[CO_2]$ in the 'linear' pressure regime, which reaches a constant maximum in the 'plateau' regime. The $[\text{alkoxide}]_p$ can be determined in the 'linear' regime, assuming no catalyst side or decomposition reactions, since the sum of alkoxide and carbonate concentrations must be the same as the catalyst concentration ($[\text{cat}]_0$).

The CO_2 insertion equilibrium constant can then be determined for each pressure in the linear regime, according to (5) with $[\text{carbonate}]_p$ now known for each pressure. The final K_{eq} is determined as the average K_{eq} for each $[CO_2]$ and $[\text{carbonate}]_p$ in the 'linear' regime, with the error calculated from the error in the range of these values in the linear regime (Supplementary Tables 11-15).

$$K_{eq} = \frac{[\text{carbonate}]}{[\text{alkoxide}][CO_2]} \qquad (2)$$

Calculation of K_{eq} for catalysts was completed using this method for every catalyst.[3]

Supplementary Table 7 Calculation of K_{eq} for PO–CO_2 ROCOP with catalyst 1 and a PPNCl co-catalyst.

Order in CO_2	CO_2 Pressure (bar)	$[CO_2]$ (M)[a]	k_{obs}/ 10^{-5} (s^{-1})[b]	[carbonate] / 10^{-3} (M)[c]	[alkoxide] / 10^{-3} (M)[d]	K_{eq} (M^{-1})[e]
First order	5	0.86	0.90	0.60	2.97	0.23
First order	10	2.20	1.75	1.16	2.41	0.22
First order	12	2.67	2.68	1.77	1.80	0.37
First order	14	3.01	3.63	2.40	1.17	0.68
Change in order	15	N/A	3.95	2.61	N/A	N/A
Zero order	20	N/A	5.38	3.56	N/A	N/A
Zero order	25	N/A	4.90	3.24	N/A	N/A
Zero order	30	N/A	6.05	4.00	N/A	N/A
Zero order	35	N/A	5.27	3.48	N/A	N/A

[a]Calculated from data supplied by Tassaing and co-workers[14]. [b]Averages of n = 2 runs, with an error of $\Delta x = \frac{\sigma}{\sqrt{n}}$. [c]Calculated using $\frac{rate_p}{rate_{max}} \times [carbonate]_{max} = [carbonate]_p$ with average k_{obs} values and k_{max}= 5.40 x 10^{-5} s^{-1} (the average k_{obs} in the zero order regime) [d] Determined using $[cat]_0$ – [carbonate] = [alkoxide] for the 'linear' regime, where $[cat]_0$ = 3.57 x 10^{-3} s^{-1}. [e] Calculated from $K_{eq} = \frac{[carbonate]}{[alkoxide][CO_2]}$.

Average K_{eq} = 0.38 ± 0.08 M^{-1} (first-order regime)

Supplementary Table 8 Calculation of K_{eq} for PO–CO_2 ROCOP with catalyst 2.

Order in CO_2	CO_2 Pressure (bar)	$[CO_2]$ (M)[a]	k_{obs}/ 10^{-5} (s^{-1})[b]	[carbonate] / 10^{-3} (M)[c]	[alkoxide] / 10^{-3} (M)[d]	K_{eq} (M^{-1})[e]
First order	2	0.37	0.49	0.43	3.14	0.37
First order	5	0.86	1.46	1.28	2.29	0.65
First order	7	1.50	1.74	1.52	2.05	0.50
First order	10	2.20	2.55	2.23	1.34	0.76
First order	12	2.67	2.82	2.47	1.10	0.84
Change in order	14	N/A	3.54	3.10	N/A	N/A
Zero order	15	N/A	4.21	3.69	N/A	N/A
Zero order	20	N/A	4.04	3.54	N/A	N/A
Zero order	25	N/A	3.97	3.48	N/A	N/A

[a]Calculated from data supplied by Tassaing and co-workers[14]. [b]Averages of n = 2 runs, with an error of $\Delta x = \frac{\sigma}{\sqrt{n}}$. [c]Calculated using $\frac{rate_p}{rate_{max}} \times [carbonate]_{max} = [carbonate]_p$, with average k_{obs} values and k_{max}= 4.07 x 10^{-5} s^{-1} (the average k_{obs} in the zero order regime) [d] Determined using $[cat]_0$ – [carbonate] = [alkoxide] for the 'linear' regime, where $[cat]_0$ = 3.57 x 10^{-3} s^{-1}. [e] Calculated from $K_{eq} = \frac{[carbonate]}{[alkoxide][CO_2]}$.

Average K_{eq} = 0.62 ± 0.09 M^{-1} (first-order regime).

Supplementary Table 9 Calculation of K_{eq} for CHO–CO_2 ROCOP with catalyst 3.

Order in CO_2	CO_2 Pressure (bar)	[CO_2] (M)[a]	k_{obs} / 10^{-5} (s^{-1})[b]	[carbonate] / 10^{-3} (M)[c]	[alkoxide] / 10^{-3} (M)[d]	K_{eq} (M^{-1})[e]
First order	2	0.15	1.31	0.75	1.75	2.86
First order	4	0.30	1.81	1.04	1.46	2.36
First order	5	0.38	2.64	1.51	0.99	4.08
Change in order	7	N/A	3.72	2.13	N/A	N/A
Zero order	10	N/A	4.00	2.29	N/A	N/A
Zero order	15	N/A	4.41	2.53	N/A	N/A
Zero order	20	N/A	4.64	2.66	N/A	N/A
Zero order	25	N/A	4.44	2.55	N/A	N/A
Zero order	30	N/A	4.31	2.47	N/A	N/A

[a]Calculated from data supplied by Tassaing and co-workers[14]. [b]Averages of n = 2 runs, with an error of $\Delta x = \frac{\sigma}{\sqrt{n}}$. [c]Calculated using $\frac{rate_p}{rate_{max}} \times [carbonate]_{max} = [carbonate]_p$, , with average k_{obs} values and k_{max}= 4.36 x 10^{-5} s^{-1} (the average k_{obs} in the zero order regime) [d] Determined using $[cat]_0$ – [carbonate] = [alkoxide] for the 'linear' regime, where $[cat]_0$ = 3.57 x 10^{-3} s^{-1}. [e] Calculated from $K_{eq} = \frac{[carbonate]}{[alkoxide][CO_2]}$.

Average K_{eq} = 3.10 ± 0.26 M^{-1} (first order regime).

Supplementary Table 10 Calculation of K_{eq} for PO–CO_2 ROCOP with catalyst 4 and a $[PhNMe_2H][B(C_6F_5)_4]$ co-catalyst.

Order in CO_2	CO_2 Pressure (bar)	[CO_2] (M)[a]	k_{obs}/ 10^{-8} (s^{-1})[b]	[carbonate] / 10^{-3} (M)[c]	[alkoxide] / 10^{-3} (M)[d]	K_{eq} (M^{-1})[e]
First order	5	0.86	1.11	0.48	3.09	0.18
First order	7	1.50	2.02	0.88	2.69	0.22
First order	10	2.20	3.14	1.36	2.21	0.28
First order	15	3.34	4.14	1.79	1.78	0.30
Change in order	16	N/A	6.22	2.69	N/A	N/A
Zero order	20	N/A	7.82	3.39	N/A	N/A
Zero order	25	N/A	9.54	4.13	N/A	N/A
Zero order	35	N/A	7.36	3.19	N/A	N/A

[a]Calculated from data supplied by Tassaing and co-workers[14]. [b]Averages of n = 2 runs, with an error of $\Delta x = \frac{\sigma}{\sqrt{n}}$. [c]Calculated using $\frac{rate_p}{rate_{max}} \times [carbonate]_{max} = [carbonate]_p$, , with average k_{obs} values and k_{max}= 8.24 x 10^{-8} s^{-1} (the average k_{obs} in the zero order regime) [d] Determined using $[cat]_0$ – [carbonate] = [alkoxide] for the 'linear' regime, where $[cat]_0$ = 3.57 x 10^{-3} s^{-1} . [e] Calculated from $K_{eq} = \frac{[carbonate]}{[alkoxide][CO_2]}$.

Average K_{eq} = 0.25 ± 0.02 M^{-1} (first order regime).

Calculation of error in K_{eq} for all the catalysts was completed as detailed below for the example of CHO–CO_2 ROCOP with catalyst **3**:

Supplementary Table 11 Calculation of ΔK_{eq} for CHO–CO_2 ROCOP with catalyst 3.

P (bar)	$[CO_2]$ (M)^a	k_{obs}/ 10^{-5} (s^{-1})^b	Δk_{obs} / 10^{-6} (s^{-1})^b	[carbonate] / 10^{-3} (M)^c	Δ[carbonate] / 10^{-4} (M)^c	[alkoxide] / 10^{-3} (M)^d	Δ[alkoxide] / 10^{-4} (M)^d	K_{eq} (M^{-1})^e	ΔK_{eq} (M^{-1})^e
2	0.15	1.31	0.11	0.75	0.41	1.75	0.41	2.86	0.17
4	0.30	1.81	0.85	1.04	0.74	1.46	0.74	2.36	0.21
5	0.38	2.64	0.18	1.51	0.82	0.99	0.82	4.08	0.41
7	0.53	3.72	2.30	2.13	N/A	N/A	N/A	N/A	N/A
10	0.74	4.00	0.78	2.29	N/A	N/A	N/A	N/A	N/A
15	1.41	4.41	5.06	2.53	N/A	N/A	N/A	N/A	N/A
20	2.48	4.64	2.23	2.66	N/A	N/A	N/A	N/A	N/A
25	3.38	4.44	0.74	2.55	N/A	N/A	N/A	N/A	N/A
30	4.26	4.31	0.04	2.47	N/A	N/A	N/A	N/A	N/A

^{a}Calculated from data supplied by Tassaing and co-workers[14]. ^{b}Averages of n = 2 runs, with an error of $\Delta x = \frac{\sigma}{\sqrt{n}}$. ^{c}Calculated using $\frac{rate_p}{rate_{max}} \times [carbonate]_{max} = [carbonate]_p$, , with average k_{obs} values and k_{max}= 4.36 x 10^{-5} s^{-1} (the average k_{obs} in the zero order regime), with an error of $\Delta x = x \times \sqrt{\left(\frac{\Delta k_{obs}}{k_{obs}}\right)^2 + \left(\frac{\Delta k_{max}}{k_{max}}\right)^2}$, where Δk_{max} is calculated with $\frac{\sigma}{\sqrt{n}}$. ^{d}Determined using $[cat]_0$ – [carbonate] = [alkoxide] for the 'linear' regime, where $[cat]_0$ = 2.50 x 10^{-3} s^{-1}, with an error of Δ[alkoxide] = $\sqrt{(\Delta[cat]_0)^2 + (\Delta[carbonate])^2}$= Δ[carbonate] (assuming $(\Delta[cat]_0)$=0), ^{e}Calculated from K_{eq} $\frac{[carbonate]_p}{[alkoxide]_p[CO_2]}$, with an error of $\Delta x = x \times \sqrt{\left(\frac{\Delta[alkoxide]}{[alkoxide]}\right)^2 + \left(\frac{\Delta[carbonate]}{[carbonate]}\right)^2}$

The final error in K_{eq} is taken as the average of the errors for each K_{eq} value used to calculate the final K_{eq}, i.e. here the average of the values in rows 1-3 (= 0.26 M^{-1}).

Supplementary Table 12 Comparison of the equilibrium constants reported in this work, in units of concentration and in units of pressure.

Catalyst	K_{eq} (M^{-1})^a	K_{eq} (bar^{-1})^b
1	0.38 ± 0.08	0.08 ± 0.02
2	0.62 ± 0.09	0.13 ± 0.02
3	3.10 ± 0.26	0.23 ± 0.03
4	0.25 ± 0.02	0.05 ± 0.01
5	1.27 ± 0.23	0.23 ±0.03

$^a K_{eq}$ in M^{-1} was calculated as outlined in Supplementary Tables 7-10. $^b K_{eq}$ in bar^{-1} was calculated according to $K_{eq} = \frac{[carbonate]}{[alkoxide]\ P_{CO2}}$, using the data presented in Supplementary Tables 7-10.

The relationship between K_{eq} in units of pressure (bar^{-1}) or concentration (M^{-1}) is linear, reflecting experimental work to investigate the concentration of CO_2 in epoxide by Foltran and co-workers,[14] as well as work conducted using the experimental set-up presented in this work (Supplementary Figure 51). Additionally, studies investigating solubility of CO_2 in neat CHO and in a 1:4000 solution of catalyst:CHO suggest that under these experimental conditions, the presence of catalyst does not impact CO_2 concentration (Supplementary Figure 51).

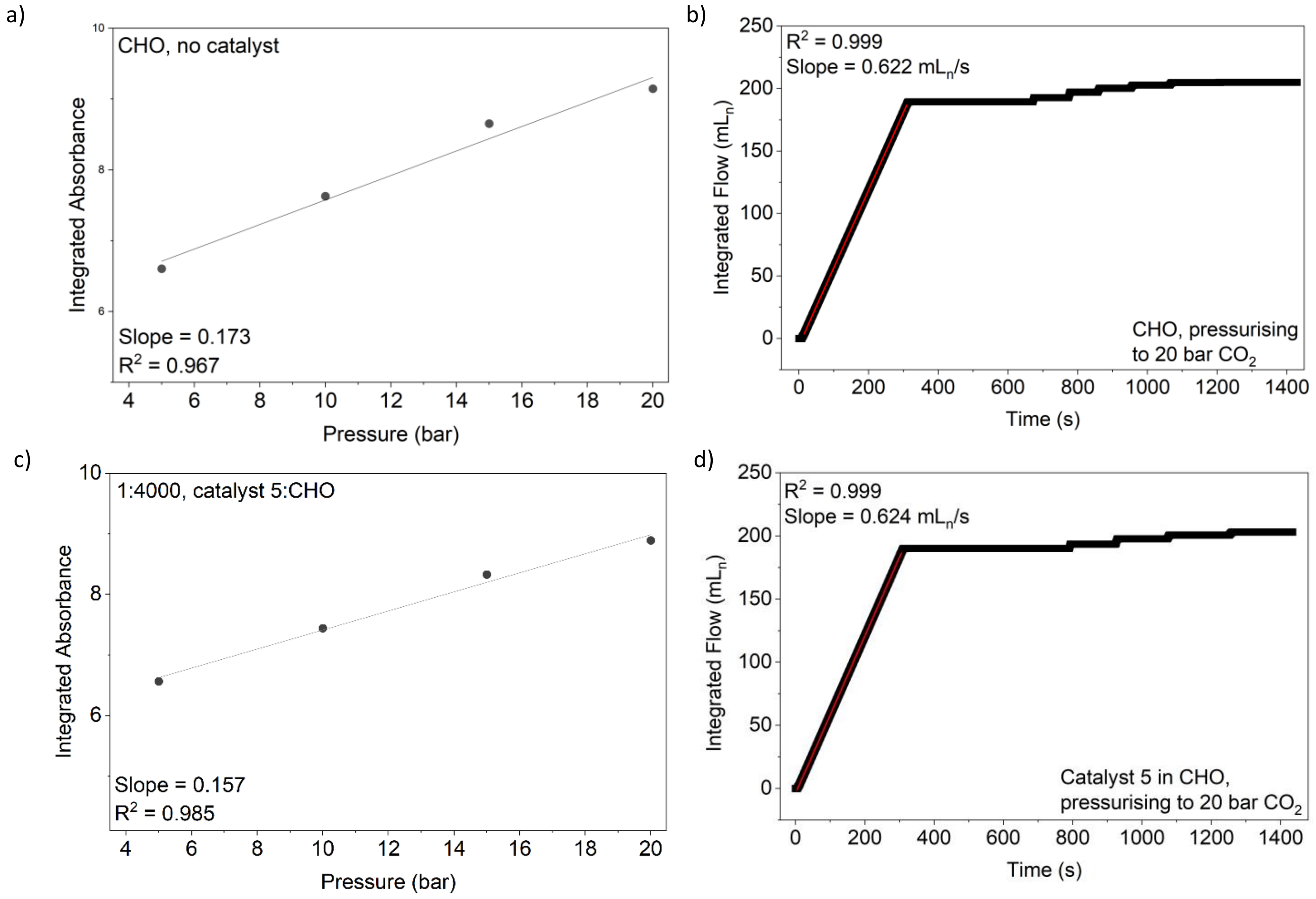

Supplementary Figure 51 a) Plot of the IR absorbance peak area at 2336 cm^{-1} obtained after CO_2 saturation of CO_2 in CHO at pressures of 4 bar, 5 bar, 10 bar, 15 bar and 20 bar CO_2 *vs*. CO_2 pressure. b) Integrated mass flow *vs*. time when pressurizing neat CHO to 20 bar CO_2. c) Plot of the IR absorbance peak area at 2336 cm^{-1} obtained after CO_2 saturation in 1:4000 catalyst 5:CHO, at pressures of 4 bar, 5 bar, 10 bar, 15 bar and 20 bar CO_2 *vs*. CO_2 pressure. d) Integrated mass flow *vs*. time when pressurising a solution of 1:4000 catalyst 5:CHO to 20 bar CO_2.

Subscript 'n' on the units of CO_2 volume (mL) indicates that the mass flow controller instrument converts mass flow rate to volumetric flow rate using the fluid density at a temperature of 0 °C and pressure of 1 atm (European normal reference conditions). The slope of plots in Supplementary Figures 51c and 51d indicates the rate of pressurization, controlled by the mass flow controller, independent of the contents of the reactor vessel.

Michaelis Menten Kinetic Model for Catalyst 2

Through consideration of the Michaelis Menten Model used for enzyme saturation kinetic treatments, a non-linear fitting of the rate data for catalyst **2** against [CO_2] allows for determination of the Michaelis Menten constant, K_M, and maximum rate, v_{max}:

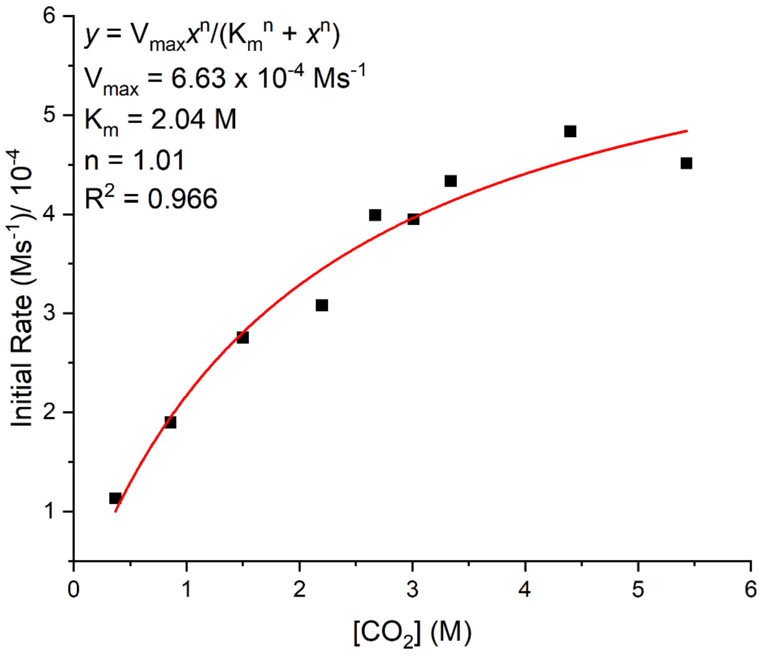

Supplementary Figure 52 Non-linear Fitting of Polymerization Rate vs [CO_2] to Obtain K_{eq}.

The here determined K_m may be approximated by the dissociation constant, K_d, and hence the association constant, K_a, may be determined as the reciprocal of this value.

$K_a = 0.49\ M^{-1}$, in good agreement with the K_{eq} value of $0.62 \pm 0.09\ M^{-1}$.

The value for v_{max} is in good agreement to the maximum rate value from the polymerization rate data, as taking the k_{max} determined for PO–CO_2 ROCOP with catalyst 2 ($4.07 \times 10^{-5}\ s^{-1}$, Figure 3D), and applying the rate law:

$v_{max} = k_{max}[PO]$

Where [PO] = 14.3 M, leads to a value of $v_{max} = 5.82 \times 10^{-4}\ M\ s^{-1}$, in good agreement with the saturation kinetics treatment value of $6.63 \times 10^{-4}\ M\ s^{-1}$.

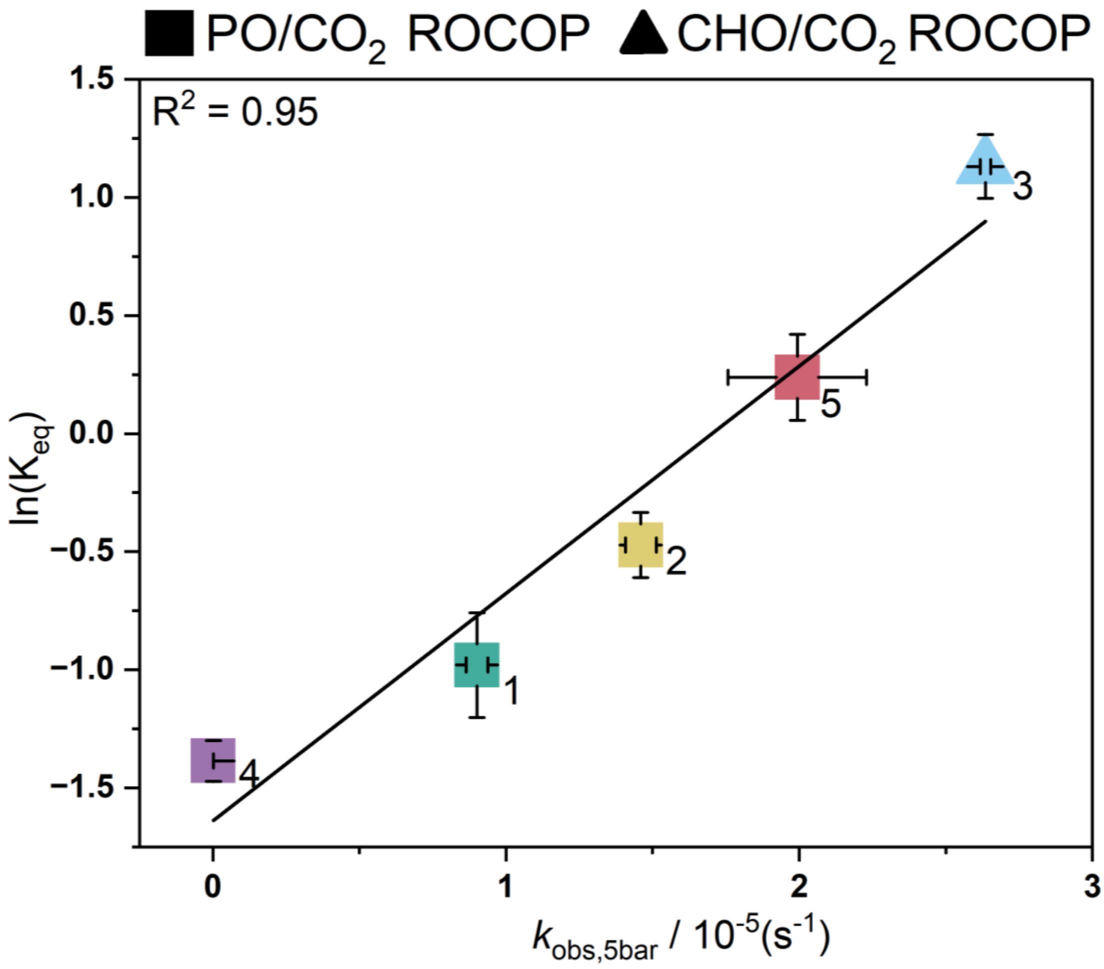

Supplementary Figure 53 Plot of ln(K_{eq}) vs. $k_{obs,5bar}$. All ln(k_{obs}) values were determined from k_{obs} values obtained as the average of n=2 independent runs, with errors indicated as ± the standard error from the mean. Values for K_{eq} and corresponding errors were calculated as indicated in Supplementary Tables 7-11, and values for ln(K_{eq}) were obtained from these values, with errors propagated accordingly.

Prediction of Equilibrium Parameters for Further Catalysts

Catalysts **6** and **8** were synthesized according to the literature procedures (Supplementary Figure 53).[15,16]

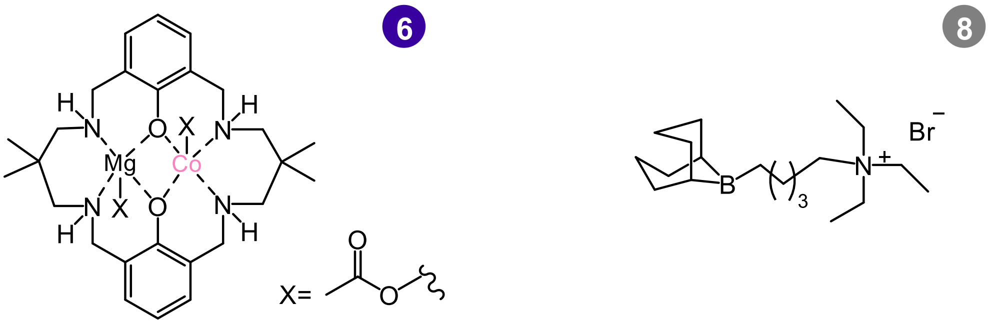

Supplementary Figure 54 Structures of Catalysts 6 and 8, synthesized according to literature procedures.

Supplementary Table 13 Polymerization Rate Data for Prediction of $P_{threshold}$ and K_{eq} using Catalysts 1, 2, 5, 6 and 8 for CHO–CO_2 ROCOP.

Catalyst	$k_{obs,5bar}$/ 10^{-5} (s^{-1})	Predicted $P_{threshold}$ (bar)[a]	$k_{obs, Pthreshold}$ /10^{-5} (s^{-1})	$k_{obs, 20 bar}$/ 10^{-5} (s^{-1})	Predicted K_{eq}[b]
**1**	0.293	16	0.85	0.86	0.27
**2**	3.86	< 5	3.86	3.91	23.4
**5**	1.68	14	3.90	4.10	0.85
**6**	0.345	16	0.91	0.77	0.28
**8***	0.015	-	0.022	0.031	-

[a]$P_{threshold}$ was predicted from the $k_{obs,5bar}$ values using the correlation identified in Figure 4D: y = 16.35 – 0.43exp(x/8.55 x 10^{-6}). [b]K_{eq} was predicted from the $k_{obs,5bar}$ values using the correlation identified in Figure 4B: y = 0.21 + 0.04exp(164818x). The rate data, k_{obs}, was obtained at 50 °C, using catalyst (0.025 mol%, 2.5 mM), CHO (6 mL, 9.9 M), *trans*-1,2-cyclohexanediol (0.5 mol%, 71 mM). *The tested organoborane catalyst showed very low activity across all pressures tested (5, 16 and 20 bar), and so no further analysis was conducted.

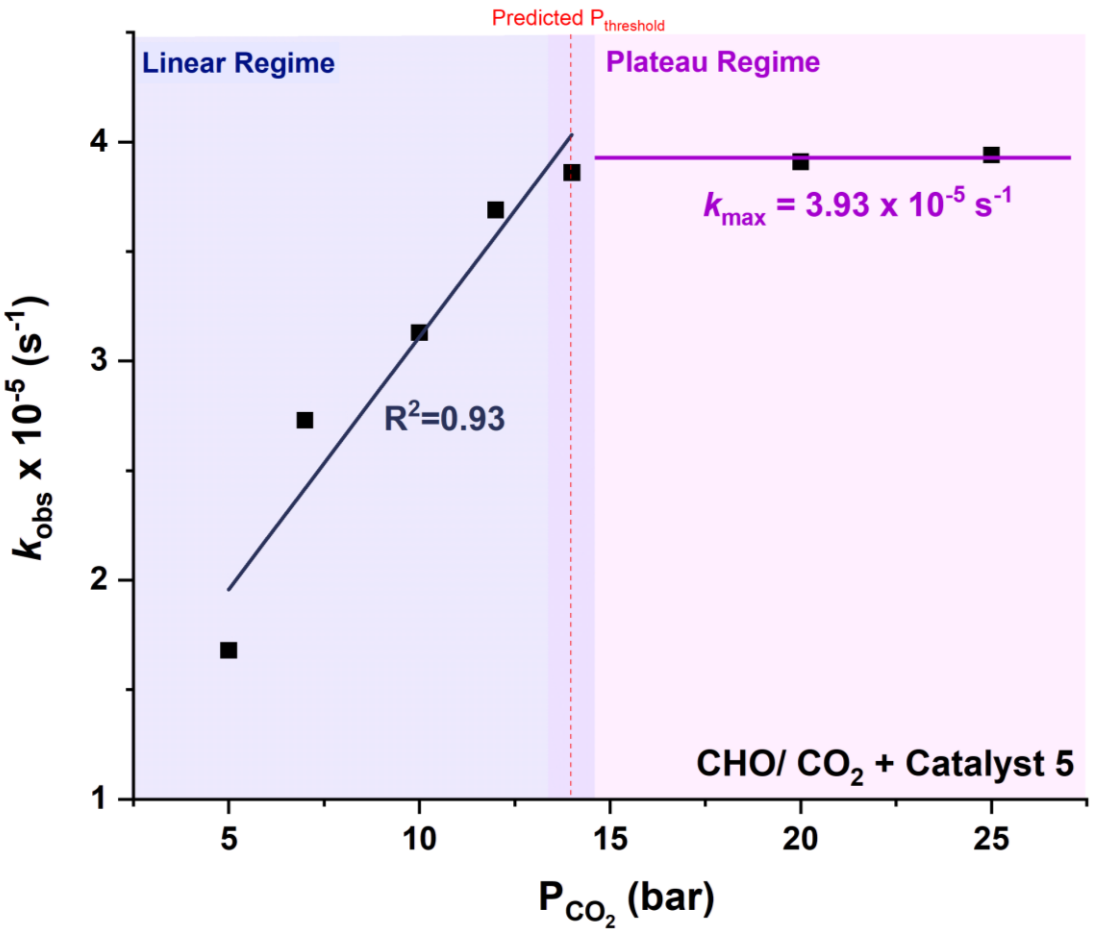

Supplementary Figure 55 k_{obs} *vs*. **P(CO_2) for CHO–CO_2 ROCOP with catalyst 5, identifying $P_{threshold}$ = 14 bar CO_2.**

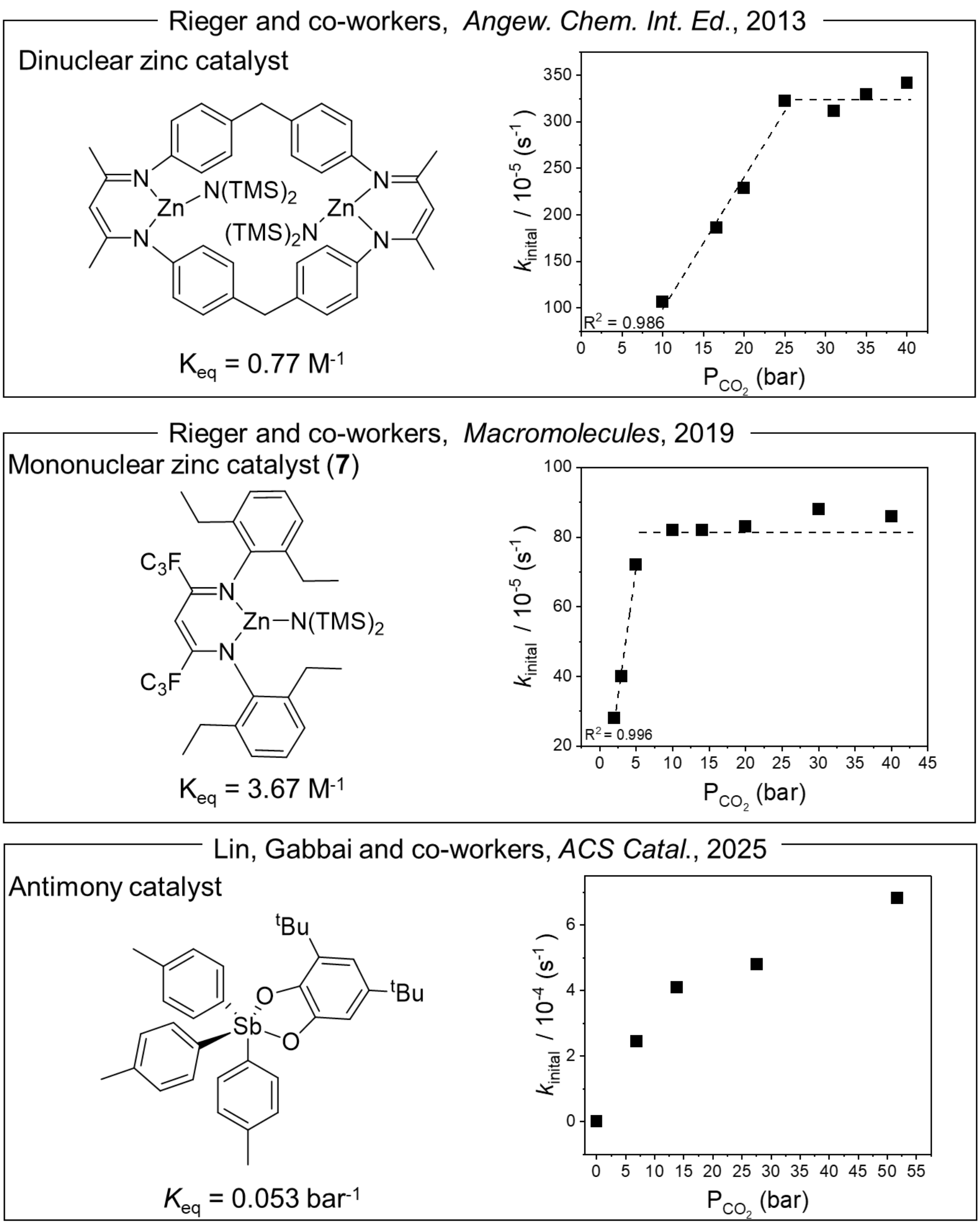

Supplementary Figure 56 Three additional catalysts, reported in the literature, for which experimental evidence for a CO_2 insertion equilibrium have been reported.

Top: Di-Zn β-diiminate catalyst, reported for CHO–CO_2 ROCOP by Rieger and co-workers. This catalyst also showed evidence of pressure dependent activity with $P_{Threshold}$ observed at 25 bar and K_{eq} = 0.77 M^{-1}.[3,17,18] Unfortunately, in the previously report it is not clear which temperature was used, hence this data is not included in Figure 6. Middle: Zn(II) β-diiminate catalyst (7), reported by Rieger and co-workers, where K_{eq} = 3.67 M^{-1} for [Zn]:[CHO] = 1:160, 60 °C, 6.31 mL neat CHO. Bottom: Antimony catalyst, reported by Lin, Gabbai and co-workers, where K_{eq} = 0.53 MPa^{-1}.[19] The $P_{Threshold}$ was not achieved for this catalyst, even at 55 bar, hence it is not included in Figure 6.

Higher Pressure Investigation of PO–CO_2 ROCOP using Catalyst 5

Supplementary Table 14 Polymerization Rate Data for PO–CO_2 ROCOP using Catalyst 5, for pressures above and including the identified $P_{threshold}$ value.

Pressure/ bar	k_{obs} x 10^{-5}/ s^{-1} ^{a}	Polycarbonate selectivity (%) ^{b}
12[20]	3.26 ± 0.04	98
15[20]	3.62 ± 0.01	98
20[20]	3.44 ± 0.01	99
25[20]	3.09 ± 0.03	97
30[20]	3.59 ± 0.12	98
35	4.33 ± 0.43	97
40	3.81 ± 0.38	96

Polymerization conditions: 50 ˚C, catalyst (0.025 mol%, 3.6 mM), PO (6 mL, 14.3 M), trans-1,2-cyclohexanediol (0.5 mol%, 71 mM) at fixed CO_2 pressure. $^{e}k_{obs}$ determined as the gradient of the plot of $\ln[PO]_t/[PO]_0$ *vs.* time. ^{b}Polycarbonate selectivity was determined by dividing the sum of ^{1}H NMR integrals for polycarbonate and any ether by the sum of integrals for polycarbonate, cyclic carbonate and ether, where integrals were identified for peaks of PPC (4.92 ppm, 1H), PC (4.77 ppm, 1H) and poly(propylene oxide) (3.46-3.64 ppm, 3H) against mesitylene (0.25 mol%, 36 mM) as an internal standard. *Error in the determination of k_{obs} for polymerizations conducted at 35 bar and 40 bar is taken as a representative error for polymerizations at these pressure ranges (±10%).

References

1 Cohen, C. T., Chu, T. & Coates, G. W. Cobalt Catalysts for the Alternating Copolymerization of Propylene Oxide and Carbon Dioxide: Combining High Activity and Selectivity. *J. Am. Chem. Soc.* **127**, 10869–10878 (2005).

2 Deacy, A. C., Moreby, E., Phanopoulos, A. & Williams, C. K. Co(III)/Alkali-Metal(I) Heterodinuclear Catalysts for the Ring-Opening Copolymerization of CO2 and Propylene Oxide. *J. Am. Chem. Soc.* **142**, 19150–19160 (2020).

3 Eisenhardt, K. H. S., Fiorentini, F., Lindeboom, W. & Williams, C. K. Quantifying CO2 Insertion Equilibria for Low-Pressure Propene Oxide and Carbon Dioxide Ring Opening Copolymerization Catalysts. *J. Am. Chem. Soc.* **146**, 10451–10464 (2024).

4 Chen, Z. *et al.* Environmental impacts and mitigation potentials of CO2-based biodegradable plastic based on life cycle assessment – A case study of poly(propylene carbonate). *J. Clean. Prod.* **471**, 143387 (2024).

5 Nagae, H., Matsushiro, S., Okuda, J. & Mashima, K. Cationic tetranuclear macrocyclic CaCo3 complexes as highly active catalysts for alternating copolymerization of propylene oxide and carbon dioxide. *Chem. Sci.* **14**, 8262–8268 (2023).

6 Akine, S., Taniguchi, T. & Nabeshima, T. Helical Metallohost−Guest Complexes via Site-Selective Transmetalation of Homotrinuclear Complexes. *J. Am. Chem. Soc.* **128**, 15765–15774 (2006).

7 Nakano, K., Kobayashi, K. & Nozaki, K. Tetravalent Metal Complexes as a New Family of Catalysts for Copolymerization of Epoxides with Carbon Dioxide. *J. Am. Chem. Soc.* **133**, 10720–10723 (2011).

8 Nakano, K., Kamada, T. & Nozaki, K. Selective Formation of Polycarbonate over Cyclic Carbonate: Copolymerization of Epoxides with Carbon Dioxide Catalyzed by a Cobalt(III) Complex with a Piperidinium End-Capping Arm. *Angew. Chem., Int. Ed.* **45**, 7274–7277 (2006).

9 Wang, Y., Liu, Z., Guo, W., Zhang, C. & Zhang, X. Phosphine-Borane Frustrated Lewis Pairs for Metal-Free CO2/Epoxide Copolymerization. *Macromolecules* (2023).

10 Zhou, Z. *et al.* Dynamic Foldamer Catalyst Enables Efficient Copolymerization of CO2 and Epoxides. *ACS Catal.* **13**, 15116–15125 (2023).

11 Wu, W., Qin, Y., Wang, X. & Wang, F. New bifunctional catalyst based on cobalt–porphyrin complex for the copolymerization of propylene oxide and CO2. *J. Polym. Sci., Part A: Polym. Chem.* **51**, 493–498 (2013).

12 Darensbourg, D. J., Yarbrough, J. C., Ortiz, C. & Fang, C. C. Comparative Kinetic Studies of the Copolymerization of Cyclohexene Oxide and Propylene Oxide with Carbon Dioxide in the Presence of Chromium Salen Derivatives. In Situ FTIR Measurements of Copolymer vs Cyclic Carbonate Production. *J. Am. Chem. Soc.* **125**, 7586–7591 (2003).

13 Olsson, J. V., Hult, D., Cai, Y., García-Gallego, S. & Malkoch, M. Reactive imidazole intermediates: simplified synthetic approach to functional aliphatic cyclic carbonates. *Polym. Chem.* **5**, 6651–6655 (2014).

14 Stéphanie Foltran, E. C., Henri Cramail, Thierry Tassaing. In situ FTIR investigation of the solubility and swelling of model epoxides in supercritical CO2. *J. Supercrit. Fluids.* **63**, 52–58 (2012).

15 Deacy, A. C., Kilpatrick, A. F. R., Regoutz, A. & Williams, C. K. Understanding metal synergy in heterodinuclear catalysts for the copolymerization of CO2 and epoxides. *Nat. Chem.* **12**, 372–380 (2020).

16 Yang, G.-W., Zhang, Y.-Y., Xie, R. & Wu, G.-P. Scalable Bifunctional Organoboron Catalysts for Copolymerization of CO2 and Epoxides with Unprecedented Efficiency. *J. Am. Chem. Soc.* **142**, 12245–12255 (2020).

17 Lehenmeier, M. W. *et al.* Flexibly Tethered Dinuclear Zinc Complexes: A Solution to the Entropy Problem in CO2/Epoxide Copolymerization Catalysis? *Angew. Chem.- Int. Ed.* **52**, 9821–9826 (2013).

18 Kernbichl, S., Reiter, M., Mock, J. & Rieger, B. Terpolymerization of β-Butyrolactone, Epoxides, and CO2: Chemoselective CO2-Switch and Its Impact on Kinetics and Material Properties. *Macromolecules* **52**, 8476–8483 (2019).

19 Jiang, C. *et al.* Pnictogen-Bonding Catalysis: Copolymerization of CO2 and Epoxides on Antimony(V) Platforms. *ACS Catal.* **15**, 17882–17892 (2025).

20 Eisenhardt, K. H. S., Fiorentini, F., Lindeboom, W. & Williams, C. K. Quantifying CO2 Insertion Equilibria for Low-Pressure Propene Oxide and Carbon Dioxide Ring Opening Copolymerization Catalysts. *J. Am. Chem. Soc.* **146**, 10451–10464 (2024).
